# Clinical validation of deep learning algorithms for radiotherapy targeting of non-small-cell lung cancer: an observational study

**DOI:** 10.1016/S2589-7500(22)00129-7

**Published:** 2022-09

**Authors:** Ahmed Hosny, Danielle S Bitterman, Christian V Guthier, Jack M Qian, Hannah Roberts, Subha Perni, Anurag Saraf, Luke C Peng, Itai Pashtan, Zezhong Ye, Benjamin H Kann, David E Kozono, David Christiani, Paul J Catalano, Hugo J W L Aerts, Raymond H Mak

**Affiliations:** Artificial Intelligence in Medicine Program, Mass General Brigham; Department of Radiation Oncology, Brigham and Women’s Hospital and Dana-Farber Cancer Institute; Artificial Intelligence in Medicine Program, Mass General Brigham; Department of Radiation Oncology, Brigham and Women’s Hospital and Dana-Farber Cancer Institute; Harvard Medical School, Boston, MA, USA; Computational Health Informatics Program, Boston Children’s Hospital, Boston, MA; Department of Radiation Oncology, Brigham and Women’s Hospital and Dana-Farber Cancer Institute; Harvard Radiation Oncology Program, Brigham and Women’s Hospital, Dana-Farber Cancer Institute, Mass General Brigham, Boston, MA; Harvard Radiation Oncology Program, Brigham and Women’s Hospital, Dana-Farber Cancer Institute, Mass General Brigham, Boston, MA; Harvard Radiation Oncology Program, Brigham and Women’s Hospital, Dana-Farber Cancer Institute, Mass General Brigham, Boston, MA; Harvard Radiation Oncology Program, Brigham and Women’s Hospital, Dana-Farber Cancer Institute, Mass General Brigham, Boston, MA; Harvard Radiation Oncology Program, Brigham and Women’s Hospital, Dana-Farber Cancer Institute, Mass General Brigham, Boston, MA; Department of Radiation Oncology, Brigham and Women’s Hospital and Dana-Farber Cancer Institute; Artificial Intelligence in Medicine Program, Mass General Brigham; Department of Radiation Oncology, Brigham and Women’s Hospital and Dana-Farber Cancer Institute; Artificial Intelligence in Medicine Program, Mass General Brigham; Department of Radiation Oncology, Brigham and Women’s Hospital and Dana-Farber Cancer Institute; Department of Radiation Oncology, Brigham and Women’s Hospital and Dana-Farber Cancer Institute; Harvard T H Chan School of Public Health, Massachusetts General Hospital and Harvard Medical School; Department of Radiation Oncology and Molecular Radiation Sciences, Johns Hopkins University School of Medicine, Baltimore, MD, USA; Artificial Intelligence in Medicine Program, Mass General Brigham; Department of Radiation Oncology, Brigham and Women’s Hospital and Dana-Farber Cancer Institute; Radiology and Nuclear Medicine, CARIM & GROW, Maastricht University, Maastricht, Netherlands; Artificial Intelligence in Medicine Program, Mass General Brigham; Department of Radiation Oncology, Brigham and Women’s Hospital and Dana-Farber Cancer Institute

## Abstract

**Background:**

Artificial intelligence (AI) and deep learning have shown great potential in streamlining clinical tasks. However, most studies remain confined to in silico validation in small internal cohorts, without external validation or data on real-world clinical utility. We developed a strategy for the clinical validation of deep learning models for segmenting primary non-small-cell lung cancer (NSCLC) tumours and involved lymph nodes in CT images, which is a time-intensive step in radiation treatment planning, with large variability among experts.

**Methods:**

In this observational study, CT images and segmentations were collected from eight internal and external sources from the USA, the Netherlands, Canada, and China, with patients from the Maastro and Harvard-RT1 datasets used for model discovery (segmented by a single expert). Validation consisted of interobserver and intraobserver benchmarking, primary validation, functional validation, and end-user testing on the following datasets: multi-delineation, Harvard-RT1, Harvard-RT2, RTOG-0617, NSCLC-radiogenomics, Lung-PET-CT-Dx, RIDER, and thorax phantom. Primary validation consisted of stepwise testing on increasingly external datasets using measures of overlap including volumetric dice (VD) and surface dice (SD). Functional validation explored dosimetric effect, model failure modes, test-retest stability, and accuracy. End-user testing with eight experts assessed automated segmentations in a simulated clinical setting.

**Findings:**

We included 2208 patients imaged between 2001 and 2015, with 787 patients used for model discovery and 1421 for model validation, including 28 patients for end-user testing. Models showed an improvement over the interobserver benchmark (multi-delineation dataset; VD 0·91 [IQR 0·83–0·92], p=0·0062; SD 0·86 [0·71–0·91], p=0·0005), and were within the intraobserver benchmark. For primary validation, AI performance on internal Harvard-RT1 data (segmented by the same expert who segmented the discovery data) was VD 0·83 (IQR 0·76–0·88) and SD 0·79 (0·68–0·88), within the interobserver benchmark. Performance on internal Harvard-RT2 data segmented by other experts was VD 0·70 (0·56–0·80) and SD 0·50 (0·34–0·71). Performance on RTOG-0617 clinical trial data was VD 0·71 (0·60–0·81) and SD 0·47 (0·35–0·59), with similar results on diagnostic radiology datasets NSCLC-radiogenomics and Lung-PET-CT-Dx. Despite these geometric overlap results, models yielded target volumes with equivalent radiation dose coverage to those of experts. We also found non-significant differences between de novo expert and AI-assisted segmentations. AI assistance led to a 65% reduction in segmentation time (5·4 min; p<0·0001) and a 32% reduction in interobserver variability (SD; p=0·013).

**Interpretation:**

We present a clinical validation strategy for AI models. We found that in silico geometric segmentation metrics might not correlate with clinical utility of the models. Experts’ segmentation style and preference might affect model performance.

## Introduction

Lung cancer is the leading cause of cancer-related mortalities worldwide.^1^ Non-small-cell lung cancer (NSCLC) is the most common type of lung cancer, accounting for 85% of all diagnoses.^2^ Radiotherapy plays a key role in treating NSCLC, with one fifth of early-stage and half of late-stage patients receiving this treatment modality.^3^ Radiotherapy can be administered as a sole treatment, with systemic agents, precede or follow surgery, and play a role in palliation.

Radiotherapy’s time-effectiveness and cost-effectiveness is affected by an expensive upfront investment: radiotherapy planning. Radiotherapy planning is crucial in maximising radiation to cancer tissue and minimising radiation to normal tissue. After image acquisition, planning steps include image registration, target and adjacent organ segmentation, and dose distribution. The manual segmentation of the target—ie, primary tumour and involved lymph nodes—is one of the most time-consuming planning tasks done by radiation oncologists.^4^ This meticulous task requires interpreting images on a voxel-by-voxel basis to delineate the target volume. The advent of advanced radiotherapy planning and delivery techniques such as intensity-modulated radiotherapy and image guidance have enabled smaller doses to surrounding organs, but require high segmentation accuracy.^5^ Additionally, a large and well documented interobserver variability exists in target segmentation,^6,7^ even in radiotherapy clinical trials with prespecified parameters.^8^ Finally, the accuracy of target segmentation can directly affect patient outcomes, with under-segmentation decreasing tumour control and over-segmentation increasing toxicity risks.^9^

Several computer-aided tools have been proposed to help streamline radiotherapy planning.^4^ For segmentation tasks, semi-automated approaches, including segmentation atlases, have had varying degrees of clinical utility.^10^ Curating atlases requires substantial time and effort on the physician’s part, and the heterogeneity of their contents might diminish performance. More recently, artificial intelligence (AI) methods—deep learning, specifically—have been proposed as promising alternatives.^11^ Deep learning algorithms can automatically learn feature representations from data, improving performance across multiple tasks.^12^ Although studies have explored the use of deep learning to automate radiotherapy target segmentation and improve its accuracy and consistency,^13^ most remain at the proof-of-concept stage. As such, these studies are often confined to in silico validation in small internal cohorts without external validation. Among many promising results, only a few efforts show the clinical impact of these automated systems.^14,15^

**Figure 1:**
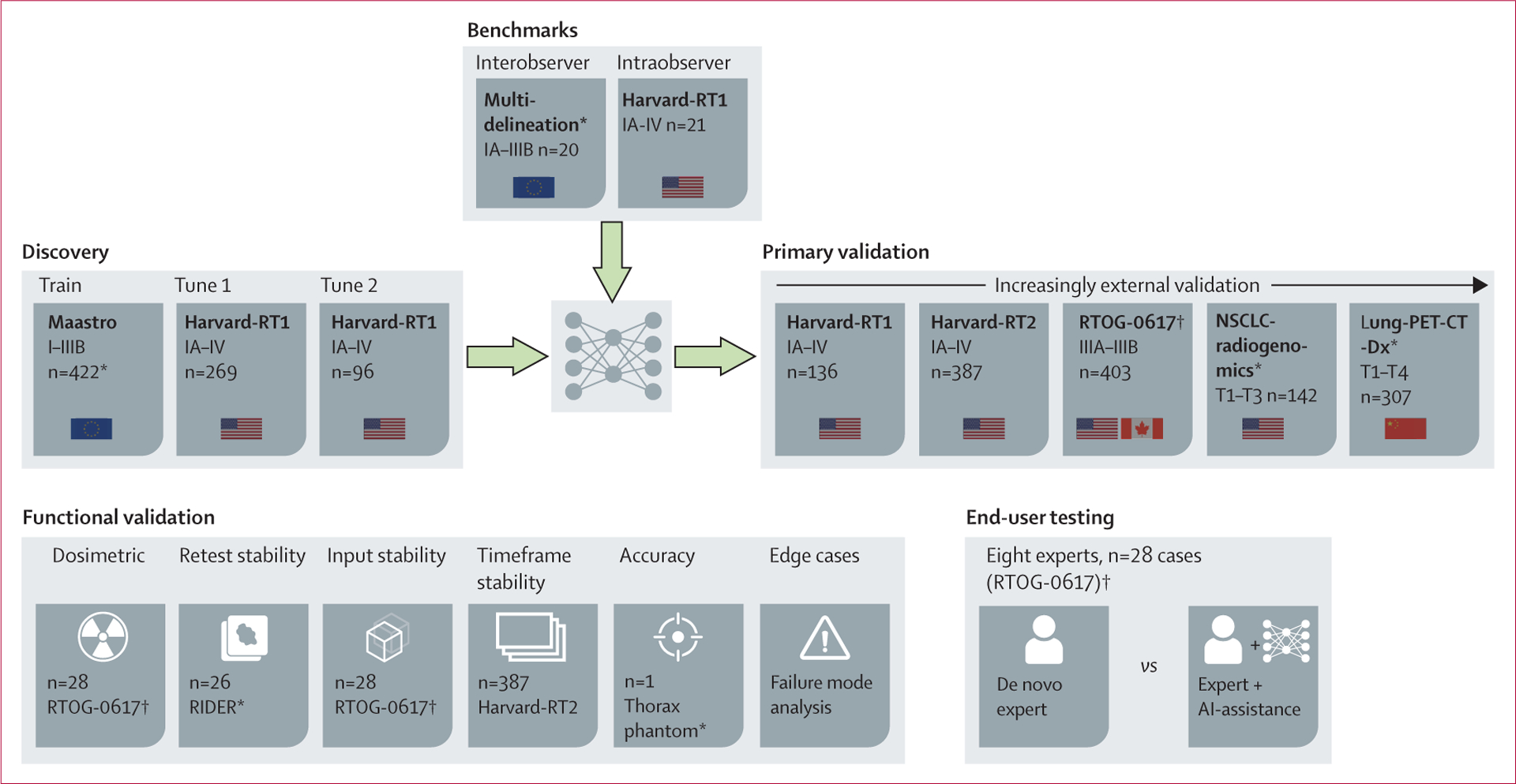
Clinical validation framework and experimental setup AI=artificial intelligence. *Publicly available. †Limited access.

In this study, we present a generalisable clinical validation strategy for therapeutic AI algorithms with the aim of bridging early proof-of-concept studies and prospective clinical trials. The strategy comprises four main components: benchmarks, primary validation, functional validation, and end-user testing (figure 1). To show the application of this strategy, we present a study in clinically validating deep learning models for radiotherapy targeting for NSCLC.

## Methods

### Discovery data

We used Maastro and Harvard-RT1 datasets for model development. Maastro included 422 patients (stages I–IIIB; 290 [69%] male, 132 [31%] female; mean age 68 years [range 33–91]) with histologically proven NSCLC and treated with radiotherapy alone (n=196 [46%]) or with radiotherapy with chemotherapy (n=226 [54%]). Patients were treated at Maastro Clinic, Maastricht, Netherlands, between 2004 and 2010 (appendix p 16).

Harvard-RT1 included 501 patients (stages IA–IV; 263 [52%] male, 236 [47%] female, 2 [<1%] unspecified; median age 73 years [range 39–89]) with histologically proven NSCLC referred for radiotherapy between 2001 and 2015 at the Dana-Farber Cancer Institute and Brigham and Women’s Hospital, Boston, MA, USA. Target volumes were delineated by a single radiation oncologist (RHM; referred to as R1). 269 (54%) patients from this dataset were used for training, 96 (19%) for tuning, and 136 (27%) for testing. The test set was identical to that used in a published study, in which contestants competed to develop the best segmentation model.^16^ Using volumetric dice (VD) and surface dice (SD) metrics, the top solution from that contest was compared to results obtained here.

### Data preprocessing

Data preprocessing involved resampling all data to a common voxel spacing of 1*1*3 mm^3^, using linear interpolations for CT images and nearest-neighbour interpolations for segmentations. CT Hounsfield units were normalised by clipping to 0·5th and 99·5th percentiles. Distributions of dataset characteristics, including gross tumour volume, CT image slice thickness, and use of intravenous contrast are given in the appendix (pp 62, 64–65). Data augmentation details during training are also given in the appendix (p 6).

### Model development

**Figure 2:**
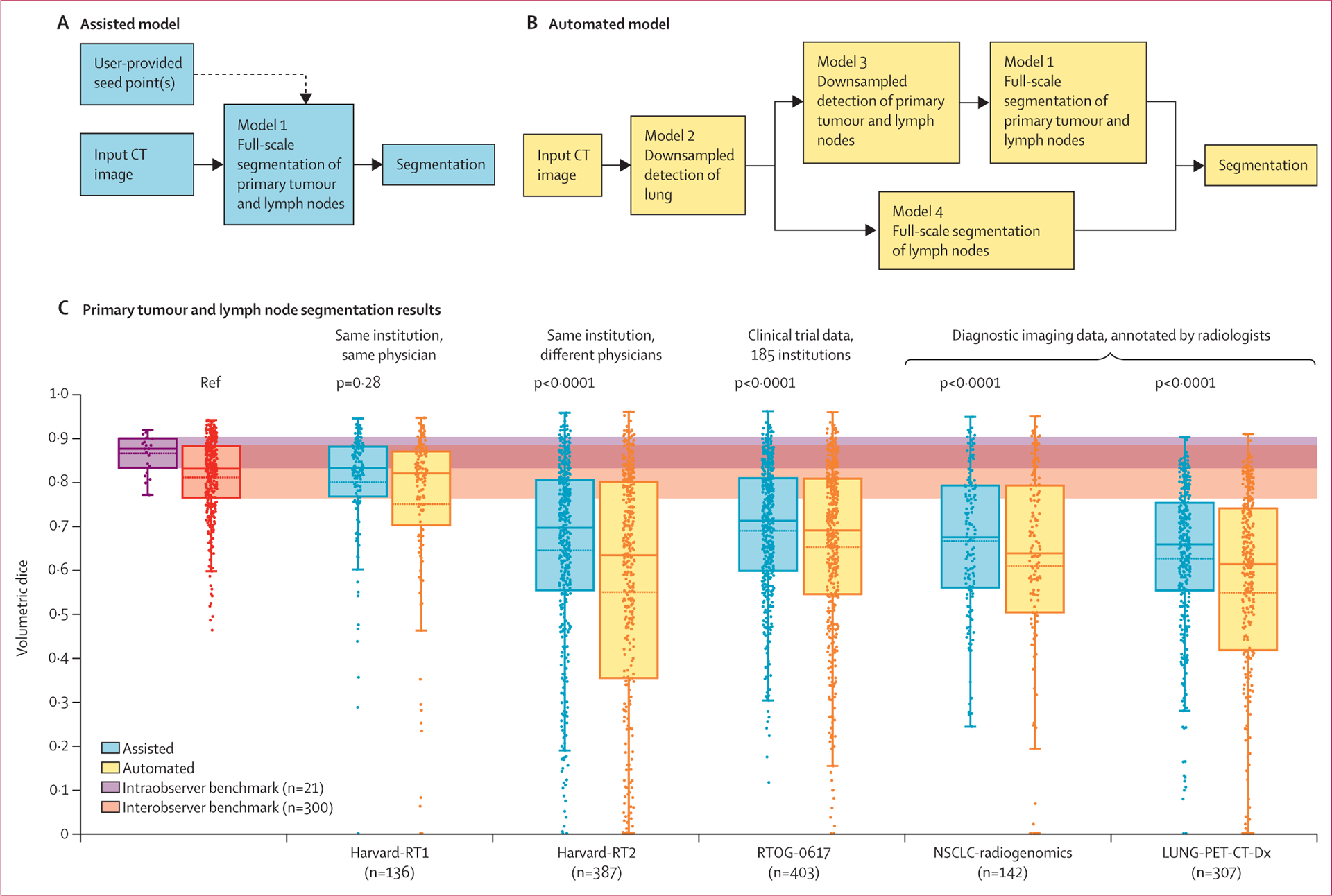
Primary validation results and comparison with benchmarks Schematics for the assisted (A) and automated (B) segmentation pipelines. (C) The model performance in localising and segmenting primary non-small-cell lung cancer tumours and involved lymph nodes, as validated on five increasingly external datasets using the volumetric dice metric.

We used U-Nets, fully convolutional neural networks with CT volume inputs and corresponding binary segmentation outputs. Our assisted and automated pipelines consisted of four three-dimensional (3D) U-Net models for the localisation and segmentation of lungs, primary tumour, and involved lymph nodes (figure 2A, B). Overall model structure closely followed that of original implementation^17^ with encoder and decoder paths connected via skip connections. Architecture parameters (eg, convolution and pooling kernel sizes; appendix p 14) and model hyperparameters (eg, learning rate and batch size; appendix pp 6–7) were fine-tuned using nnU-Net (version 1.6.5).^18^

### Metrics

We used multiple metrics to assess model performance (appendix p 15), including VD (spatial overlap of two volumes, with 0 being no overlap and 1 being perfect overlap), as well as SD (fraction of surface within a threshold distance from another, with 0 being none of the surfaces within tolerance and 1 being entire surfaces within tolerance). We used the 75th percentile of interobserver variability (1·9 mm) as the threshold (appendix p 25–26).

### Benchmarks

We developed the interobserver benchmark using the multi-delineation dataset^19^ of 20 patients (stages IA–IIIB; 12 [60%] male, 8 [40%] female; median age 67 years [IQR 57–71]) with histologically confirmed NSCLC referred for radiotherapy at Maastro Clinic. Manual tumour delineations were done by five radiation oncologists, in addition to R1 (appendix pp 18, 68). Comparisons were drawn between the two residents and four attendings involved in the interobserver benchmark. The intraobserver benchmark was developed using 21 randomly sampled patients from the Harvard-RT1 test set. R1 did the segmentation task twice with a 3-month washout period.

### Primary validation

We did further validation on four increasingly external datasets, with increasingly diverging characteristics from those of the training data: Harvard-RT2 segmented by other experts, RTOG-0617 from other institutions, and NSCLC-radiogenomics and Lung-PET-CT-Dx from diagnostic radiology. Harvard-RT2 included 387 patients (stages IA–IV; 165 [43%] male, 222 [57%] female; median age 69 years [range 32–92]) with histologically confirmed NSCLC referred for radiotherapy between 2011 and 2017 at the Dana-Farber Cancer Institute and Brigham and Women’s Hospital. Tumour volumes were delineated by multiple physicians (appendix p 19). RTOG-0617 included 403 patients with histologically confirmed NSCLC (stages IIIA–IIIB; 223 [55%] male, 155 [38%] female, 25 [6%] unspecified; median age 64 years [IQR 57–70]) from the phase 3 RTOG-0617 trial (NCT00533949).^20,21^ Patients were treated with radiotherapy between 2007 and 2011 at 185 institutions across the USA and Canada (appendix pp 20, 69). NSCLC-radiogenomics included 142 patients with histologically confirmed early stage NSCLC (pathological stages T1–3, N0–2, and M0–1; 124 [77%] male, 38 [23%] female; mean age 68 years [range 42–86]) referred for surgical treatment at Stanford University School of Medicine (n=67) and Palo Alto Veterans Affairs (n=75) in California, USA, between 2008 and 2012.^22^ Tumour segmentations were done by two thoracic radiologists (appendix pp 22, 70). Lung-PET-CT-Dx included 307 patients with histologically confirmed NSCLC (clinical stages T1–4, N0–3, and M0–13; 163 [53%] male, 144 [47%] female; mean age 61 years [range 28–90]) imaged at the Second Affiliated Hospital of Harbin Medical University, Harbin, China.^23^ Tumour location was annotated using per-slice bounding rectangles by five thoracic radiologists (appendix pp 23, 71).

### Functional validation

Data used in the dosimetric analysis were from a random quartile-based subset of 28 patients in the RTOG-0617 dataset (appendix p 44). Dose coverage was compared between the planning target volume as used in the RTOG-0617 clinical trial and its AI-generated counterpart, generated from the gross tumour volume (appendix p 7). Two common dose metrics were calculated: V95, percentage target volume receiving at least 95% of the prescription dose; and D95, dose covering 95% of the target volume. Test-retest stability (geometric overlap between segmentations generated from different CT images of the same patient) was assessed using RIDER,^24^ which included 26 patients with NSCLC (primary tumour ≥1 cm; 12 men, 14 women; mean age 62 years [range 29–82]), each of whom underwent two CT scans of the chest within 15 min. Images were acquired between January and September, 2007, at the Memorial Sloan-Kettering Cancer Center, New York, NY, USA. Tumour segmentations were done by two thoracic radiologists (appendix p 72). Additionally, we tested the assisted models’ stability as a function of variation in input data by simulating multiple experts’ placement of seed points (50 simulations). Random seed points were generated within a 50 mm cube around the tumour’s centre of mass, and we measured the geometric overlap between the resultant segmentations. Timeframe stability between 3D and 4D CT data was tested in Harvard-RT2 (n=186 single timeframe 3D CT *vs* n=201 multi-timeframe 4D CT). Model accuracy was assessed using CT of a thorax phantom containing 12 synthetic lung lesions (10 mm and 20 mm in effective diameter, six per lung). The phantom was scanned at Columbia University Medical Center, New York, NY, USA (appendix p 24).^25^ Lesion volume as calculated from our AI-generated segmentations was compared with that of three previously published segmentation models.^26^ Model performance based on the use of intravenous contrast in images (detected using a published algorithm^27^) was assessed through subgroup analysis. DSB conducted the model failure mode analysis by qualitatively assessing model results on the RTOG-0617 dataset and identifying cases of under-segmentation or over-segmentation.

### End-user testing

We recruited and obtained written informed consent (under protocol DF/HCC 20-328) from eight radiation oncologists (DSB, JMQ, HR, SP, AS, LCP, BHK, and RHM) from the Department of Radiation Oncology at Brigham and Women’s Hospital. Experts had varying degrees of experience: three attendings (1, 2, and 9 years of experience) and five residents (appendix pp 9–10). Data used were a random quartile-based subset of 28 patients in the RTOG-0617 dataset, further divided into two groups of 14 patients each (appendix p 44). In the first group, experts were asked to perform the segmentation task de novo. For the second group, experts were asked to rate and edit a provided segmentation while masked to its source. In this second group, for ten patients, automated segmentations from the assisted pipeline were provided (AI assisted). For four patients, clinical segmentations from RTOG-0617 were provided (expert assisted; appendix pp 11, 73). Testing was done in a simulated clinical setting within MIM, the software used for this task at our institution (appendix p 74). Experts were provided with the following information per patient: age, sex, Eastern Cooperative Oncology Group score, histology, stage, and primary tumour lung lobe. Experts were also surveyed before, during, and after reviewing each case. After each case, experts were asked to qualitatively rate the task difficulty and quality of provided segmentations. Time for task completion was recorded automatically in the background. We used time measurements to compare between the de novo, expert-assisted, and AI-assisted groups. Correlation between time and segmentation metrics (VD and SD) was also measured. Details on ethics approval and patient consent for the various datasets are given in the appendix (pp 1–6).

### Statistical analysis

All statistical tests done were non-parametric, with a two-tailed p value of less than 0·05 indicating significance. For two dependent groups, we used the Wilcoxon matched-pairs signed rank test. For two independent groups, we used the Mann-Whitney U rank test. For three or more independent groups, we used the Kruskal-Wallis H-test. For measuring correlation between two groups, we used the Spearman rank-order correlation coefficient. All analyses were done in python (version 3.8.0).

### Role of the funding source

The funders of the study had no role in study design, data collection, data analysis, data interpretation, or writing of the report.

## Results

Interobserver benchmark was VD 0·83 (IQR 0·77–0·88) and SD 0·72 (0·61–0·81; appendix pp 25–26). AI versus R1 yielded VD 0·91 (0·83–0·92) and SD 0·86 (0·71–0·91), a significant improvement over the benchmark with VD (p=0·0062) and SD (p=0·0005; appendix pp 27–28). Additionally, AI versus R1 was found to be inversely correlated with the IQR of variability among all six experts (Spearman’s r=–0·74; p=0·0002; appendix p 29). With AI segmentations as reference, non-significant differences were detected between residents and attendings (appendix p 30).

Intraobserver benchmark was VD 0·88 (IQR 0·83–0**·**90) and SD 0·85 (0·80–0·93; appendix p 31). AI versus R1’s first read yielded VD 0·86 (0·83–0·87) and SD 0·79 (0·72–0·90), with similar results for the second read (appendix p 33). For VD, non-significant differences were observed when both results were compared with the benchmark. For SD, AI versus R1’s first read was non-significantly different than the benchmark, while AI versus R1’s second read was significantly lower (p=0·043; appendix pp 32–33).

First, we tested on the internal Harvard-RT1 dataset, also segmented by R1. Results of the assisted primary tumour segmentation were VD 0·86 (IQR 0·81–0·89) and SD 0·83 (0·73–0·91), a significant improvement over previously published results^16^ (p<0·0001; appendix p 35). Automated primary tumour segmentation results were VD 0·83 (0·77–0·89) and SD 0·80 (0·75–0·83). Results for primary tumour and lymph node segmentation were VD 0·83 (0·76–0·88) and SD 0·79 (0·68–0·88) for the assisted model and VD 0·82 (0**·**70–0.88) and SD 0·74 (0·62–0·83) for the automated model (n=3 [2%] localisation failure; appendix p 8; figure 2C).

Second, we tested the model on the internal Harvard-RT2 dataset segmented by other experts in our institution. Results were VD 0·70 (IQR 0·56–0**·**80) and SD 0·50 (0·34–0·71) for the assisted model and VD 0·63 (0·36–0·80) and SD 0·44 (0·23–0·62) for the automated model (n=40 [10%] localisation failure; appendix p 8; figure 2C).

Third, we tested the model on RTOG-0617 trial data.^21^ Results were VD 0·71 (IQR 0**·**60–0·81) and SD 0·47 (0·35–0·59) for the assisted model, and VD 0·69 (0·54–0·82) and SD 0·44 (0**·**30–0·58) for the automated model (n=2 [0·5%] localisation failure; appendix p 8; figure 2C). We found non-significant differences between trial groups (p=0·47; appendix p 39), as well as between radiotherapy treatment techniques (p=0·24; appendix p 40).

Finally, we tested the model on two diagnostic datasets. For NSCLC-radiogenomics,^22^ results were VD 0·68 (IQR 0·56–0·79) and SD 0·61 (0·37–0.85) for the assisted model, and VD 0·64 (0**·**50–0·79) and SD 0·55 (0·30–0·82) for the automated model (n=9 [6%] localisation failure; appendix p 8; figure 2C). We found non-significant differences between lung lobes (p=0·36; appendix p 41). For Lung-PET-CT-Dx,^23^ results were VD 0·66 (0·55–0·76) and SD 0·31 (0·22–0·43) for the assisted model, and VD 0·61 (0·42–0·74) and SD 0·27 (0·16–0·42) for the automated model (n=27 [9%] localisation failure; appendix p 8; figure 2C).

To assess changes in radiation delivered as a result of using AI-generated segmentations, we did a dosimetric analysis (appendix pp 45–46). We found non-significant differences between clinical and AI planning target volumes across two common dose metrics: V95 (p=0·37) and D95 (p=0·47; appendix p 47).

Model stability across two separate CT scans of the same patient were assessed using RIDER^24^ (appendix p 48). AI versus radiologist on the first scan was non-significantly different from the same comparison on the second scan (VD, p=0·25; and SD, p=0·29; appendix p 49). Radiologists’ variation in tumour volume across the two scans was non-significantly different from that of the AI models (p=0·19; appendix p 50). In terms of model stability as a function of variation in seed point placement, median predictions showed high stability with an IQR of 0·02 for both VD and SD (appendix p 51). With regard to stability across CT timeframes, we found non-significant differences between 3D and 4D CT data (VD, assisted model p=0·14, automated model p=0·33; appendix pp 36–38).

Model accuracy was measured on CT of a thorax phantom containing nodules of known volume^25^ (appendix p 52). On average, models were found to underestimate nodule volume by 0·4 cm^3^, or 12% of known volume. Three published models also showed similar trends on the same data^26^ (appendix p 53). Models were also found to significantly over-perform on contrast-enhanced images (VD p=0·042; SD p=0·0043; appendix p 66).

**Figure 3:**
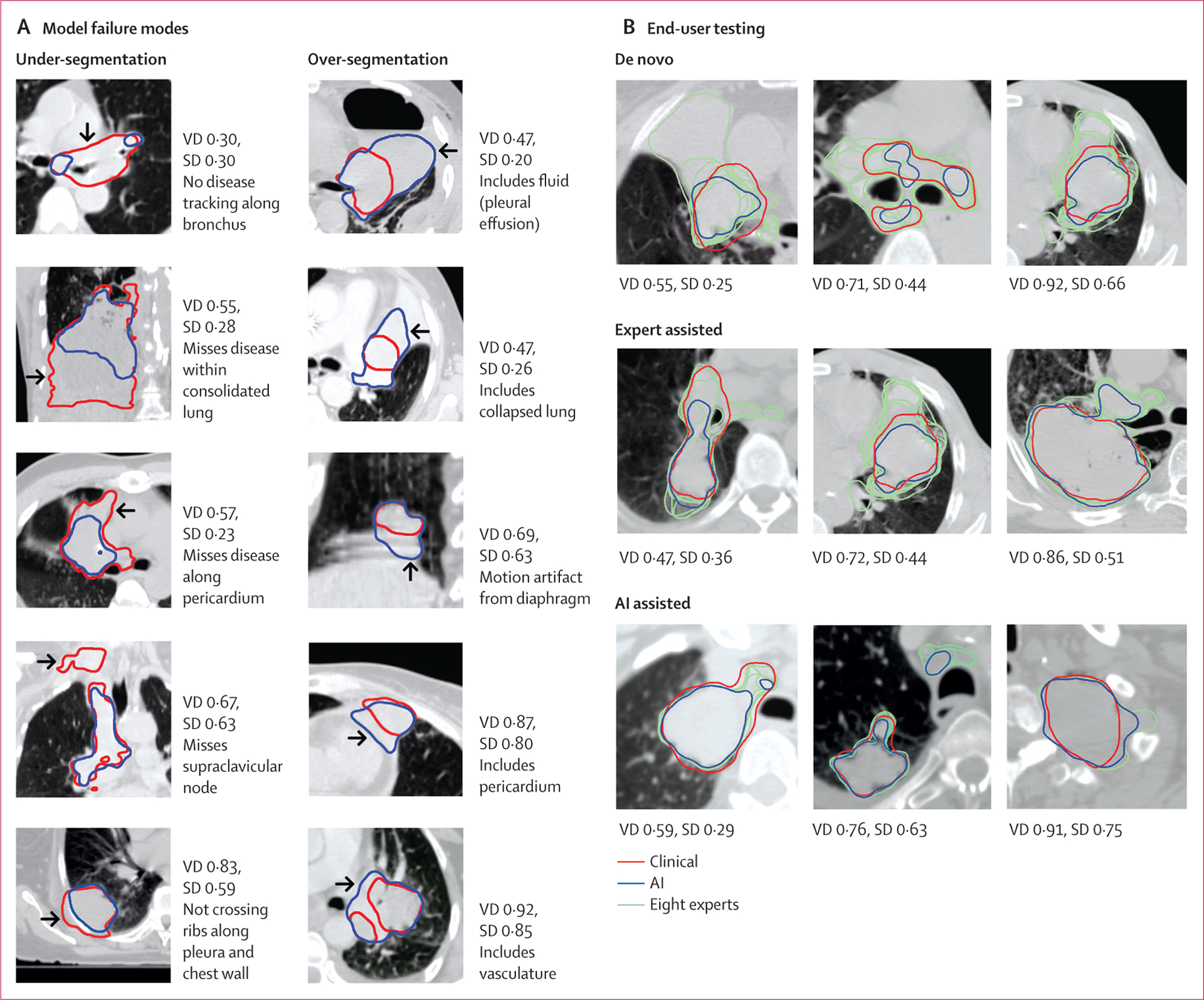
Model failure modes and end-user testing (A) Examples of model failure modes. Includes ten representative examples of model failures for both under-segmentation and over-segmentation scenarios (five cases each). Cases are ordered top to bottom in increasing model performance metrics. (B) Nine representative examples from the end-user testing. Depicted scores are calculated between clinical and AI segmentations. AI=artificial intelligence. SD=surface dice. VD=volumetric dice.

Finally, model failure modes were examined through expert review. These included missing thoracic nodal stations originally undersampled in the discovery data (eg, supraclavicular nodes; appendix p 67), over-segmentation into pericardium and collapsed lungs, and susceptibility to motion artifacts around the diaphragm (figure 3A).

**Figure 4:**
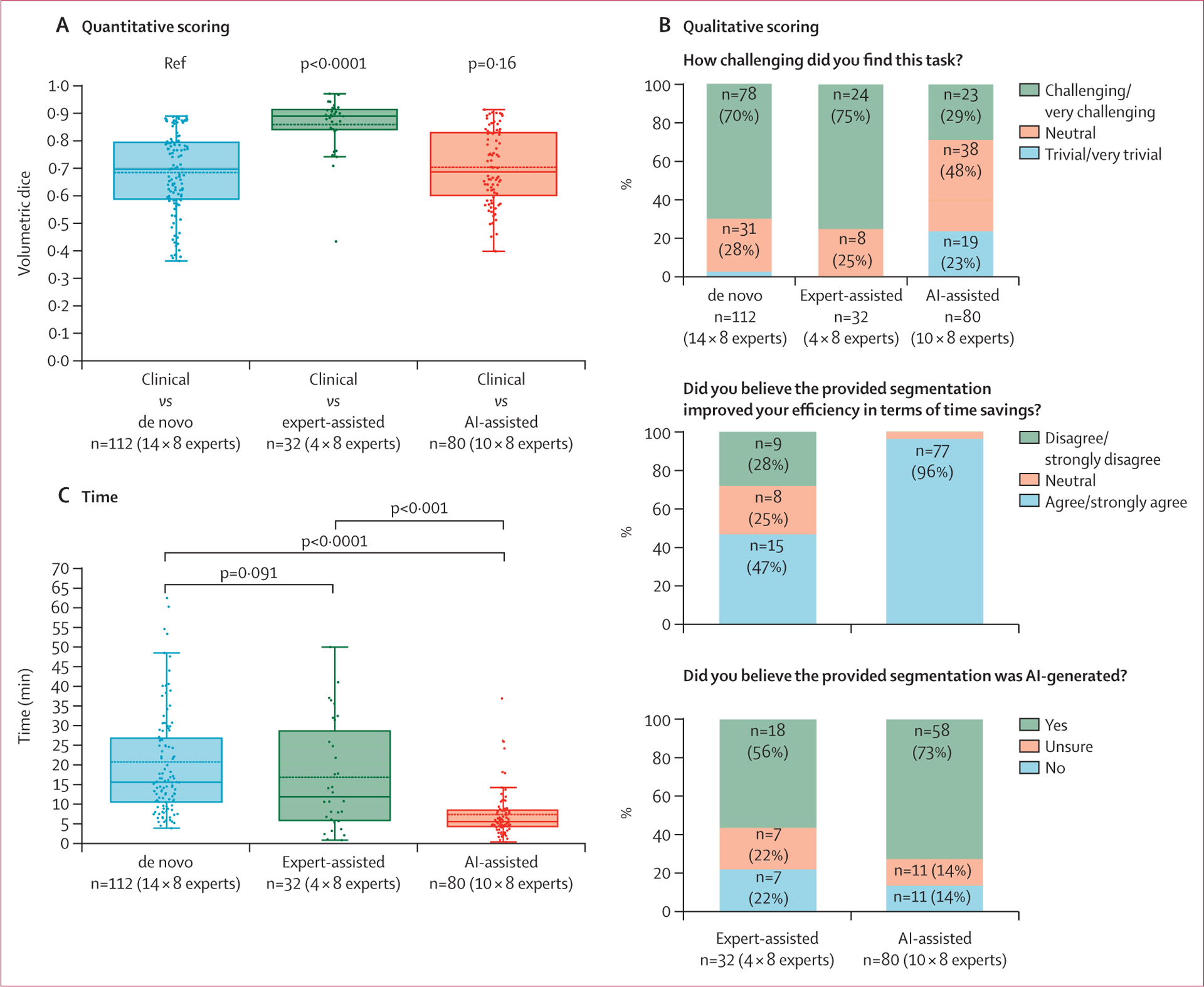
Results from the end-user testing (A) The volumetric dice score between clinical trial segmentations and each of de novo, expert-assisted (clinical segmentation provided), and AI-assisted (AI-generated segmentation provided) segmentations. (B) Answers to qualitative questions asked to experts during the end-user testing. (C) Time needed to complete the segmentation task. AI=artificial intelligence.

Eight experts were asked to perform the segmentation task de novo, or rate and edit a provided segmentation while masked to its source (figure 3B). Provided segmentations were either clinical (expert-assisted) or AI-generated (AI-assisted). Using clinical segmentations as reference, we found non-significant differences between de novo (VD 0**·**70 [IQR 0·59–0**·**80]; SD 0·43 [0·35–0.52]) and AI-assisted (VD 0·69 [0**·**60–0·83]; SD 0·38 [0**·**30–0·64]) segmentations (figure 4A; appendix p 54), with similar results across individual experts (appendix pp 55–56). When compared with de novo segmentation time (median 15·5 min), expert assistance led to a non-significant reduction of 24% (11·7 min; p=0·091), whereas AI assistance led to a significant reduction of 65% (5·4 min; p<0·0001; figure 4C). We found non-significant differences between de novo segmentations by residents and attendings (appendix p 57). When compared with the de novo IQR of interobserver variability, AI assistance led to a non-significant reduction of 53% for VD (p=0·092) and a significant reduction of 32% for SD (p=0·013; appendix pp 58–59).

**Figure 5:**
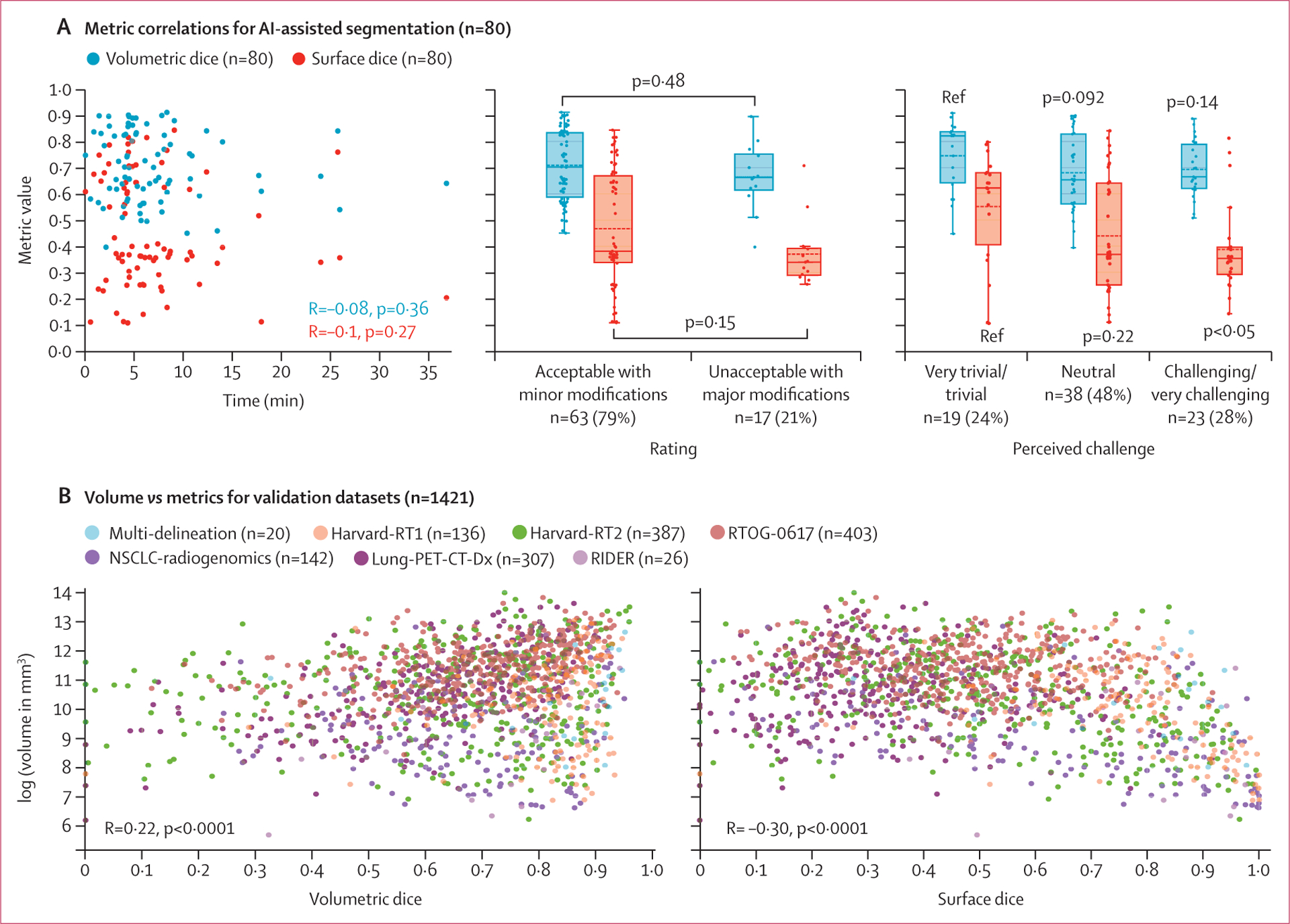
Analysis of segmentation metrics (A) The correlation of segmentation metrics with time needed to edit AI segmentations, qualitative rating provided by experts, as well as the perceived challenge. Data used for this analysis are from the end-user testing when an AI segmentation was provided to experts (n=80, ten cases × eight experts). (B) The correlation of segmentation metrics with tumour volume (displayed on log scale). We used all validation datasets for this analysis (n=1421). AI=artificial intelligence.

We also collected qualitative survey data. For 77 (96%) of 80 AI segmentations, experts agreed that the provided segmentations improved their efficiency (figure 4B). Experts identified 58 (73%) AI segmentations as being AI-generated (figure 4B). Finally, we found that VD and SD metrics did not correlate with the time required to edit AI segmentations, nor did they significantly stratify subgroups on the basis of expert rating and perceived difficulty. 63 (79%) AI segmentations were rated as “acceptable with minor modifications” (figure 5A). We also found significant correlations between metrics and tumour volume (VD, R=0·22, p<0·0001; and SD, R=–0·30, p<0·0001; figure 5B).

## Discussion

In this study, we developed a multifaceted strategy for the clinical validation of deep learning models for radiotherapy targeting, a crucial component of cancer therapy. Beyond establishing benchmarks, we performed multi-tiered validation on internal and external datasets including clinical trial and diagnostic radiology data. We also carried out dosimetric validation—the ultimate functional objective of segmentation in this clinical context—and measured the models’ stability and accuracy. Finally, we did end-user testing to measure clinical utility and physician acceptance.

Starting with benchmarks, our results underscore the model’s ability in identifying challenging cases with large interobserver variability. Although we showed no difference in performance between residents and attending physicians, further work is needed to understand the effect of experience on human–AI interaction and the potential of such models to augment physician training.

Our tiered validation process started with single-expert internal test data (Harvard-RT1) that most resembled the training data. We reported a significant improvement over previously published results with 45 different models tested on the same data.^16^ The decrease in performance at the multi-expert internal test data (Harvard-RT2) in the context of its relative stability on subsequent increasingly external datasets suggest that segmentation variability might be a function of treating physician preference and experience. Results from 4D CT data imply the models’ relevance towards modern imaging practices. Results from the diagnostic datasets highlight known differences in tumour definition between radiologists (anatomical knowledge) and radiation oncologists (therapeutic goals; appendix pp 42, 60). These findings stimulate further discussion around the off-label use of AI, where applications developed within one speciality are deployed in another, while emphasising the importance of radiologist input in radiotherapy planning.^28^

Our functional validation and end-user testing underscore the importance of assessing segmentations beyond common geometric measures. Similar to previous studies,^29^ our dosimetric analysis showed no correlation between geometric and dosimetric measures. This relationship is likely to be confounded by factors affecting dosimetric measures including dose distribution, radiotherapy treatment technique, beam arrangement, and other patient-specific considerations. Additionally, we also found that geometric measures might not accurately reflect time savings and other qualitative endpoints (figure 5A). Our results also highlight undesired correlations between common metrics and tumour volume (figure 5B; appendix p 13). As such, an unmet need exists for new metrics that combine qualitative physician assessment with geometric, dosimetric, and time-related measures to accurately reflect clinical utility and acceptability.^30^

Model failure modes might be automatically detected with warnings that model outputs might be compromised, thereby bringing much needed trust into automated systems.^31^ In terms of tumour localisation failures, our automated models failed in 6% of validation cases, in line with false-positive rates in similar lung cancer diagnostic settings.^32^ Although these failures require fallback onto assisted models, future iterations might be augmented through the automated extraction of rough anatomic tumour location from other sources such as clinical notes to ensure accurate model localisation. Finally, the exact effects of imaging contrast on model performance remain unclear, as our models significantly over-performed on contrast-enhanced images despite being trained primarily on non-contrast data (appendix p 65).

Several limitations should be noted. Both our in silico and end-user testing are limited by their retrospective nature. Many of our discovery data relied on a single human expert. Although this method enabled us to highlight the model’s ability to encapsulate the skills of a given expert and share it with other clinicians as decision support, our models might have acquired a natural bias. Our radiologist versus radiation oncologist comparisons relied on AI-generated segmentations, which might not be fully representative of radiotherapy segmentations. Our dosimetric analysis might not always have reflected clinical reality because such analysis does not allow for manually editing the intermediate volume between gross tumour volume and planning target volume, namely the clinical target volume. Results from Lung-PET-CT-Dx were calculated on tumour bounding boxes, and should therefore be interpreted accordingly. The design of our end-user tests did not allow for studying AI effects on intraobserver variability, nor did we incorporate PET imaging, commonly used in radiotherapy planning for patients with NSCLC. Finally, although masking experts to the source of provided segmentations improved the fairness of the assessment of the AI model, this design did not test human bias towards a clinical AI algorithm.

Future directions include improving segmentation performance—both in silico and clinically—through experimentation with various model types, architectures, ensemble approaches, pooling of multi-expert segmentations, validation on external benchmarks, and prospective testing. To enable separate reporting of segmentation results on lymph nodes, improved data curation strategies are needed to unambiguously isolate these from combined gross tumour volume segmentations.

Early and thorough testing of AI tools in clinical environments is crucial for successful translation into clinical practice. Our four-component validation strategy allows for uncovering downstream consequences of implementing AI models in the clinic, those that might otherwise go unnoticed in typical in silico validation. We encourage the broader adoption of similar validation strategies that help close the translational gap for clinical AI applications.

## Supplementary Material

1

## Data Availability

The following datasets are publicly available on the Cancer Imaging Archive website: Maastro, Multi-delineation, RTOG-0617, NSCLC-radiogenomics, Lung-PET-CT-Dx, RIDER, and Thorax phantom. RTOG-0617 is a limited access dataset. Harvard-RT1 and Harvard-RT2 are not publicly available. See appendix (pp 1–6) for individual URLs. For output data, including AI-generated segmentations and tabular result metrics, see https://github.com/AIM-Harvard/DL-RT-segmention.

## Research in context

### Evidence before this study

We searched PubMed using the following search terms: “(machine learning OR artificial intelligence OR deep learning) AND (radiation oncology OR radiotherapy)”. We focused on articles in English using medical imaging from Jan 1, 2014, to April 1, 2020. Our search identified 1383 articles. Within the same search, we also identified studies that did end-user validation by deploying and testing artificial intelligence (AI) tools clinically, whether retrospectively or prospectively. Although deep learning applications in medical imaging have shown great promise, most studies remain at the proof-of-concept stage, often validated in small cohorts and without external validation. Additionally, most are confined to in silico testing, with only a few investigating model performance in clinical settings. This insufficient evidence on real-world clinical utility ultimately translates to poor confidence in pursuing prospective clinical trials to clinically assess models.

### Added value of this study

In this study, we developed a multifaceted strategy for the clinical validation of deep learning models, aimed at closing the translational gap that falls in between early in silico validation and larger-scale prospective clinical trials. The strategy comprises four components: (1) developing clinical benchmarks to understand the current standard of care; (2) primary validation in large external cohorts to understand the models’ generalisability profile; (3) functional validation to study the models’ effect on downstream clinical endpoints; and (4) end-user testing in simulated clinical settings to assess models in their deployed state. Although in silico validation of AI models might provide evidence of clinical utility, measuring performance within the deployment context is often needed. Our work underscores the value of performing both these validation modes and provides insight into instances where disagreements between them occur, shedding light on potential issues such as the choice of evaluation metrics.

### Implications of all the available evidence

Our findings highlight the importance of broader adoption of similar validation strategies that help close the translational gap for clinical AI applications. Such strategies might provide the high levels of confidence needed to pursue clinical AI trials in medicine, uncover model weaknesses, and quantify the time and effort needed to bring AI outputs to clinically acceptable levels. Our results also underscore the importance of generating data on human factors given our incomplete understanding of this area, together with studying physicians’ interactions with AI outputs and their overall satisfaction.
