# Supplementary material for: Clinical validation of deep learning algorithms for radiotherapy targeting of non-small-cell lung cancer: an observational study
Source: Lancet Digit Health. Author manuscript; Available in PMC 2022 Sep 1. (PMC9435511; doi:10.1016/S2589-7500(22)00129-7)
Supplement: 1 [file NIHMS1832205-supplement-1.pdf]

THE LANCET
Digital Health

Supplementary appendix

This appendix formed part of the original submission and has been peer reviewed. We post it as supplied by the authors.

Supplement to: Hosny A, Bitterman DS, Guthier CV, et al. Clinical validation of deep learning algorithms for radiotherapy targeting of non-small-cell lung cancer: an observational study. *Lancet Digit Health* 2022; **4:** e657–666.

Supplementary Methods

Datasets

Maastro

Maastro Clinic, Maastricht, The Netherlands
Stages I-IIIB; 290 male, 132 female; mean age 68
Between 2004 and 2010

Patient population: 422 consecutive patients were included (132 women and 290 men), with inoperable, histologic or cytologic confirmed NSCLC, UICC stages I-IIIb, treated with radical radiotherapy alone (196) or with chemo-radiation (226). Mean age was 68 years (min: 33, max: 91 years).

Data collection: All patients received an FDG PET-CT scan for radiotherapy treatment planning, in radiotherapy position on a dedicated PET-CT simulator with both arms above the head. For the FDG PET-CT scans a Siemens Biograph (SOMATOM Sensation-16 with an ECAT ACCEL PET scanner) was used. An intravenous injection of (weight * 4 + 20) MBq FDG (Tyco HealthCare, Amsterdam, The Netherlands) was followed by 10 ml physiologic saline. After a 45-min uptake period, during which the patient was encouraged to rest, PET and CT images were acquired. A spiral CT (3 mm slice thickness) with or without intravenous contrast was performed covering the complete thoracic region. Radiotherapy planning was performed on a XiO (Computerized Medical Systems, St Louis, Missouri) treatment planning system, based on a convolution algorithm using inhomogeneity corrections.

Segmentations: Delineation based on fused PET-CT images was performed by the radiation oncologist by using a standard clinical delineation protocol. The protocol included fixed window level settings of both CT (lung W1700; L–300, mediastinum W600; L40) and PET scan (W30000; L15000) to be used for delineation. For all patients, a gross tumor volume (GTV) was defined based on FDG PET-CT data. All research was carried out in accordance with Dutch law. The Institutional Review Board of the Maastricht University Medical Center (MUMC+) waved the review due to the retrospective nature of this study.

See clinical information (Supplementary Table 8). Data were downloaded from The Cancer Imaging Archive (TCIA): https://wiki.cancerimagingarchive.net/display/Public/NSCLC-Radiomics

Harvard-RT1

Dana-Farber Cancer Institute, Brigham and Women's Hospital, Boston, Massachusetts, US
Stages IA-IV; 263 male, 236 female, 2 unspecified; median age 73
Between 2001 and 2015

Data collection: The data was collected under an IRB-approved retrospective protocol with a waiver of consent (Dana-Farber/Harvard Cancer Center protocol 11-286). The data set

consisted of fully anonymized computerized tomography (CT) scans (512x512 pixel, 16-bit grayscale image slices) which were clinically utilized for radiation treatment planning in 501 patients diagnosed with non-small cell lung cancer (NSCLC) from 2001 to 2015. All CT scans were taken using GE Lightspeed QX/i (4%), Lightspeed RT (36%) and Lightspeed RT16 (60%) equipment with slice thicknesses 2.5mm (96%), 3.75(2%) and 5mm (2%). The primary tumor segmentations were re-drawn by a single radiation oncologist with expertise in lung cancer treatment (R.H.M), and incorporated both analysis of CT image and the following available clinical data: 1) other image modalities such as 18F-fluorodeoxyglucose positron emission tomography (PET), and 2) clinical reports including staging procedures and pathology.

Data split: The curated data set was randomly divided into three disjoint subsets: a training set comprising 269 patients, a tuning set comprising 96 patients, and a holdout test set comprising 136 patients. The test set is identical to that used in a previously published tumor segmentation study[1]**.** 21 patients were randomly selected from the test set to develop the intraobserver benchmark.

See clinical information (Supplementary Table 9).

Multi-delineation[2]

Maastro Clinic, Maastricht, The Netherlands
Stages IA–IIIB; 12 male, 8 female; median age 67

22 patients with histologically proven NSCLC, stages Ib–IIIb, were included. All patients had undergone a diagnostic whole body PET/CT scan. The CT scan was a spiral CT scan of the whole thorax with intravenous contrast. The PET images were acquired in 5-min bed positions. For all patients, the primary tumour was segmented manually on CT/PET scans by five independent radiation oncologists: three radiation oncologists specializing in thoracic oncology, and two residents. GTV manual segmentations were based on fused PET/CT images using a standard clinical segmentation protocol. Briefly, the protocol included fixed window level settings of both CT (lung W 1,700; L –300, mediastinum W 600; L 40) and PET scan (W 30,000; L 15,000) to be used for segmentation . All observers were blinded to each other's segmentations. The primary gross tumor volume (GTV) was defined for each patient based on combined CT and PET information. Segmentations were performed on a treatment planning system (XiO; Computer Medical System, Inc., St. Louis, MO). The Institutional Review Board of the Maastricht University Medical Center (MUMC+) waved the review due to the retrospective nature of this study.

See data exclusion diagram (Supplementary Figure 43) and clinical information (Supplementary Table 10). Data were downloaded from The Cancer Imaging Archive (TCIA): https://wiki.cancerimagingarchive.net/display/Public/NSCLC-Radiomics-Interobserver1

Harvard RT-2

Dana-Farber Cancer Institute, Brigham and Women's Hospital, Boston, Massachusetts, US
Stages IA-IV; 165 male, 222 female; median age 69

Data collection: The data was collected under an IRB-approved retrospective protocol with a waiver of consent (Dana-Farber/Harvard Cancer Center protocol 11-286). The data set consisted of fully anonymized computerized tomography (CT) scans (512x512 pixel, 16-bit grayscale image slices) which were clinically utilized for radiation treatment planning in 387 patients diagnosed with non-small cell lung cancer (NSCLC) from 2011 to 2017. The primary tumor segmentations were drawn by multiple radiation oncologists with expertise in lung cancer treatment.

Data split: This dataset is split into two groups based on available imaging data. The first group (n=186) with single timeframe 3D CT data and corresponding GTV annotations, and the second group (n=201) with multi timeframe 4D CT data and iGTV annotations.

See clinical information (Supplementary Table 11).

RTOG-0617[3]

Multiple US and Canadian institutions
Stages IIIA-IIIB; 223 male, 155 female, 25 unspecified; median age 64
Between 2007 and 2011

Data was collected as part of the randomised phase 3 clinical trial NCT005339493[4], "High-Dose or Standard-Dose Radiation Therapy and Chemotherapy With or Without Cetuximab in Treating Patients With Newly Diagnosed Stage III Non-Small Cell Lung Cancer That Cannot Be Removed by Surgery." Patients recruited for this trial were aged 18 years and older with stage IIIA/IIIB NSCLC from 185 institutions in the USA and Canada. The trial accrued 544 patients between Nov 2007 and Nov 2011, and patients were assigned to one of four arms: 166 to receive standard-dose chemoradiotherapy, 121 to high-dose chemoradiotherapy, 147 to standard-dose chemoradiotherapy and cetuximab, and 110 to high-dose chemoradiotherapy and cetuximab. CT imaging of the thorax was required within 6 weeks of registration. The institutional review board (IRB) of each participating institution approved the study protocol. Patients were required to read and sign an IRB approved informed consent document.

See data exclusion diagram (Supplementary Figure 44) and clinical information (Supplementary Table 12). Data were downloaded from The Cancer Imaging Archive (TCIA): https://wiki.cancerimagingarchive.net/pages/viewpage.action?pageId=33948334

NSCLC-radiogenomics[5]

Stanford University School of Medicine and Palo Alto Veterans Affairs Healthcare System, USA
Pathological stages T1-T3, N0-N2, M0-M1; 124 male, 38 female; mean age 68

Patient population: With approval of respective Institutional Review Boards (IRB), the authors collected the "R01" cohort which comprised n=162 NSCLC patients (n=38 females, n=124 males, age at scan: mean 68, min:42, max:86) from Stanford University School of Medicine (n=69) and Palo Alto Veterans Affairs Healthcare System (n=93). Recruitment took place between April 7th, 2008 and September 15th, 2012. Patients signed written consent forms according to the guidelines of the institutions' IRBs. Patients were early stage NSCLC patients referred for surgical treatment with preoperative CT and PET/CT performed prior to surgical procedures.

Images: Given the retrospective nature of the data collected, different subjects were scanned using different scanners, scanning protocols, and parameters. Slice thickness of 0.625–3 mm (median: 1.5 mm) and an X-ray tube current of 124–699 mA (mean 220 mA) at 80–140 kVp (mean 120 kVp). Scans were acquired with subjects in supine position with arms at sides, from the apex of the lung to the adrenal gland within a single breath-hold.

Segmentations: Segmentations for 144 subjects were obtained from CT images using an unpublished automatic segmentation algorithm. These were then viewed by a thoracic radiologist with more than 5 years of experience and edited as necessary using ePAD. Final segmentations were reviewed by an additional thoracic radiologist. Disagreements in tumor boundaries were discussed and edited as appropriate.

See data exclusion diagram (Supplementary Figure 45) and clinical information (Supplementary Table 13). Data were downloaded from The Cancer Imaging Archive (TCIA): https://wiki.cancerimagingarchive.net/display/Public/NSCLC+Radiogenomics#6a3175f88bf2483c874777cadd372e8f

Lung-PET-CT-Dx[6]

Second Affiliated Hospital of Harbin Medical University, Harbin, Heilongjiang Province, China
Clinical stages T1-T4, N0-N3, M0-M13; 163 male, 144 female; mean age 61

Patient population: The images were retrospectively acquired from 355 lung cancer patients who underwent standard-of-care lung biopsy and PET/CT. Patient histologies included Adenocarcinoma, Small Cell Carcinoma, Large Cell Carcinoma, and Squamous Cell Carcinoma. Small Cell Carcinoma patients were excluded from this study. All research was carried out in accordance with Chinese law. The Institutional Review Board waived the review due to the retrospective nature of this study.

Images: Before the examination, the patient underwent fasting for at least 6 hours, and the blood glucose of each patient was less than 11 mmol/L. Whole-body emission scans were acquired 60 minutes after the intravenous injection of 18F-FDG (4.44MBq/kg, 0.12mCi/kg), with patients in the supine position in the PET scanner. FDG doses and uptake times were 168.72-468.79MBq (295.8±64.8MBq) and 27-171min (70.4±24.9 minutes), respectively. 18F-FDG with a radiochemical purity of 95% was provided. Patients were allowed to breathe normally during PET and CT acquisitions. Attenuation correction of PET images was performed

using CT data with the hybrid segmentation method. Attenuation corrections were performed using a CT protocol (180mAs,120kV,1.0pitch). Each study comprised one CT volume, one PET volume and fused PET and CT images: the CT resolution was 512 × 512 pixels at 1mm × 1mm, the PET resolution was 200 × 200 pixels at 4.07mm × 4.07mm, with a slice thickness and an interslice distance of 1mm. Both volumes were reconstructed with the same number of slices. Three-dimensional (3D) emission and transmission scanning were acquired from the base of the skull to mid femur. The PET images were reconstructed via the TrueX TOF method with a slice thickness of 1mm. The images were analyzed on the mediastinum (window width, 350 HU; level, 40 HU) and lung (window width, 1,400 HU; level, –700 HU) settings. The reconstructions were made in 2mm-slice-thick and lung settings. The CT slice interval varies from 0.625 mm to 5 mm. Scanning mode includes plain, contrast and 3D reconstruction.

Segmentations: The location of each tumor was annotated using slice-based bounding rectangles by five academic thoracic radiologists with expertise in lung cancer. Two of the radiologists had more than 15 years of experience and the others had more than 5 years of experience. After one of the radiologists labeled each subject the other four radiologists performed a verification, resulting in all five radiologists reviewing each annotation file in the dataset.

See data exclusion diagram (Supplementary Figure 46) and clinical information (Supplementary Table 14). Data were downloaded from The Cancer Imaging Archive (TCIA): https://wiki.cancerimagingarchive.net/pages/viewpage.action?pageId=70224216

RIDER[7]

Memorial Sloan-Kettering Cancer Center, New York, USA
Primary tumor >=1cm; 16 men, 16 women; mean age 62

Cohort: 32 patients (mean age 62.1; min:29, max:82) with pathologically confirmed NSCLC having measurable primary pulmonary tumors of 1 cm or larger. Patients were recruited between January 2007 and September 2007 at Memorial Sloan-Kettering Cancer Center. 16 patients were men (mean age 61.8; min:29, max:79) and 16 were women (mean age 62.4; min:45, max:82). The Institutional Review Board of the Memorial Sloan-Kettering Cancer Center waved the review due to the retrospective nature of this study.

Images: Each patient underwent two thoracic CT scans within 15 minutes of each other, acquired with the same CT scanner and using the same imaging protocol. CT scans were obtained with a 16-detector row LightSpeed 16 (GE Healthcare, Milwaukee, Wis) or a 64-detector row VCT (GE Healthcare) scanner. Thoracic images were acquired without intravenous contrast during a breath hold. Images were reconstructed without overlap by applying the lung convolution kernel. All images had an in-plane resolution of 0.576×0.576 mm/pixel and a slice thickness of 1.25 mm. Further patient and imaging details are described by Zhao et al[7]. The primary tumors were segmented, in both, test and retest scans, using a CT

single click ensemble segmentation algorithm. Correctness and consistency of these automated segmentations were visually inspected by two thoracic radiologists.

See data exclusion diagram (Supplementary Figure 47). Data were downloaded from The Cancer Imaging Archive (TCIA):
https://wiki.cancerimagingarchive.net/display/Public/RIDER+Lung+CT

Thorax phantom[8,9]

Columbia University Medical Center, New York, USA

The FDA anthropomorphic thorax phantom[10] with 12 phantom lesions of different sizes (10 and 20 mm in effective diameter), shapes (spherical, elliptical, lobulated, and spiculated), and densities (−630,−10, and +100 HU) was scanned at Columbia University Medical Center on a 64-detector row scanner (LightSpeed VCT, GE Healthcare, Milwaukee, WI). The CT scanning parameters were 120 kVp, 100 mAs, 64x0.625 collimation, and pitch of 1.375. The images were reconstructed with the lung kernel using 1.25 mm slice thickness.

See imaging information (Supplementary Table 15). Data were downloaded from The Cancer Imaging Archive (TCIA): https://wiki.cancerimagingarchive.net/display/Public/Lung+Phantom

Data preprocessing

Data preprocessing involved resampling all data to a common voxel spacing of 1*1*3 mm^3 (Supplementary Figure 39). This was achieved using linear and nearest neighbor interpolations for CT images and segmentations, respectively. CT Hounsfield units were normalized by clipping to 0.5 and 99.5 percentiles. Use of intravenous contrast in images was detected using a published algorithm[11]. During model training, data augmentation we performed using batchgenerators[12], and closely followed those published in nnU-Net[13]. These included scaling (drawn from 0.7 to 1.4 multiplier range), rotating (drawn from -30 to 30 degree range), mirroring (along all 3 axes), addition of gaussian noise (variance drawn from 0 to 0.1 range) and blur (kernel width drawn from 0.5 to 1.5 range) as well as brightness (multiplier drawn from 0.7 to 1.3 range) and contrast adjustments (multiplier drawn from 0.65 to 1.5 range).

Model development

Our assisted and automated pipelines consist of four 3D U-Net models - closely following the original implementation[14,15] - for the localization and segmentation of lungs, primary tumor, as well as involved thoracic lymph nodes. The assisted pipeline requires a user-placed seed point within the tumor volume, while the automated pipeline is fully autonomous. For pipeline schematics, see Supplementary Figure 17. Each model comprised 2 blocks per level along both the encoder and decoder. Each block contained a convolutional layer with instance normalization[16] and leaky ReLU activation[17]. Strided and transposed convolutions were used to downsample and upsample the images respectively. Number of feature maps started at 32 and was doubled and halved at every level along the encoder and decoder respectively. For model

specifications, see Supplementary Table 6. For training, we used the stochastic gradient descent (SGD) optimizer with Nesterov momentum (μ = 0.99) and an initial learning rate of .01 (decay using polynomial policy[18]) for a maximum of 1000 epochs. The loss function used was dice coefficient combined with cross entropy[19]. Pytorch[20] was used for model development, and nnU-Net[13] for hyper-parameter tuning. Multiple segmentation metrics were used for model validation (Supplementary Table 7).

Functional validation

Dosimetric analysis

While the models described herein predict the gross tumor volume (GTV), it is the planning target volume (PTV) that accounts for uncertainty and is ultimately used for dose calculation in RT planning. AI PTVs were generated from AI GTV as follows. First, a uniform expansion of 5mm was applied to the GTV to generate the clinical target volume (CTV), which is routinely used in clinical practice to account for microscopic tumor spread. This represents the lower bound of the 5mm to 10mm range specified in RTOG-0617 clinical trial protocol[3]. The CTV was further uniformly expanded by the mean margin between CTV and PTV segmentation used for each patient in the trial independently. Statistics of this margin were min=4.6mm, mean=8.1mm, max=12.1mm. The same treatment plans and radiation dose distributions used in the trial were then applied to this AI-based target volume and compared against the dosimetric data from the clinically treated radiation plan on trial. Dose volume histograms and other dose calculations were performed in 3Dslicer[21] using the SlicerRT extension[22].

Timeframe stability

Timeframe stability between 3D and 4D CT data was tested in Harvard-RT2 comprising n=186 single timeframe 3D CT with GTV annotations, against n=201 multi timeframe 4DCT with internal gross target volume (iGTV) annotations. The latter includes 10 reconstructed CT scans per patient encompassing the motion through the full respiration cycle. A GTV was predicted at each of the 10 timeframes and combined to produce an iGTV that compensates for the tumor's movements during respiration, and is the target that is most commonly utilized for clinical radiation planning.

Statistics

All statistical tests conducted were non-parametric, with a two-tailed $P < .05$ indicating significance. For two dependent groups, the Wilcoxon matched-pairs signed rank test was used. For two independent groups, the Mann-Whitney U rank test was used. For three or more independent groups, the Kruskal-Wallis H-test was used. For measuring correlation between two groups, the Spearman rank-order correlation coefficient was used.

End-user Testing

All participants were consented under protocol (DF/HCC 20-328).

Supplementary Tables

Dataset	n	Localization result			
		Successful localization	Failed localization		
			False positives	No predicted location	Total
Multi-delineation	20	20	0	0	0 (0%)
Harvard-RT1	136	133	3	0	3 (2%)
Harvard-RT2	387	347	40	0	40 (10%)
RTOG-0617	403	401	2	0	2 (0.5%)
NSCLC-radiogenomics	142	133	7	2	9 (6%)
Lung-PET-CT-Dx	307	280	25	2	27 (9%)
RIDER	26	20	6	0	6 (23%)
**Total**	**1421**	**1334**	**83**	**4**	**87 (6%)**
Thorax phantom	1 (12 lesions)	10	2	0	2(17%)

Supplementary Table 1: Table depicting the localization failures across all validation datasets.

ID	Position	Years in practice	Cases treated	Specialized in a disease site?	Years specialized in disease site?
R1 (R.H.M.)	Attending	9	1200	Yes/Thoracic	9
R2	Attending	2	270	No	0
R3	Attending	1	100	Yes/Thoracic	1
R4	Resident / 5th year				
R5	Resident / 4th year				
R6	Resident / 4th year				
R7	Resident / 4th year				
R8	Resident / 3rd year				

Supplementary Table 2: Table describing all eight participants in the end-user testing.

Survey questions		R1	R2	R3	R4	R5	R6	R7	R8
**Do you have prior experience with any AI in general?**	1=None<br>5=A lot	5	4	4	4	3	2	2	3
**Do you have prior experience with any AI in the clinic?**		2	1	2	2	1	3	1	1
**Do you conduct AI Research?**		5	3	5	5	3	3	3	1
**How much trust do you have in AI generally?**		3	3	3	3	4	3	4	3
**How often do you use any AI-Based automation or productivity tools in the clinic?**	1=Never<br>5=Always	1	1	2	3	1	4	1	1
**How often do you use automated contouring (non-AI or AI) in the clinic?**		3	1	2	3	1	2	2	2
**Do you believe AI may hurt contouring efficiency?**	1=Disagree<br>5=Agree	1	3	4	3	3	1	1	1
**Do you believe AI may benefit contouring efficiency?**		5	4	5	5	3	5	3	4
**Do you believe AI may hurt contouring quality?**		1	3	4	5	2	3	2	2
**Do you believe AI may benefit contouring quality?**		5	4	4	4	3	4	4	4
**Do you believe AI tools may hurt clinical practice and medicine generally in the long term?**		2	2	2	5	5	1	2	2
**Do you believe AI tools may benefit clinical practice and medicine generally in the long term?**		5	5	5	5	5	5	5	5

Supplementary Table 3: Results from a survey answered by participants in our end-user testing.

	patient_id	Volumetric dice (Clinical vs AI)	Quartile	Group	Task type	Segmentation provided (blinded)
1	0617-452389	0.3598742616		B	rate and adjust	AI
2	0617-342808	0.3857856988		A	*de novo*	
3	0617-559900	0.4714477505		B	rate and adjust	Clinical
4	0617-713112	0.5512047143	q1	A	*de novo*	
5	0617-635346	0.5563374087		B	rate and adjust	AI
6	0617-684904	0.5640645859		A	*de novo*	
7	0617-517268	0.5877014406		B	rate and adjust	AI
8	0617-466966	0.6410086813		A	*de novo*	
9	0617-475757	0.6658023209		B	rate and adjust	AI
10	0617-544413	0.6746766792		A	*de novo*	
11	0617-585616	0.7001307434		B	rate and adjust	Clinical
12	0617-309598	0.7134717441		A	*de novo*	
13	0617-709755	0.7138742639		B	rate and adjust	AI
14	0617-732035	0.7197506618	q2_3	A	*de novo*	
15	0617-588897	0.7210947574		B	rate and adjust	Clinical
16	0617-639972	0.7567666089		A	*de novo*	
17	0617-673893	0.7619517003		B	rate and adjust	AI
18	0617-624760	0.7645108426		A	*de novo*	
19	0617-619906	0.7769182559		B	rate and adjust	AI
20	0617-579641	0.7836360058		A	*de novo*	
21	0617-483282	0.809801991		B	rate and adjust	AI
22	0617-637689	0.8159860055		A	*de novo*	
23	0617-740237	0.8510139098		B	rate and adjust	AI
24	0617-670038	0.8552310143		A	*de novo*	
25	0617-671116	0.8622660288	q4	B	rate and adjust	Clinical
26	0617-498021	0.8653225652		A	*de novo*	
27	0617-657637	0.9093106938		B	rate and adjust	AI
28	0617-516857	0.9223921282		A	*de novo*	

Supplementary Table 4: Table illustrating the data utilized in the end-user testing. Data consisted of a random quartile-based 28 patient subset of the RTOG-0617 clinical trial dataset. This subset was further divided into two random quartile-based groups of 14 patients each. For

group A patients, readers were asked to perform the primary tumor and lymph node segmentation task de novo. For group B patients, readers were asked to rate and adjust a provided segmentation blinded to its source. For 10 patients, AI-generated segmentations were provided. For 4 patients, clinical segmentations (from RTOG-0617 clinical trial) were provided.

Dataset	n	Volume vs Volumetric dice (VD)		Volume vs Surface dice (SD)	
		Spearman R	*P*-value	Spearman R	*P*-value
**Multi-delineation**	20	0.52	.018	-0.2	0.39
**Harvard-RT1**	136	0.13	.13	-0.53	3.89e-11
**Harvard-RT2**	387	0.11	.037	-0.39	7.48e-16
**RTOG-0617**	403	0.44	7.89e-21	-0.11	.023
**NSCLC-radiogenomics**	142	-0.01	.86	-0.55	1.49e-12
**Lung-PET-CT-Dx**	307	0.54	3.87e-25	0.01	.81
**RIDER**	26	0.69	9.1e-05	-0.2	.31

Supplementary Table 5: Table depicting the amount of correlation between tumor volume and model performance across all validation datasets (n=1421). Data is shown for volumetric dice and surface dice.

Model #	Task	Input description	Input size and spacing (mm)	Discovery data used	Batch size	U-net levels	Kernel sizes
**1**	Full-scale segmentation of primary tumor and lymph nodes	Cropped ROI centered around user-provided see point(s)	160*160*64 1*1*3	n=787 (Maastro, Harvard-RT1)	2	5	Convolution: [1, 3, 3], [3, 3, 3], [3, 3, 3], [3, 3, 3], [3, 3, 3], [3, 3, 3] Pooling: [1, 2, 2], [2, 2, 2], [2, 2, 2], [2, 2, 2], [2, 2, 2]
**2**	Downsampled detection of lung	Raw CT image	108*108*96 (downsampled from 432*432*192) 4*4*6	n=325 (Harvard-RT1)	4	4	Convolution: [3, 3, 3], [3, 3, 3], [3, 3, 3], [3, 3, 3], [3, 3, 3] Pooling: [2, 2, 2], [2, 2, 2], [2, 2, 2], [2, 2, 2]
**3**	Downsampled detection of primary tumor and lymph node	Cropped ROI centered around lung segmentation (from model 2)	108*84*54 (downsampled from 324*252*108) 3*3*6	n=787 (Maastro, Harvard-RT1)	6	4	Convolution: [1, 3, 3], [3, 3, 3], [3, 3, 3], [3, 3, 3], [3, 3, 3] Pooling: [1, 2, 2], [2, 2, 2], [2, 2, 2], [2, 2, 2]
**4**	Full-scale segmentation of lymph nodes		144*144*64 1*1*3	n=365 (Harvard-RT1)	2	5	Convolution: [1, 3, 3], [3, 3, 3], [3, 3, 3], [3, 3, 3], [3, 3, 3], [3, 3, 3] Pooling: [1, 2, 2], [2, 2, 2], [2, 2, 2], [2, 2, 2], [2, 2, 2]

Supplementary Table 6: Table depicting the specifications of the four models used in this study

Metric	Definition
Precision	TP/(TP+FP)
Recall	TP/(TP+FN)
Jaccard	TP/(TP+FP+FN)
Segmentation score	$\exp\left[-\frac{E}{2V}\left(1+\left(\frac{V}{V_0}\right)^{1/3}\right)\right]$ See original implementation[1].
Volumetric dice (VD)	$\frac{2 \times TP}{2 \times TP + FP + FN}$
Surface dice (SD)	See original implementation[23]. Threshold used was 1.9mm, corresponding to the 75th percentile of the interobserver variability. See Supplementary Fig 1.
Volume	Number of voxels in object multiplied by the voxel size in mm^3.

Supplementary Table 7: Table depicting the definition of segmentation and other performance metrics used in this study. TP=true positive, FP=false positive, FN=false negative, E (Non-negative error volume) = V FN / TP + FP, V= tumor volume, V0 (scale parameter) = (4π/3) $(30mm)^3$

**Categories**		**Number of patients**	**%**
Gender	Male	290	68.7
	Female	132	31.3
T-stage	T1	93	22
	T2	156	37
	T3	53	12.6
	T4	117	27.7
	Tx	3	0.7
N-stage	N0	170	40.3
	N1	23	5.5
	N2	141	33.4
	N3	85	20.1
	Nx	3	0.7
TNM stage	I	93	22
	II	40	9.5
	IIIa	112	26.5
	IIIb	177	41.9
Treatment	Radiation only	196	46.5
	Chemo-radiation	226	53.5

Supplementary Table 8: Clinical information of the Maastro dataset[24]**.**

Categories		Number of patients	%
Gender	Female	263	52.5
	Male	236	47.1
	Unspecified	2	<1
Median age (min to max)		73 (39 to >=89*)	
Median tumor volume (min to max), cm3		16.40 (0.28 to 1103.74)	
Clinical stage**	IA	97	19.4
	IB	15	3
	IIA	12	2.4
	IIB	10	2
	IIIA	184	36.7
	IIIB	116	23.2
	IV	50	10
	Unspecified	17	3.4
Lobe (categories not exclusive)	No primary lung tumor	8	2
	Right upper lobe	184	37
	RIght middle lobe	40	7
	Right lower lobe	74	15
	Left upper lobe	123	25
	Left lower lobe	61	13
	Right endobronchial	4	1
	Left endobronchial	3	1
	Unspecified	20	4
IV contrast	Yes	168	33
	No	333	67
Histologic type	Adenocarcinoma	305	59
	Squamous cell carcinoma	97	20
	Non-small cell lung carcinoma	57	12
	Other	42	9

* 17 patients classified as 89 years or older during anonymization
** American Joint Committee on Cancer STaging, 7th edition

Supplementary Table 9: Clinical information of the Harvard-RT1 dataset. 461 patients from this dataset overlap with previously published data[1]**.**

PatientID	Sex	Age	Histology	Tumour Location	Clinical Stage			Overall Stage
					T	N	M	
interobs05	M	65	adenocarcinoma	right.middle.lobe	T2	N2	M0	IIIa
interobs06	M	82	squamous.cell.carcinoma	left.hilum	T1	N2	M0	IIIa
interobs08	M	66	adenocarcinoma	right.middle.lobe	T2	N2	M0	IIIa
interobs09*	F	47	non.small.cell.lung.cancer	right.upper.lobe	T1	N0	M0	Ia
interobs10	F	57	large.cell.carcinoma	left.upper.lobe	T1	N2	M0	IIIa
interobs11	M	74	adenocarcinoma	right.middle.lobe	T2	N2	M0	IIIa
interobs12	M	50	adenocarcinoma	right.lower.lobe	T2	N2	M0	IIIa
interobs13	F	68	adenocarcinoma	left.lower.lobe	T2	N0	M0	Ib
interobs14	M	77	undifferentiated.lung.carcinoma	right.lower.lobe	T2	N2	M0	IIIa
interobs15	M	70	squamous.cell.carcinoma	right.upper.lobe	T2	N2	M0	IIIa
interobs18	M	50	squamous.cell.carcinoma	left.lower.lobe	T2	N2	M0	IIIa
interobs19*	M	71	large.cell.carcinoma	left.hilum	T2	N2	M0	IIIa
interobs20	F	59	adenocarcinoma	left.upper.lobe	T2	N2	M0	IIIa
interobs21	M	70	large.cell.carcinoma	left.upper.lobe	T2	N2	M0	IIIa
interobs22	F	70	adenocarcinoma	right.upper.lobe	T2	N2	M0	IIIa
interobs27	F	49	non.small.cell.lung.cancer	right.middle.lobe	T4	N3	M0	IIIb
interobs28	F	75	squamous.cell.carcinoma	left.upper.lobe	T4	N2	M0	IIIb
interobs29	F	63	adenocarcinoma	left.lower.lobe	T2	N2	M0	IIIa
interobs31	M	73	squamous.cell.carcinoma	left.upper.lobe	T2	N2	M0	IIIa
interobs32	M	58	adenocarcinoma	left.upper.lobe	T2	N0	M0	Ib
interobs33	F	40	adenocarcinoma	right.upper.lobe	T2	N1	M0	IIb
interobs34	M	69	squamous.cell.carcinoma	right.upper.lobe	T2	N2	M0	IIIa

Supplementary Table 10: Clinical information of the Multi-delineation dataset. This data pertains to all 22 subjects in the original study, see[25].

Categories		Number of patients	%
Gender	Female	222	57.4
	Male	165	42.6
	Unspecified	0	0
Median age (min to max)		69 (32 to 92)	-
Clinical stage*	IA	46	11.9
	IB	5	1.3
	IIA	5	1.3
	IIB	3	0.8
	IIIA	26	6.7
	IIIB	8	2.1
	IV	22	5.7
	Unspecified	272	70.3
IV contrast	Yes	5	1.3
	No	16	4.1
	Unspecified	366	94.6
Histologic type	No pathology	32	8.3
	Adenocarcinoma	28	7.2
	Squamous cell carcinoma	17	4.4
	small cell lung cancer	12	3.1
	Large cell carcinoma	9	2.3
	Other	14	3.6
	Unspecified	275	71.1

* American Joint Committee on Cancer STaging, 7th edition

Supplementary Table 11: Clinical information of the Harvard-RT2 dataset.

Categories		Number of patients	%
Median age (IQR)		64.0 (57-70)	-
Gender	Female	155	38.5
	Male	223	55.3
	Unspecified	25	6.2
Trial arm	1 60 Gy, no cetuximab	124	30.8
	2 74 Gy, no cetuximab	95	23.6
	3 60 Gy + cetuximab	98	24.3
	4 74 Gy + cetuximab	86	21.3
Race	American Indian/Alaskan Native	2	0.5
	Asian	10	2.5
	Black or African American	32	7.9
	White	331	82.1
	Unspecified	28	6.9
Ethnicity	Hispanic or Latino	10	2.5
	Not Hispanic or Latino	355	88.1
	Unspecified	38	9.4
Histology	Squamous cell carcinoma	164	40.7
	Adenocarcinoma	153	38
	Large cell undifferentiated	9	2.2
	Non-small cell lung cancer NOS	52	12.9
	Unspecified	25	6.2
AJCC stage group	IIIA, or N2 with an undetectable primary	248	61.5
	IIIB, or N3 with an undetectable primary	130	32.3
	Unspecified	25	6.2

Supplementary Table 12: Clinical information of the RTOG-0617 dataset[3]**.**

**Categories**		**Number of patients**	**%**
Sex	Female	76	36.02
	Male	135	63.98
Ethnicity	African-American	6	2.84
	Asian	24	11.37
	Caucasian	123	58.29
	Hispanic/Latino	6	2.84
	Native Hawaiian/Pacific Islander	3	1.42
	Not Recorded	49	23.22
Histology	Adenocarcinoma	172	81.52
	Squamous cell carcinoma	35	16.59
	Not otherwise specified	4	1.9
Pathological T stage	T0	0	0
	Tis	6	2.84
	T1a	40	18.96
	T1b	31	14.69
	T1nos	0	0
	T2a	47	22.27
	T2b	10	4.74
	T2nos	0	0
	T3	21	9.95
	T4	7	3.32
	TX	0	0
	Not Collected	49	23.22
Pathological N stage	N0	129	61.14
	N1	15	7.11
	N2	18	8.53
	N3	0	0
	NX	0	0
	Not Collected	49	23.22
Pathological M stage	M0	157	74.41
	M1a	1	0.47
	M1b	4	1.9
	Not Collected	49	23.22

Histopathological Grade	G1 Well differentiated	32	15.17
	G2 Moderately differentiated	76	36.02
	G3 Poorly differentiated	33	15.64
	Other, Type I: Well to moderately differentiated	9	4.27
	Other, Type II: Moderately to poorly differentiated	12	5.69
	Not Collected	49	23.22
Peak kilovoltage (kVp)	100–120	See DICOM image headers for individual scans	-
X-ray Tube Current (mA)	28–749	See DICOM image headers for individual scans	-
Slice Thickness (mm)	0.625	12	5.69
	1	64	30.33
	1.5	114	54.03
	2	2	0.95
	2.5	15	7.11
	3	4	1.9

Supplementary Table 13: Clinical information of the NSCLC-radiogenomics dataset. Note: This data pertains to all 211 subjects in the original study, see[5]. Data used in this study is a subset of the "R01" cohort.

**Categories**		**Number of patients**	**%**
Mean age (min, max)		61.2 (28, 90)	-
Gender	Female	144	46.9
	Male	163	53.1
T-stage	1	1	0.3
	1a	7	2.3
	1b	26	8.5
	1c	119	38.8
	2	44	14.3
	2a	32	10.4
	2b	13	4.2
	3	45	14.7
	4	17	5.5
	Unspecified	3	1
N-stage	0	172	56
	1	72	23.5
	2	7	2.3
	3	56	18.2
M-stage	0	198	64.5
	1	48	15.6
	1a	28	9.1
	1b	21	6.8
	1c	10	3.3
	2	1	0.3
	3	1	0.3
Histopathological grading	G1	12	3.9
	G1-G2	8	2.6
	G2	27	8.8
	G2-G3	35	11.4
	G3	23	7.5
	Unspecified	202	65.8

Supplementary Table 14: Clinical information of the Lung-PET-CT-Dx dataset[26].

Scanned at site	kVp	mA	Slice thickness (mm)	Number of slices per study	Acquisition year
Columbia University Medical Center	120	195	1.25	237	2011

Supplementary Table 15: Imaging information for the thorax phantom[8].

Supplementary Figures

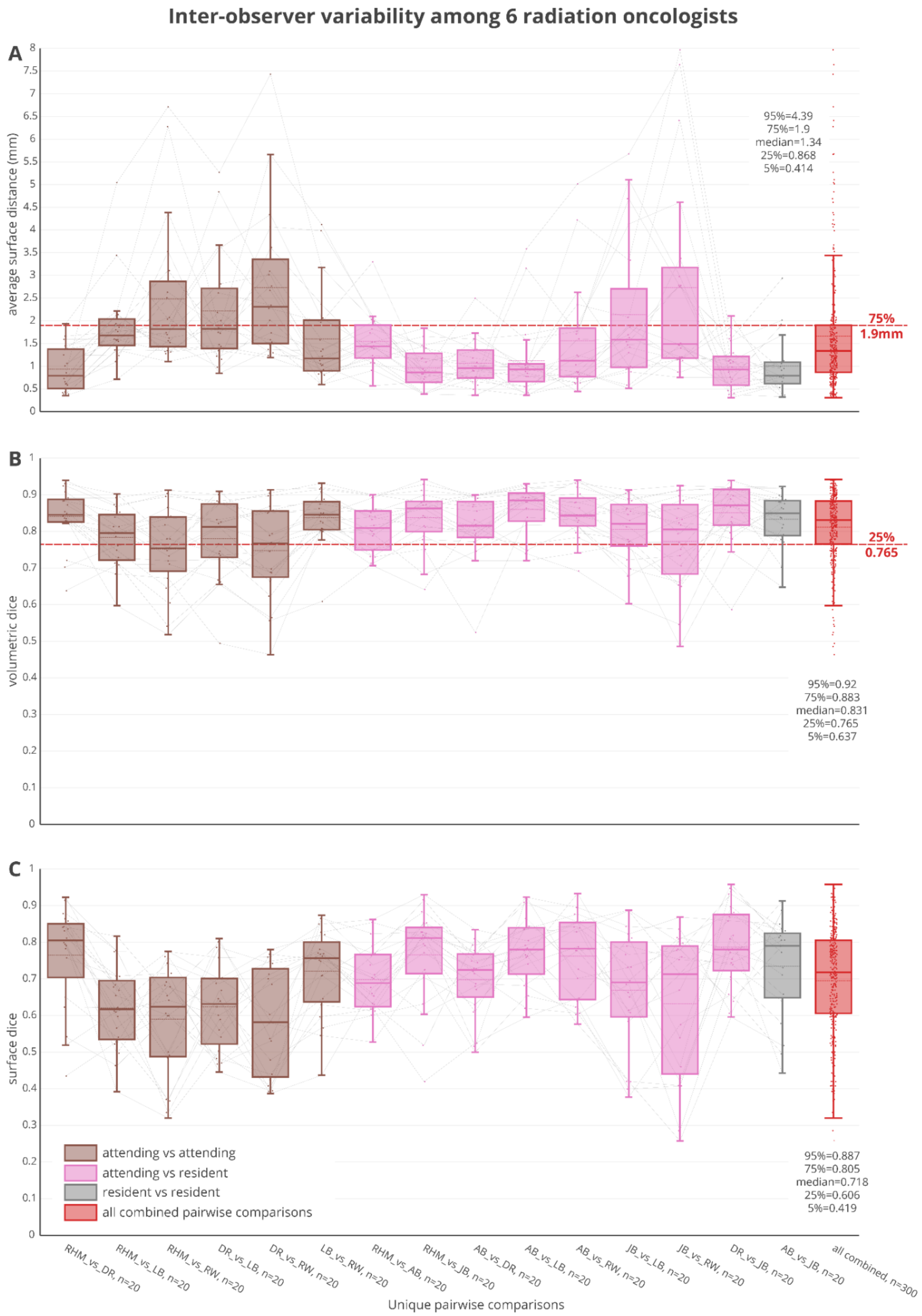

Supplementary Figure 1: Interobserver variability among 6 radiation oncologists: the expert from this study (R.H.M.) + five from a previously published and publicly available study[2]. The box plots represent all 15 unique pairwise comparisons between all 6 experts. Boxes are color coded based on expert level: attending and resident - see legend. The final red box represents all data points. Panel A shows the average distance between each two respective segmentations in mm. The 75th percentile of all data points (1.9mm) was used as a tolerance for calculating the surface dice (See Supplementary Table 7). Panel B shows the volumetric dice between each two respective segmentations. Panel C shows the surface dice between each two respective segmentations

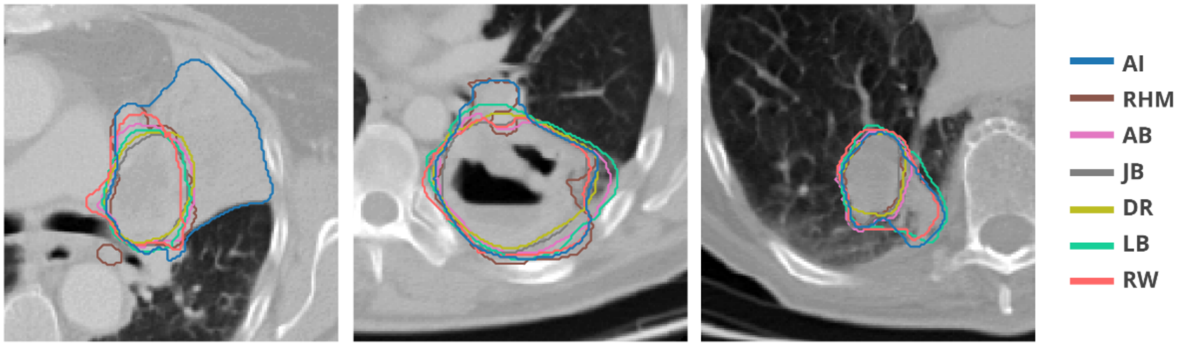

Supplementary Figure 2: Three examples showing AI segmentations (blue) and that of 6 human experts from the multi-delineation dataset.

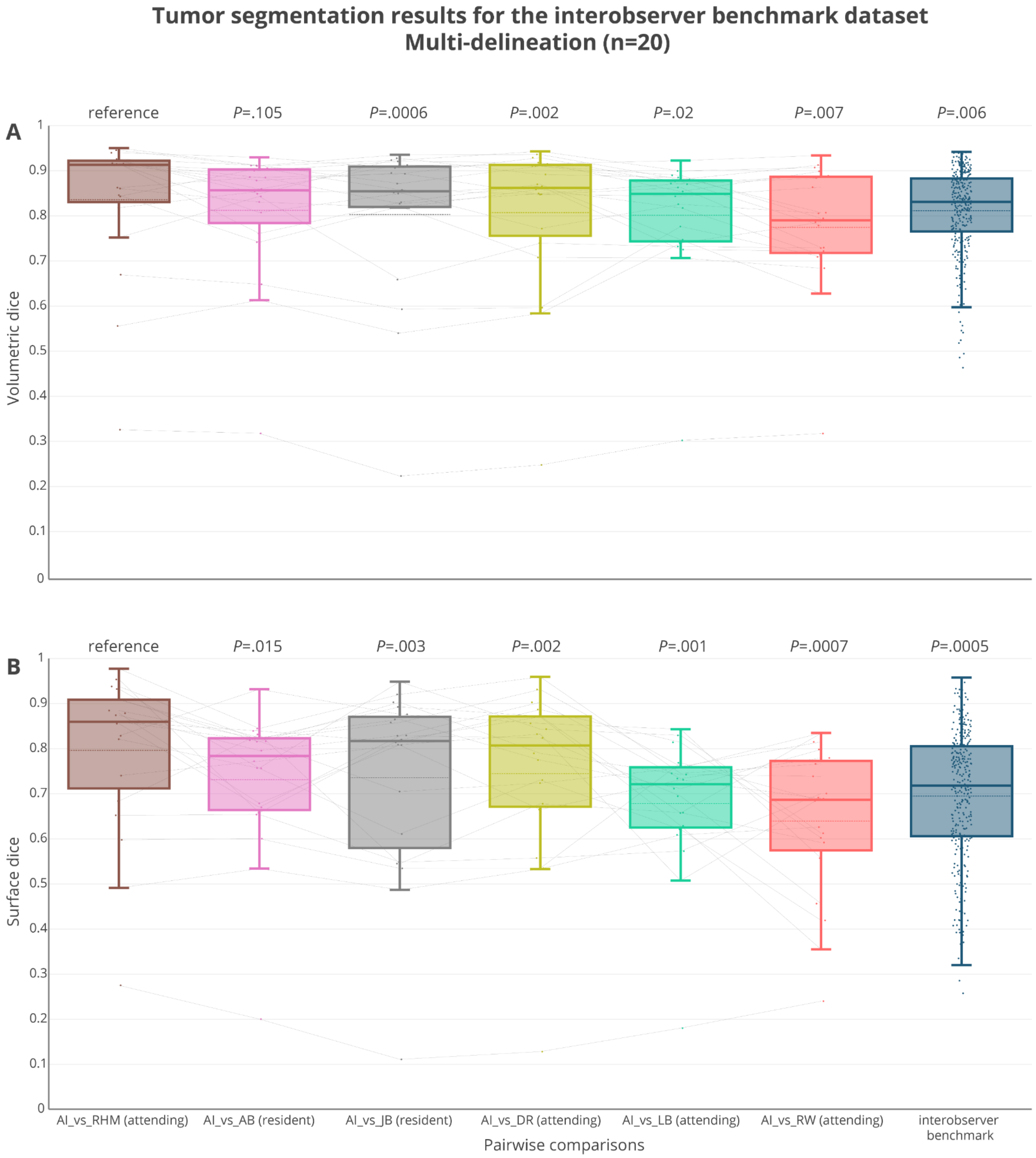

Supplementary Figure 3: Tumor segmentation results on the Multi-delineation dataset (n=20). The AI vs R.H.M. comparison (in brown) is used as reference. For volumetric dice (panel A), AI vs RHM was statistically better than 4 of 5 comparisons. For surface dice (panel B), AI vs RHM was statistically better than all other 5 comparisons. The Wilcoxon matched-pairs signed rank test was used, with a two-tailed $P < .05$ indicating significance. For both volumetric dice and surface dice, AI vs RHM was statistically better than the combined interobserver benchmark. The Mann-Whitney U rank test was used, with a two-tailed $P < .05$ indicating significance.

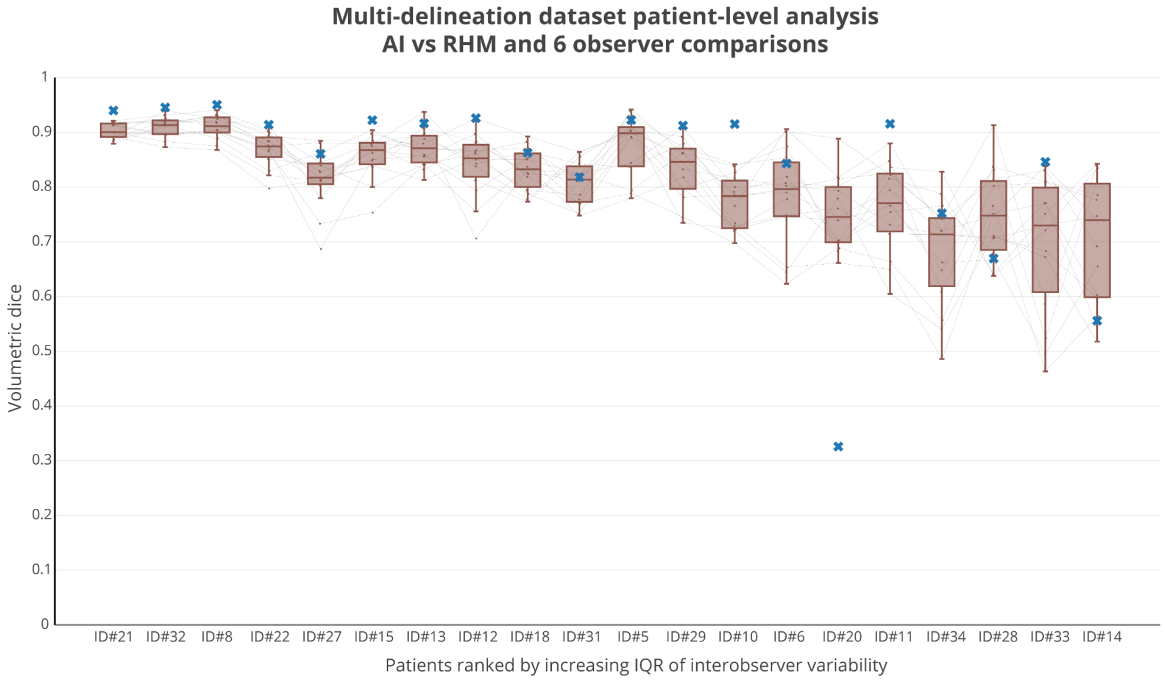

Supplementary Figure 4: Correlation between AI model performance and interobserver variability among experts. Brown box plots represent the interobserver variability for each case in the Multi-delineation dataset (n=20) ranked by increasing interquartile range (IQR). The blue crosses indicate AI model performance as measured against R.H.M. segmentations. AI vs R.H.M. was found to be inversely correlated with the interquartile range of variability among all 6 readers, Spearman R -0.74, *P*=0.00015. That is, the AI model is likely to underperform if experts disagree generally (as indicated by a larger IQR).

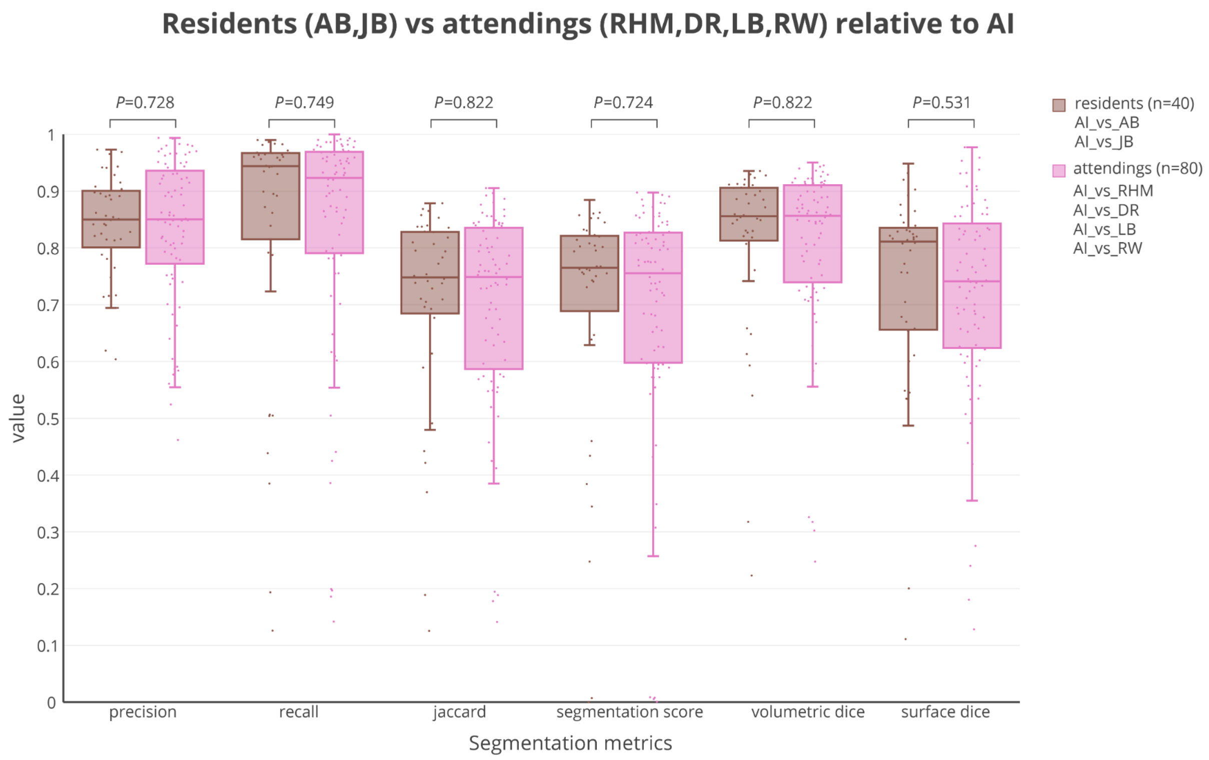

Supplementary Figure 5: Subgroup analysis comparing residents (n=40, 2 residents) and attendings (n=80, 4 attendings) and using AI output as reference. Non-significant differences were detected across all segmentation metrics. The Mann-Whitney U rank test was used, with a two-tailed *P* < .05 indicating significance.

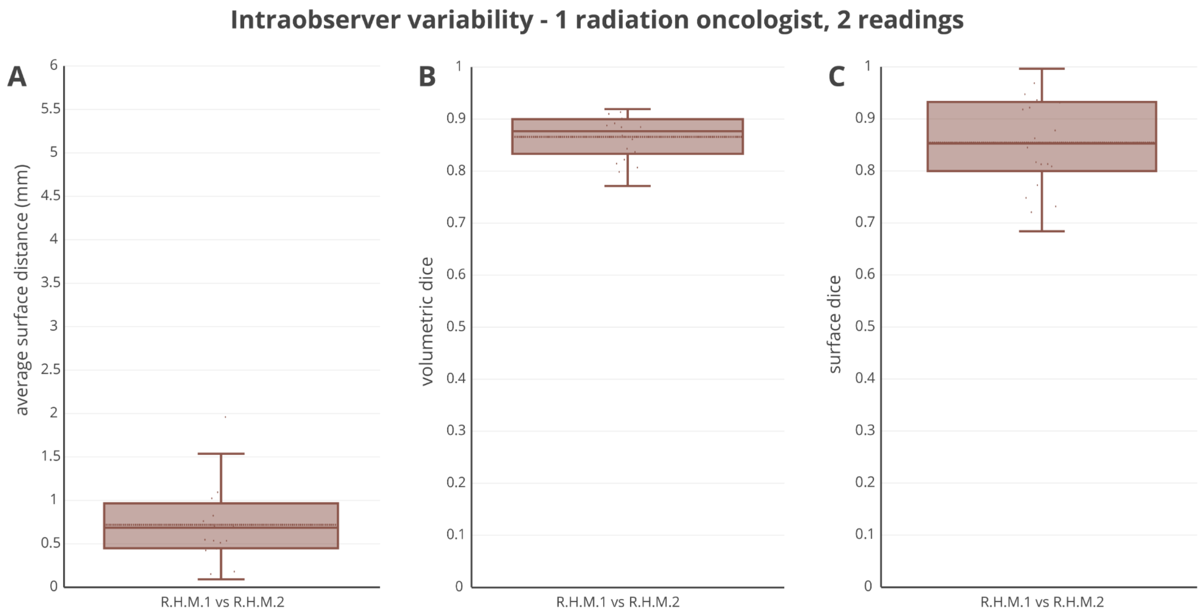

Supplementary Figure 6: Intraobserver variability among 2 readings by the same expert radiation oncologist, R.H.M. Panels A, B, and C show the average distance (mm), volumetric dice, and surface distance between the two segmentations respectively.

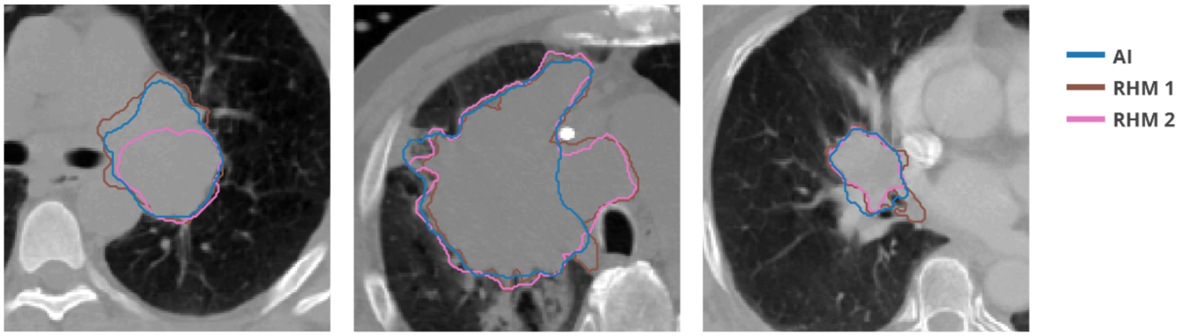

Supplementary Figure 7: Three examples showing AI segmentations (blue) and two segmentations by the same expert (R.H.M.) performed 3 months apart. Data is from the intraobserver benchmark (Harvard-RT1).

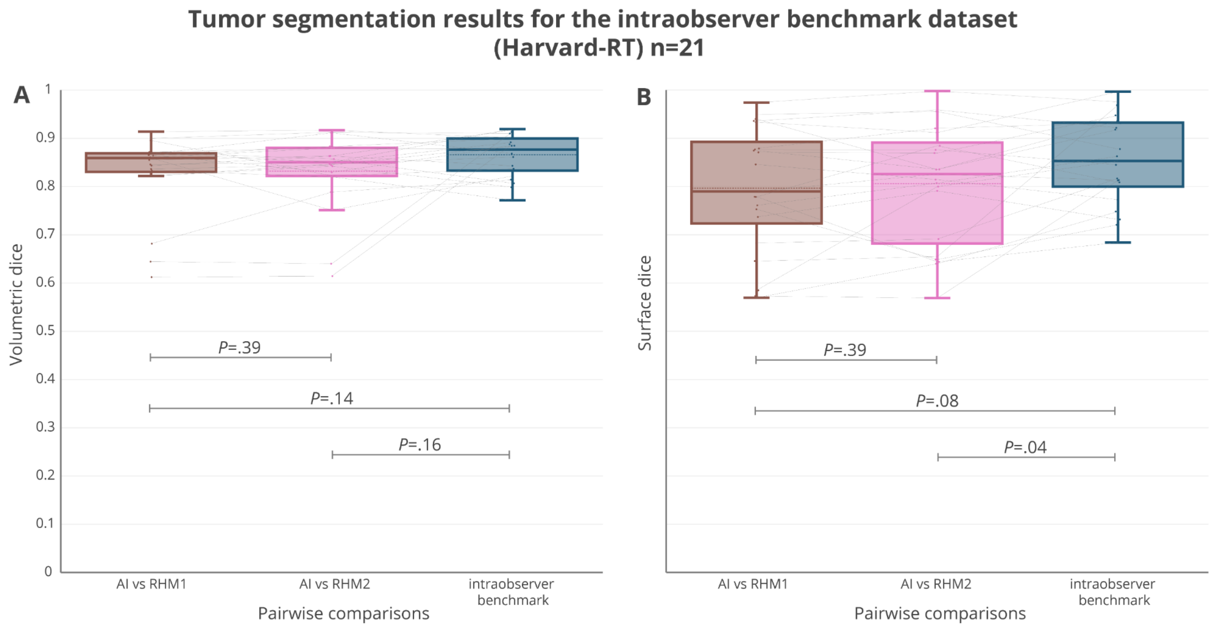

Supplementary Figure 8: Tumor segmentation results on the intraobserver benchmark (Harvardt-RT1, n=21). Non-significant differences were found when comparing AI to two segmentations performed by the same expert, three months apart. For volumetric dice (panel A), both AI vs RHM1 and AI vs RHM2 were found to be statistically similar to the benchmark (RHM1 vs RHM2). For surface dice (panel B), AI vs RHM1 was statistically similar to the benchmark while AI vs RHM2 was borderline inferior. The Wilcoxon matched-pairs signed rank test was used, with a two-tailed *P* < .05 indicating significance.

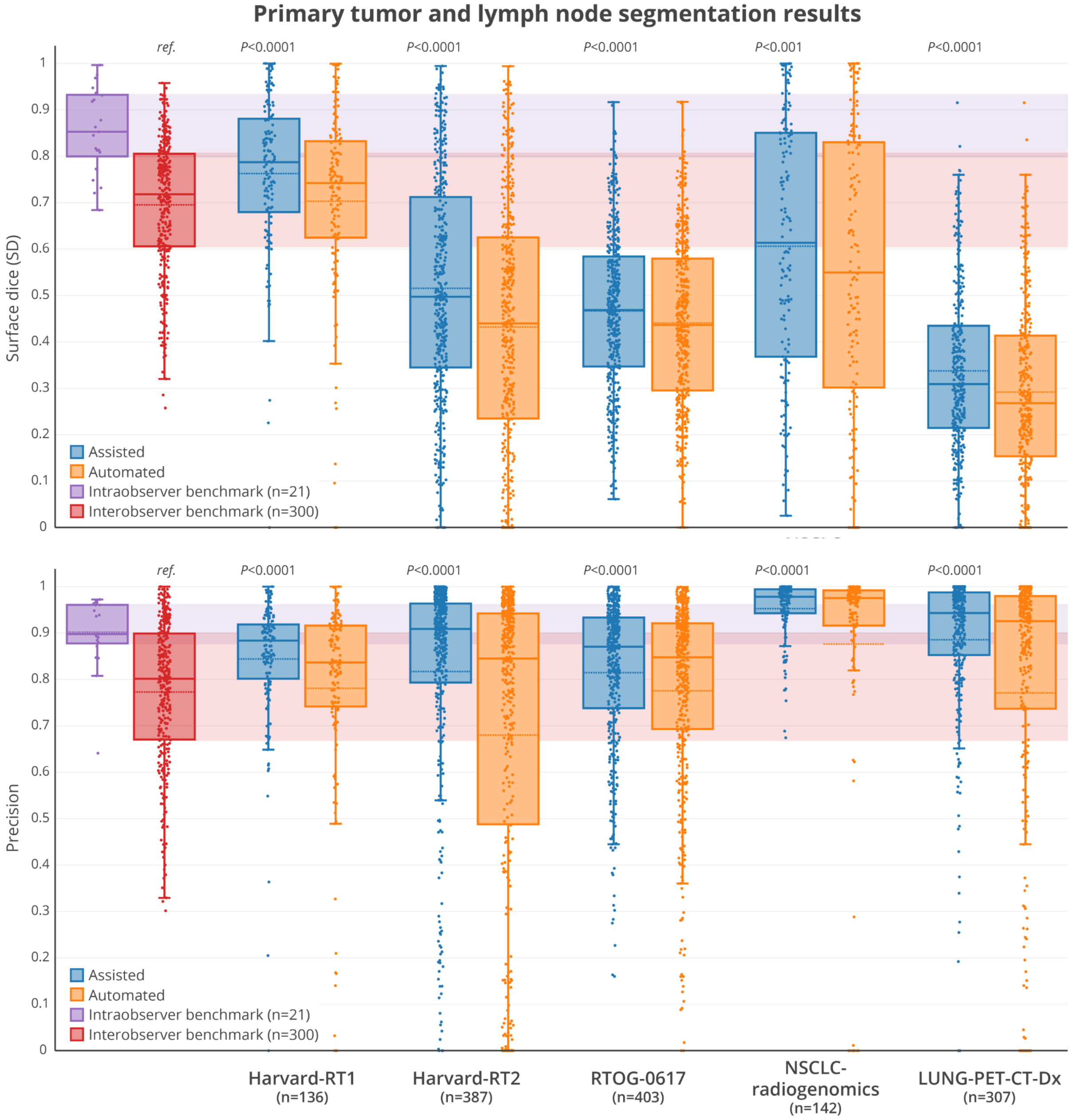

Supplementary Figure 9: Deep learning model performance in localizing and segmenting primary NSCLC tumors and involved lymph nodes, as validated on five increasingly external datasets using the surface dice (SD, top) and precision (bottom) metrics.

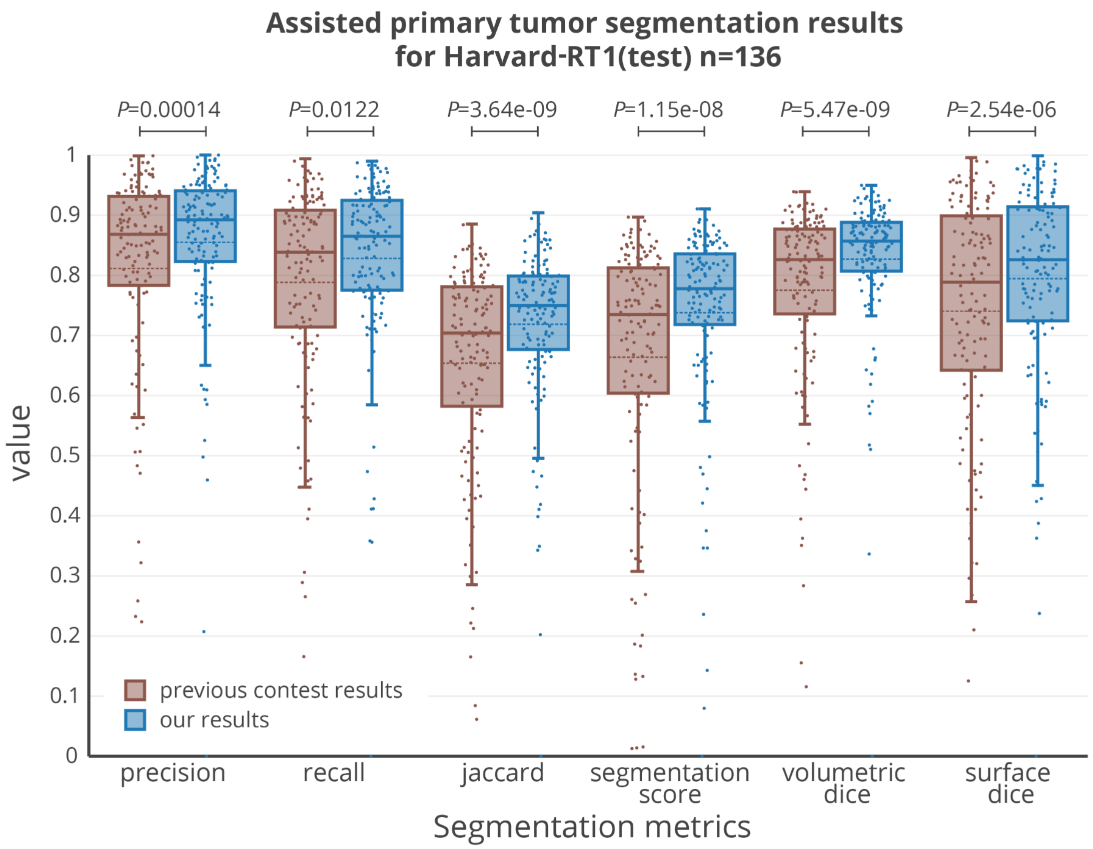

Supplementary Figure 10: Primary tumor segmentation results on 136 patients held out from the internal Harvard-RT1 dataset. Results are from the seed point assisted model. Brown box plots are results from a previously published contest on the same data[1]. Blue box plots are results from this study. The Wilcoxon matched-pairs signed rank test was used, with a two-tailed $P < .05$ indicating significance.

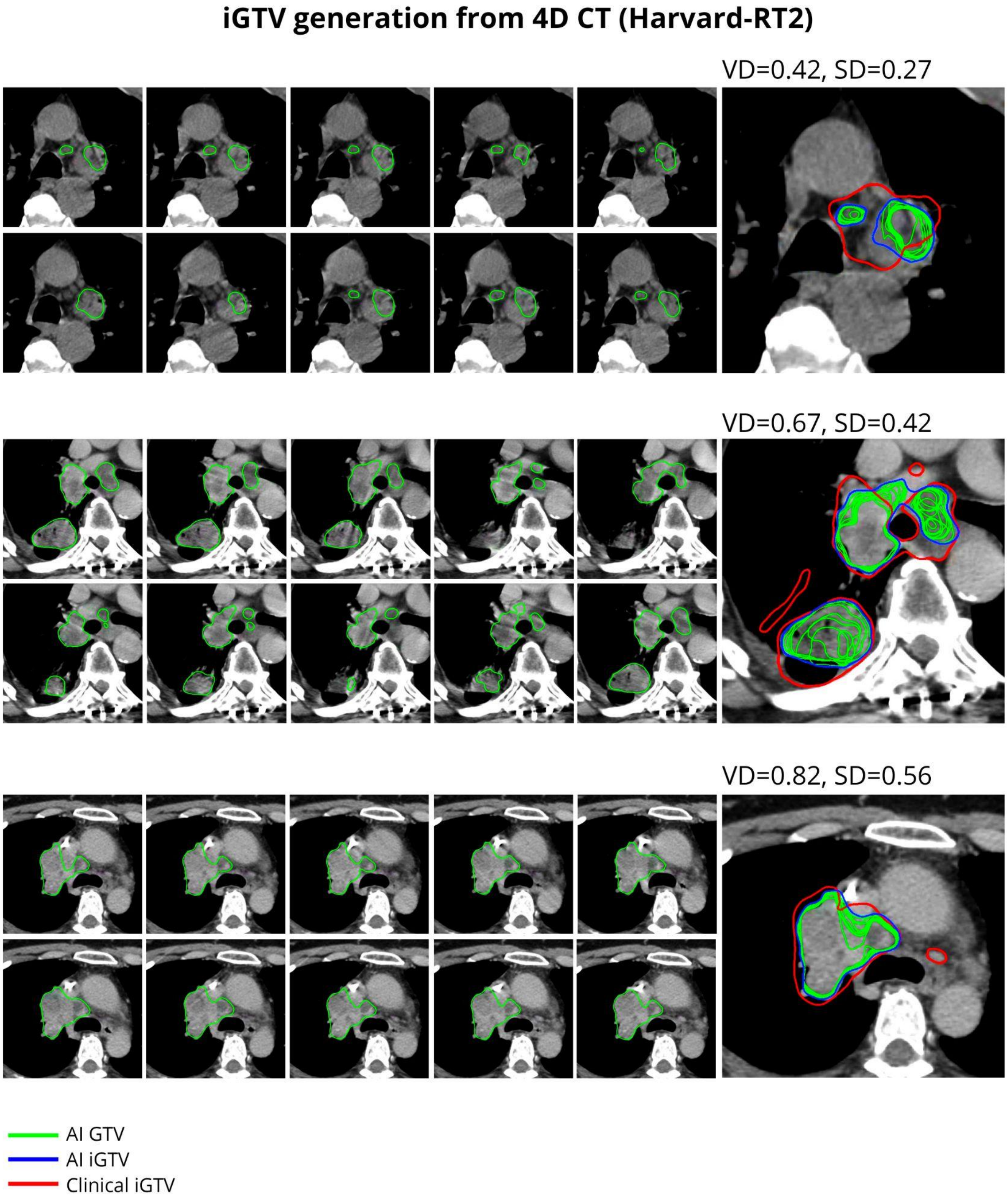

Supplementary Figure 11: Three examples from the Harvard-RT2 dataset at multiple performance levels. These illustrate the utility of the GTV segmentation model on 4D CT data. Each 4D CT comprises 10 timeframes that capture the patient's entire respiratory phase (left). The model predicts a GTV at each given timeframe (green segmentation). GTVs across all 10

timeframes are then combined to generate an iGTV, or internal GTV that compensates for the tumor's physiological movements and variation in the size, shape, and position. Panels on the right show how the AI-generated iGTV (blue segmentation) is then compared to the clinical iGTV (red segmentation). VD=volumetric dice, SD=surface dice.

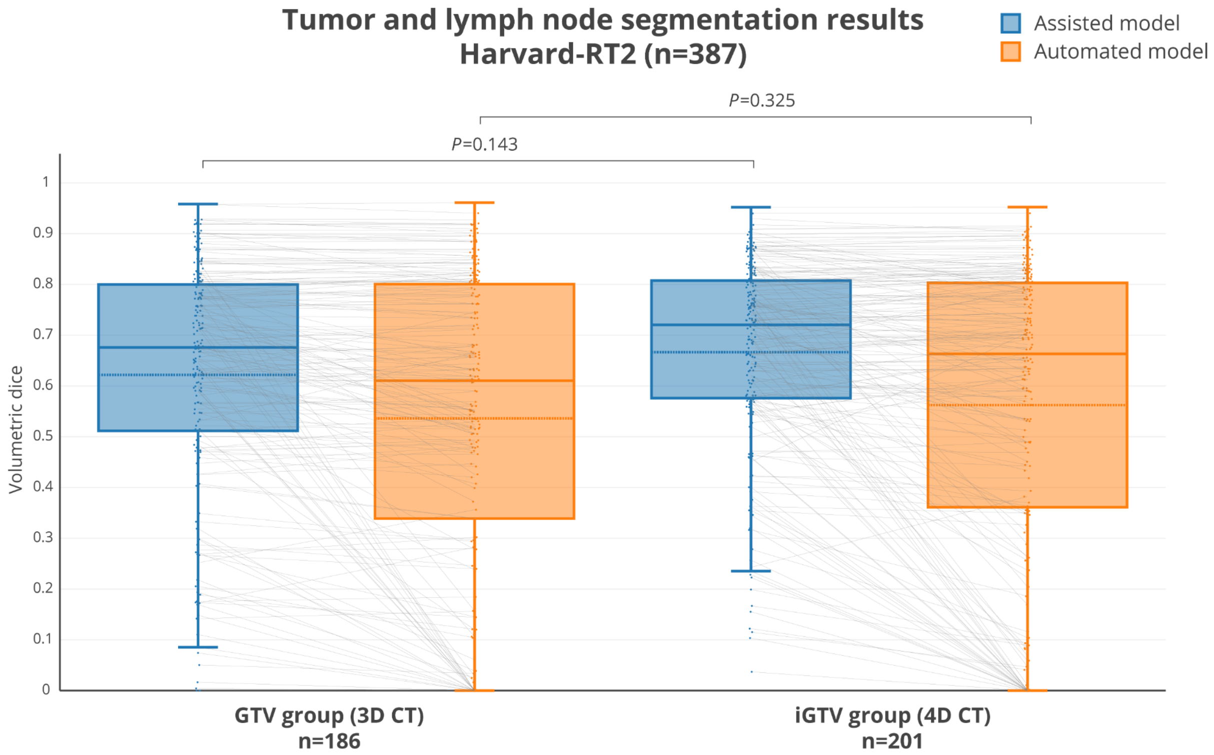

Supplementary Figure 12: Tumor and lymph node segmentation results on the Harvard-RT2 validation dataset (n=387). The GTV group (n=186) comprises single timeframe 3D CT images where the clinical GTV is compared to the AI GTV. The iGTV group (n=201) comprises 4D CT images where the clinical iGTV is compared to the AI iGTV. Non-significant differences in model performance were observed between these two groups. The Mann-Whitney U rank test was used, with a two-tailed $P < .05$ indicating significance. Blue indicates the seed point assisted model, while orange indicates the fully automated model.

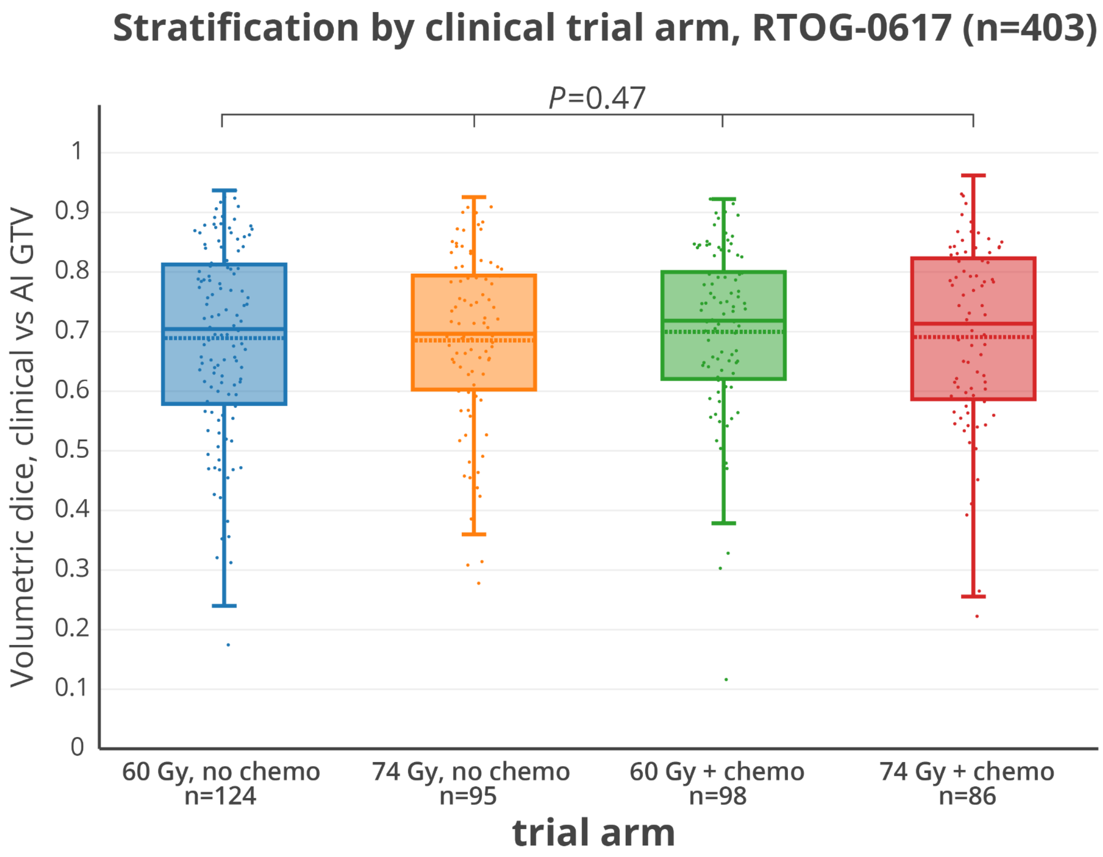

Supplementary Figure 13: Subgroup analysis on the RTOG-0617 clinical trial dataset (n=403). The plot shows non-significant differences in model performance across subgroups stratified by clinical trial arm. Model performance is defined as the volumetric dice (VD) between clinical trial GTV and AI GTV. The Kruskal-Wallis H-test was used, with a two-tailed $P < .05$ indicating significance.

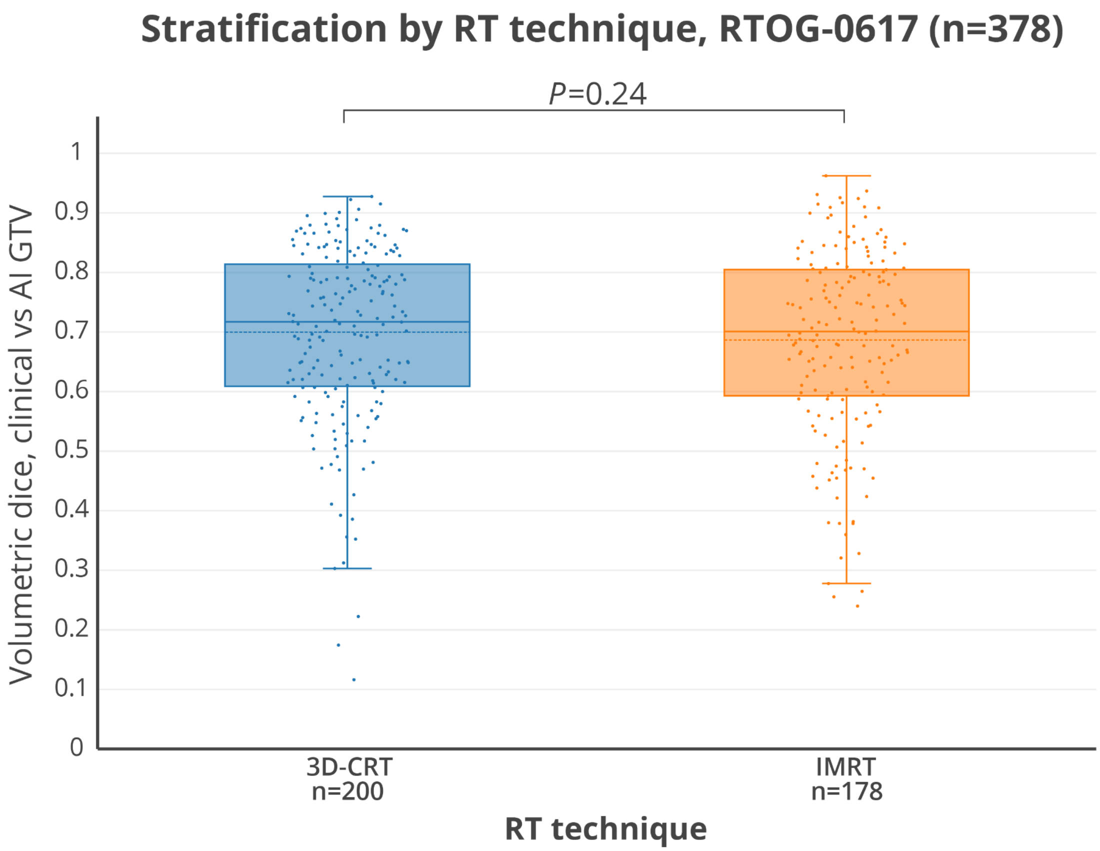

Supplementary Figure 14: Subgroup analysis on the RTOG-0617 clinical trial dataset (n=387, 25 patients did not have RT technique information). The plot shows non-significant differences in model performance across subgroups stratified by RT technique used, whether 3D conformal (3D-CRT) or intensity-modulated (IMRT). Model performance is defined as the volumetric dice (VD) between clinical trial GTV and AI GTV. The Mann-Whitney U rank test was used, with a two-tailed $P < .05$ indicating significance.

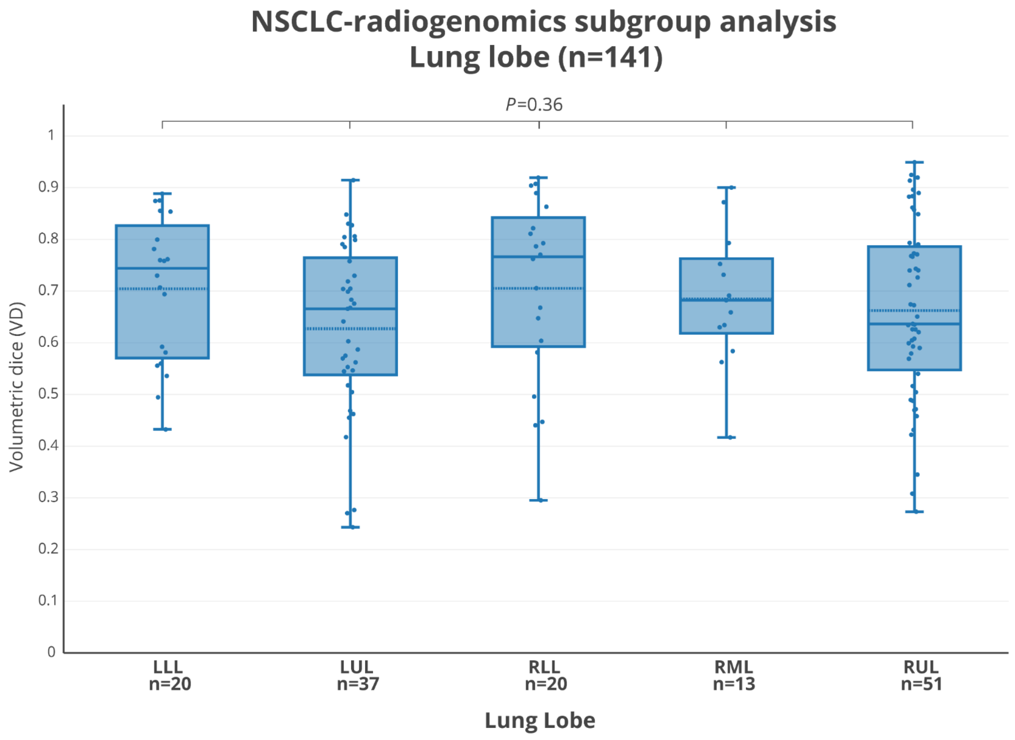

Supplementary Figure 15: Subgroup analysis of the NSCLC-radiogenomics dataset (n=141, 1 patient did not have lobe information). The plot shows non-significant differences in model performance across subgroups stratified by lung lobe. Model performance is defined as the volumetric dice (VD) between radiologist-drawn tumor segmentation and AI GTV. The Kruskal-Wallis H-test was used, with a two-tailed $P < .05$ indicating significance. LLL=left lower lobe, LUL=left upper lobe, RLL=right lower lobe, RML=right middle lobe, RUL=right upper lobe.

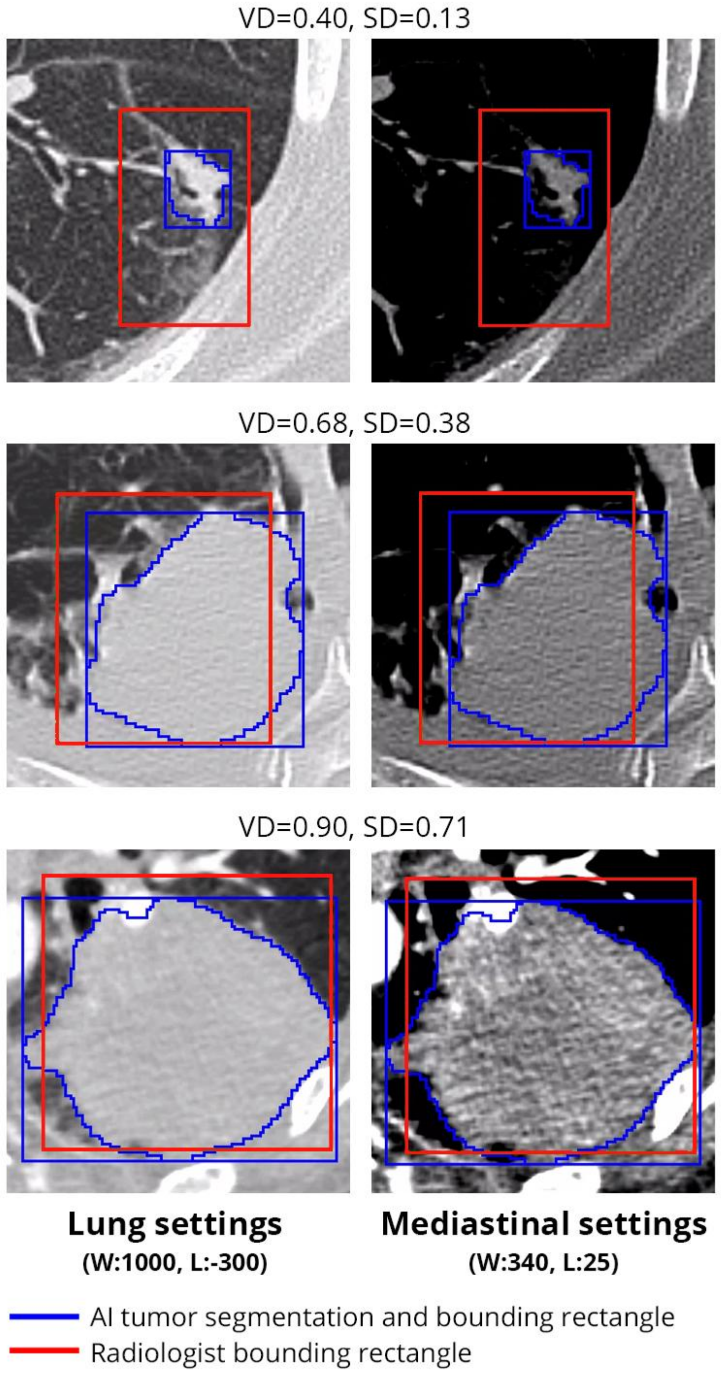

Supplementary Figure 16: Three representative examples from the Lung-PET-CT-Dx dataset accompanied by model performance metrics. Images on the left are displayed using the lung window settings, while those on the right are displayed using the mediastinal window settings. Segmentations in blue depict the AI-generated GTV and bounding rectangles (AI models trained using radiotherapy segmentations), while those in red depict bounding rectangles drawn by radiologists. VD=volumetric dice, SD=surface dice.

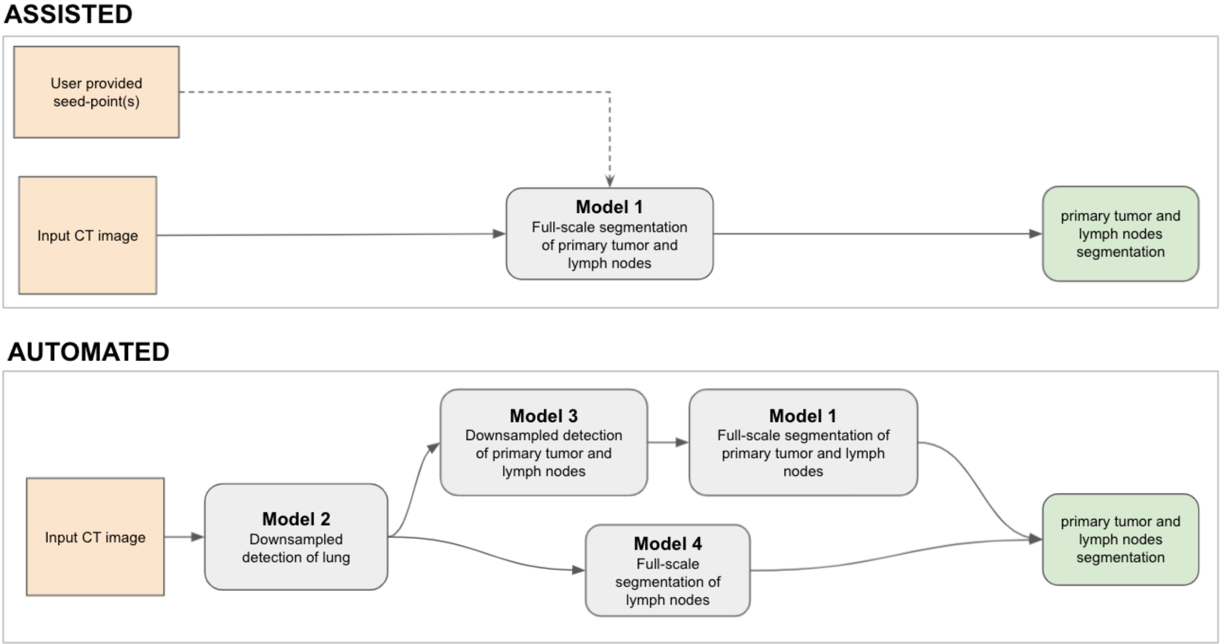

Supplementary Figure 17: Figure depicting the assisted and automated pipelines and the four models that comprise them. In the assisted pipeline, a user-placed seed point helps identify the region of interest to be cropped and fed into the model. In the automated pipeline, detection models are used to identify the region of interest. For model specifications, see Supplementary Table 6.

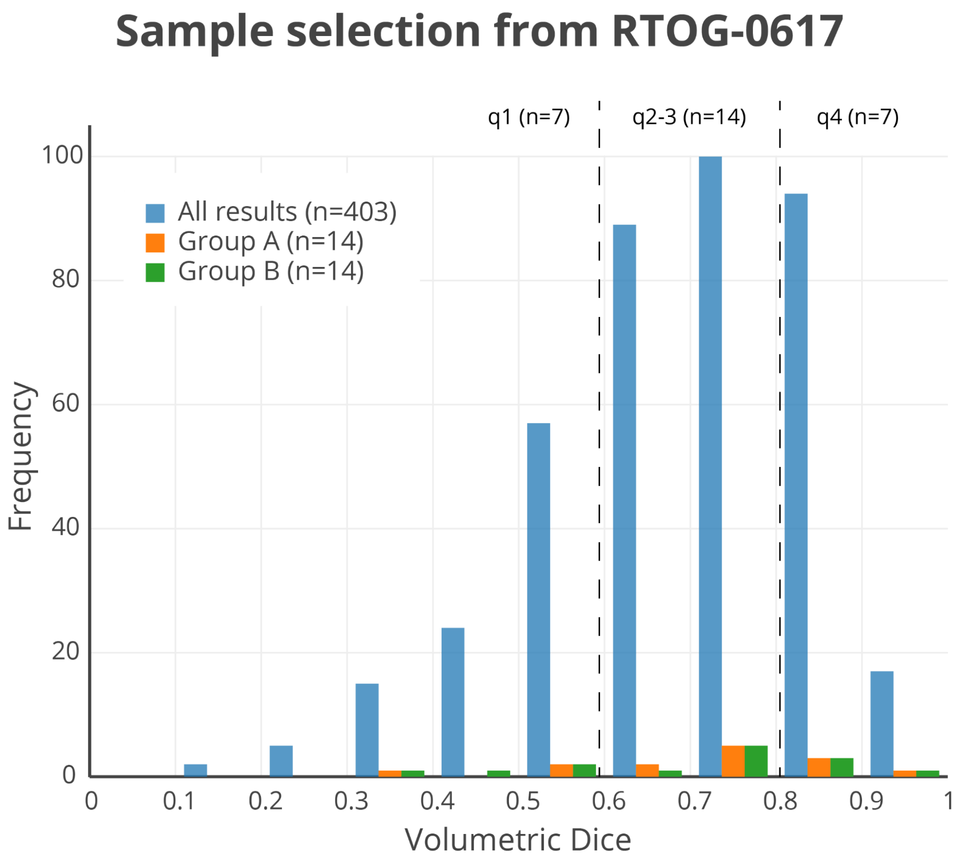

Supplementary Figure 18: A quartile-based random subset of 28 patients is selected from the RTOG-0617 dataset. This subset is used for the dosimetric analysis, as well as the end-user testing where the subset is further divided into two groups, A and B.

0617-713112	GTV		PTV			
	VD	SD	VD	SD	v95	d95
Clinical	0.55	0.25	0.74	0.14	99.00	59.38
AI					99.24	60.71

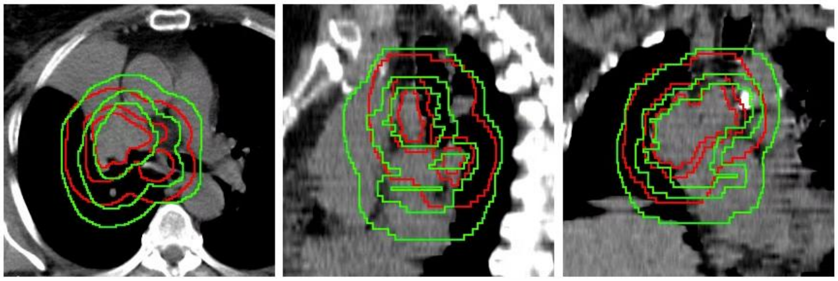

0617-619906	GTV		PTV			
	VD	SD	VD	SD	v95	d95
Clinical	0.78	0.33	0.80	0.23	99.22	72.37
AI					99.37	72.46

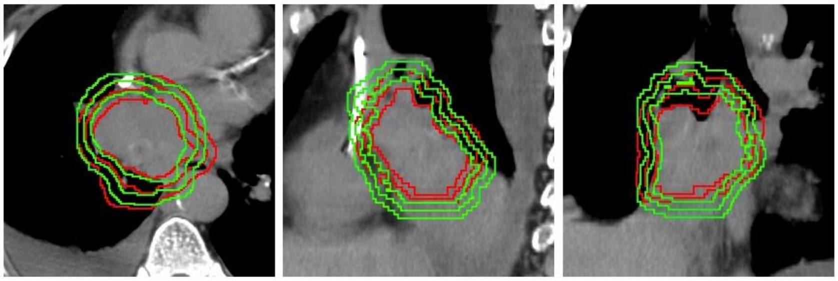

0617-657637	GTV		PTV			
	VD	SD	VD	SD	v95	d95
Clinical	0.91	0.75	0.71	0.02	97.20	71.52
AI					70.46	50.40

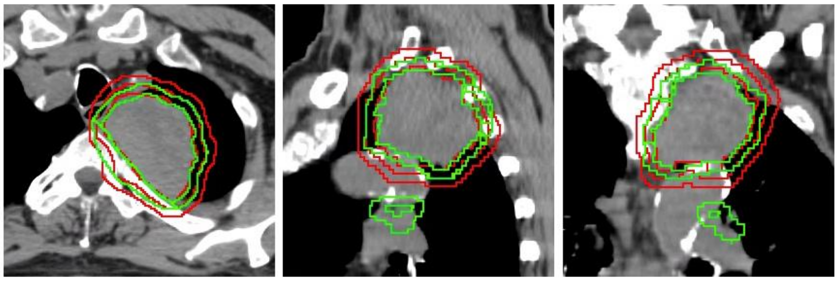

Supplementary Figure 19: Three representative examples from a subset of the RTOG-0617 dataset showing model performance metrics (table) together with axial, sagittal, and coronal views. Concentric segmentations donate the gross tumor volume (GTV, innermost), clinical tumor volume (CTV, intermediate), and planning target volume (PTV, outermost). This figure illustrates the dosimetric analysis results by comparing the ground truth PTV as used in the

RTOG-0617 clinical trial with its AI-generated counterpart. The first example shows a case with sub-optimal overlap performance - low VD and SD - but with great dose coverage as measured by the v95 and d95 metrics. The second example shows better overlap measures and also great dose coverage. Finally, the third example shows superior overlap measures with sub-optimal dose metrics. VD=volumetric dice, SD=surface dice, v95= percent target volume that received at least 95% of the prescription dose, d95=dose covering 95% of the target volume.

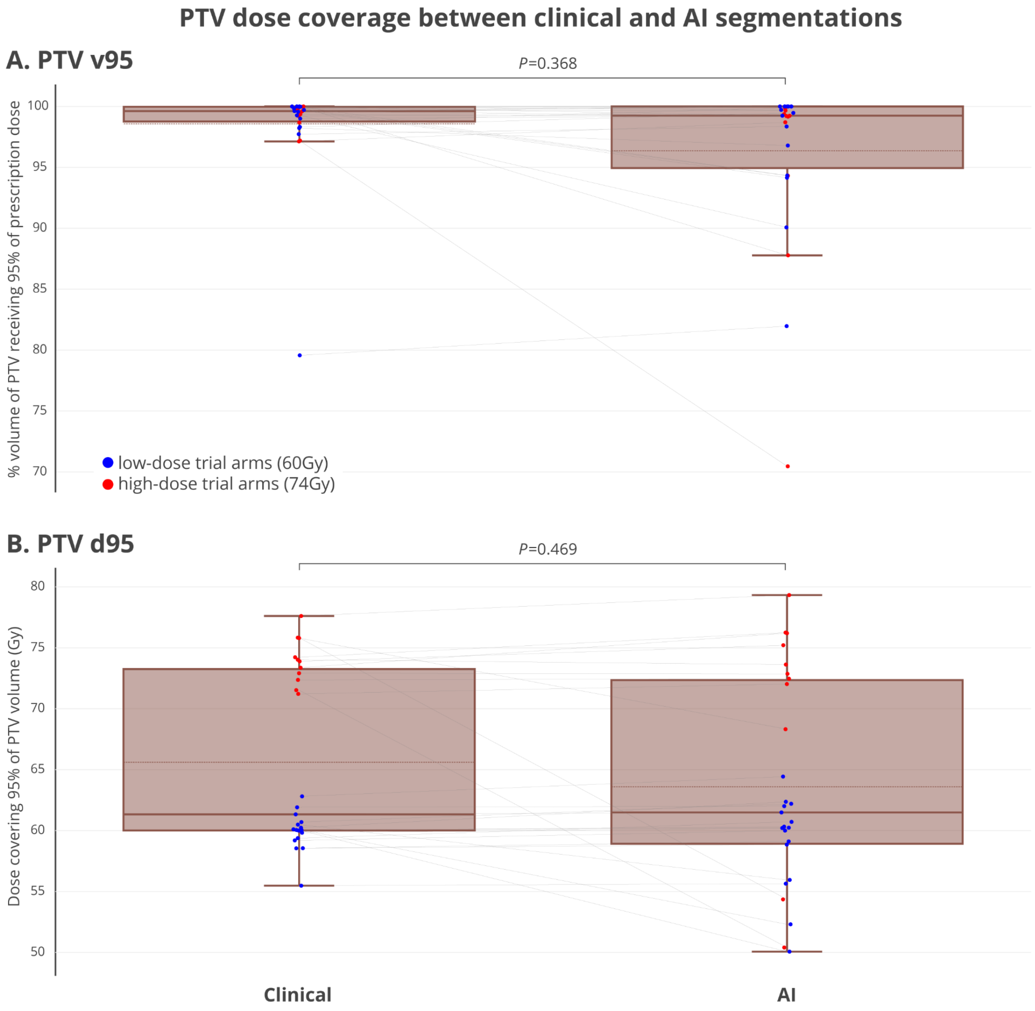

Supplementary Figure 20: Dose coverage in n=28 patient subset of the RTOG-0617 dataset illustrating the difference between ground truth planning target volume (PTV) as used in the RTOG-0617 clinical trial with its AI-generated counterpart. Panel A shows the v95 metric, or the percent target volume that received at least 95% of the prescription dose. Panel B shows the d95 metric, or the dose covering 95% of the target volume. Non-significant differences were found between dose coverage of clinical trial PTV and AI PTV, for both metrics. The Wilcoxon matched-pairs signed rank test was used, with a two-tailed $P < .05$ indicating significance. Dots in blue represent the low-dose arms (arms 1 and 3, 60Gy, n=16) and the high-dose arms (arms 2 and 4, 74Gy, n=12) of the RTOG-0617 clinical trial. For more information about this trial, see[3].

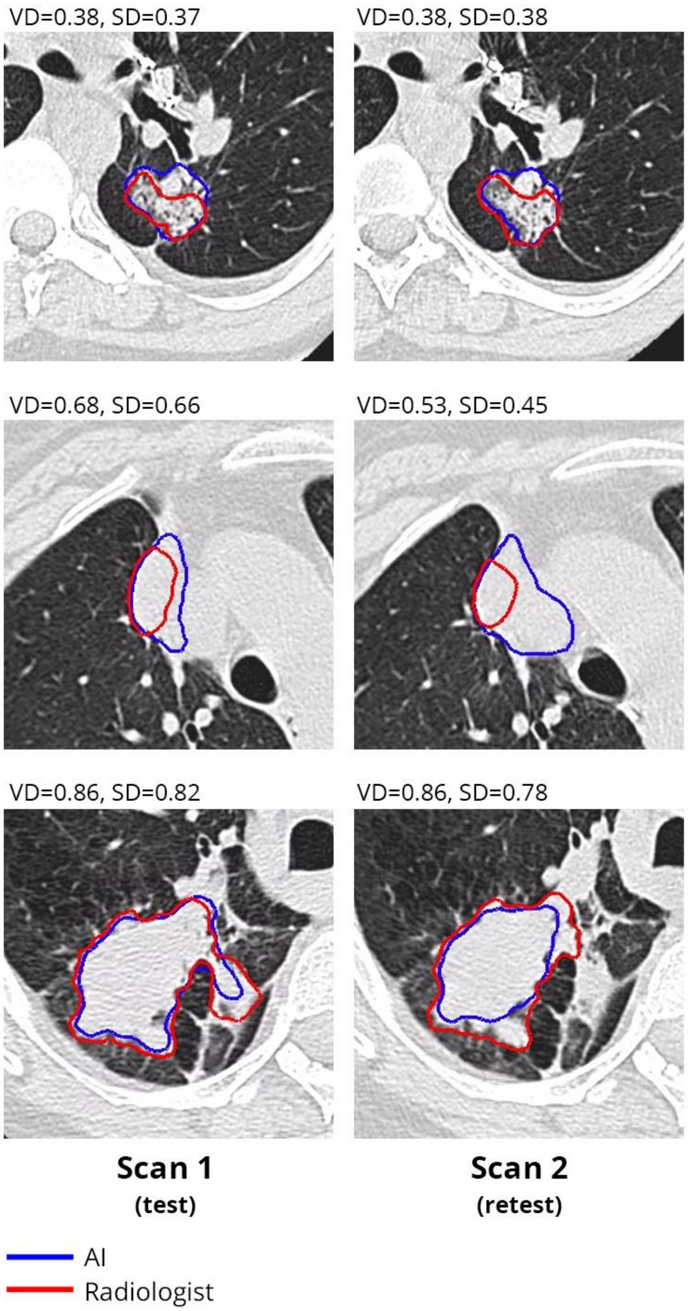

Supplementary Figure 23: Three examples from the RIDER dataset at multiple performance levels. On the left are images and segmentations from the first scan (test scan), while images and segmentations from the second scan (retest scan, taken 15 minutes later) are shown on the right. Scans were independent of one another and acquired separately. Best effort was made to align the axial slices for comparison. The AI-generated segmentations are shown in blue, while the radiologists' segmentations are shown in red. VD=volumetric dice, SD=surface dice.

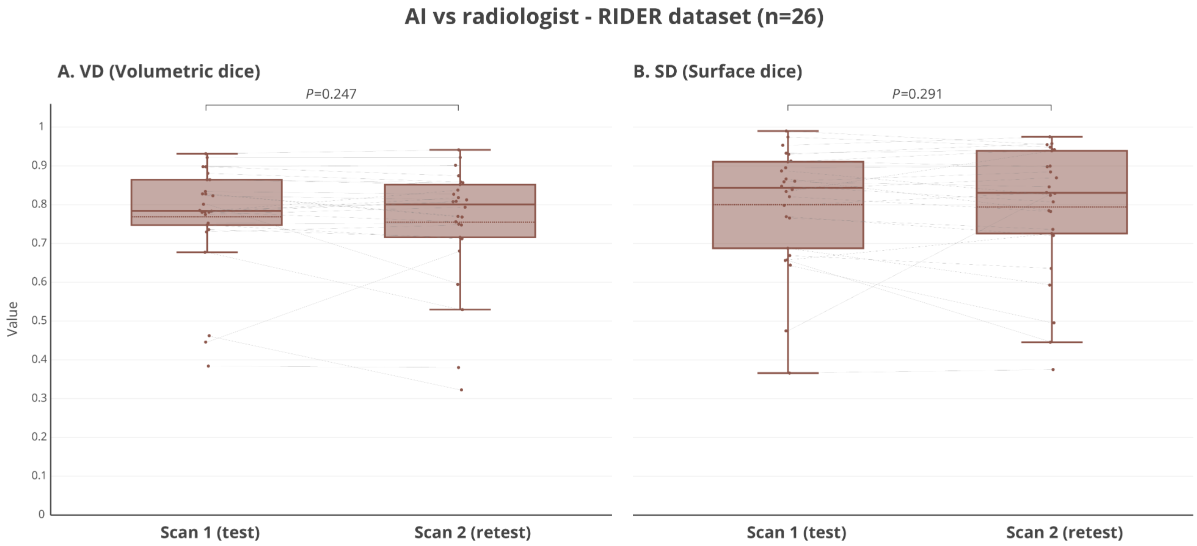

Supplementary Figure 24: Comparison between AI and radiologists' tumor segmentations on the RIDER dataset (n=26) for both scan 1 (test) and scan 2 (retest). For both volumetric dice (panel A) and surface dice (panel B), non-significant differences were found between the two scans. The Wilcoxon matched-pairs signed rank test was used, with a two-tailed P < .05 indicating significance.

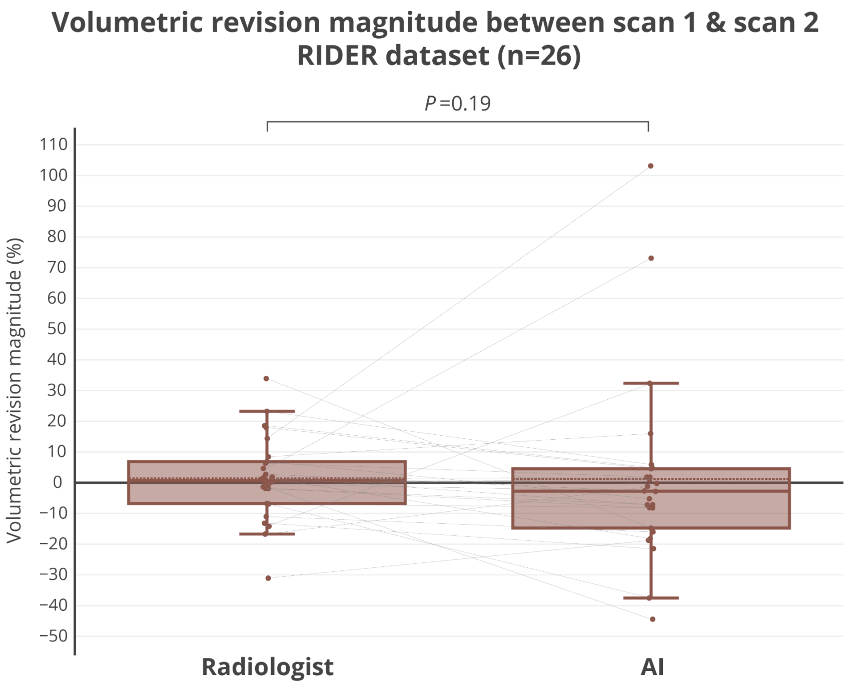

Supplementary Figure 25: Variation in volumetric revision magnitudes between scan 1 and scan 2 of the RIDER dataset (n=26). Results are shown for radiologists' and AI tumor segmentations. Radiologists' variation in tumor volume across the two scans was non-significantly different from that of the AI models. Volumetric revision magnitude is defined as the edited volume of scan 2 divided by the volume of scan 1, multiplied by 100. The Wilcoxon matched-pairs signed rank test was used, with a two-tailed $P < .05$ indicating significance.

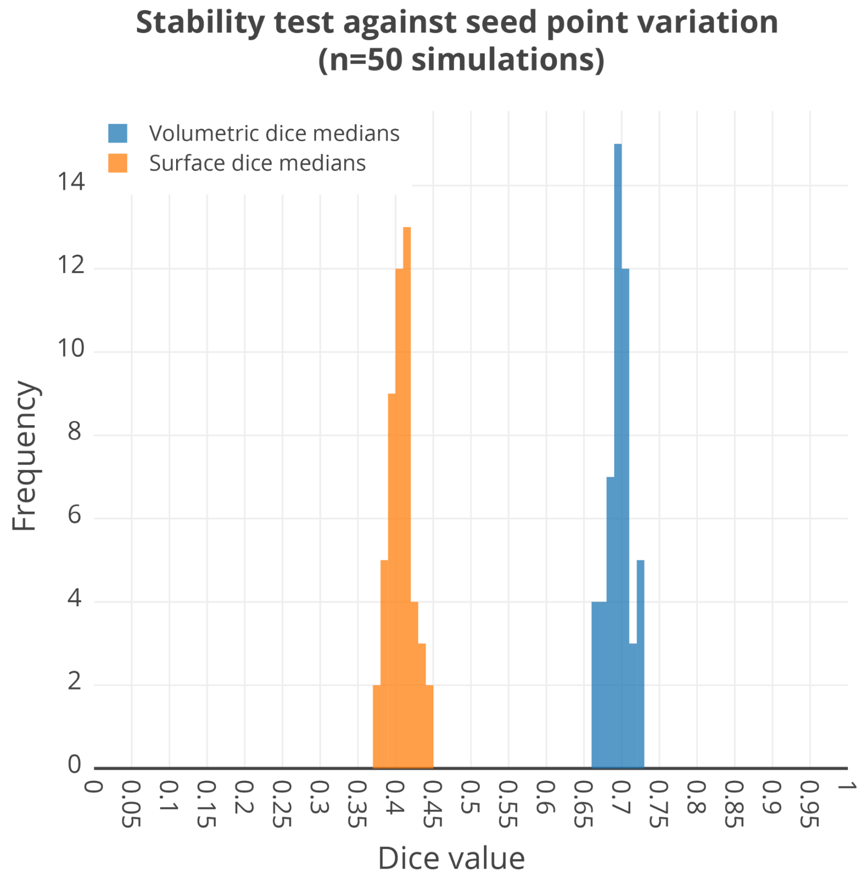

Supplementary Figure 26: 50 simulations to test the assisted models' stability as a function of variation in input data. This experiment was conducted on a random quartile-based 28 patient subset of the RTOG-0617 clinical trial dataset (Supplementary Figure 18). For each simulation, the seed point was randomly relocated in 3D space within a 50x50x50mm region surrounding the center of mass of the clinical segmentations. This was performed on all 28 patients, with the median volumetric dice (VD) and surface dice (SD) reported here. Median model predictions showed high stability with an inter quartile range (IQR) of 0.02 for both VD and SD.

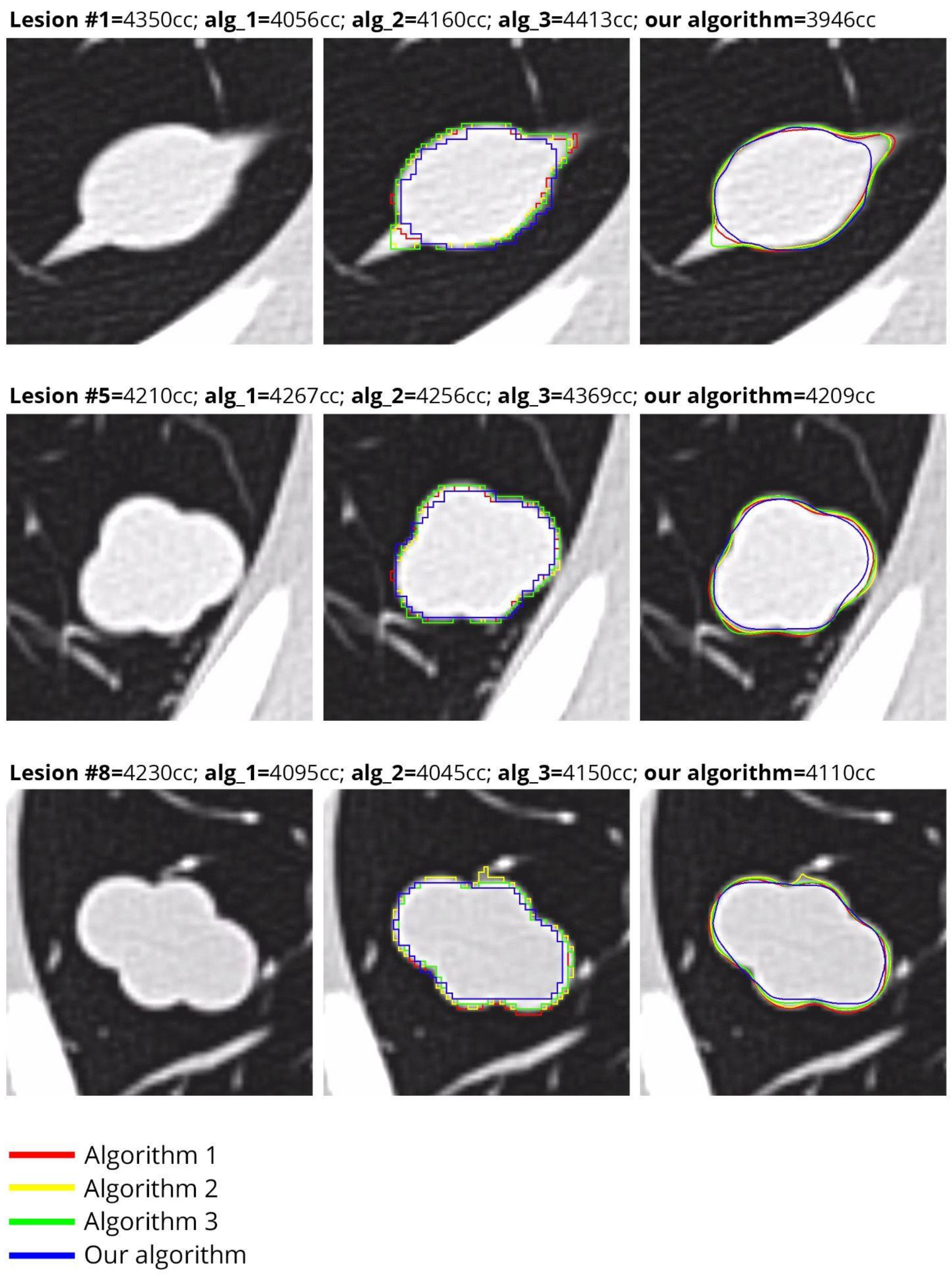

Supplementary Figure 27: Three example lesions from the thorax phantom dataset. The left column shows the synthetic lesions without segmentations. The middle column shows the segmentations overlaid in their original binary format. The right column shows the segmentations overlaid in a smoothed spline-based format (clinical software often performs similar smoothing on segmentations). The segmentations represent output from four different algorithms: three previously published algorithms[8] in addition to ours. All algorithms tend to underestimate the true known volume of the synthetic lesions.

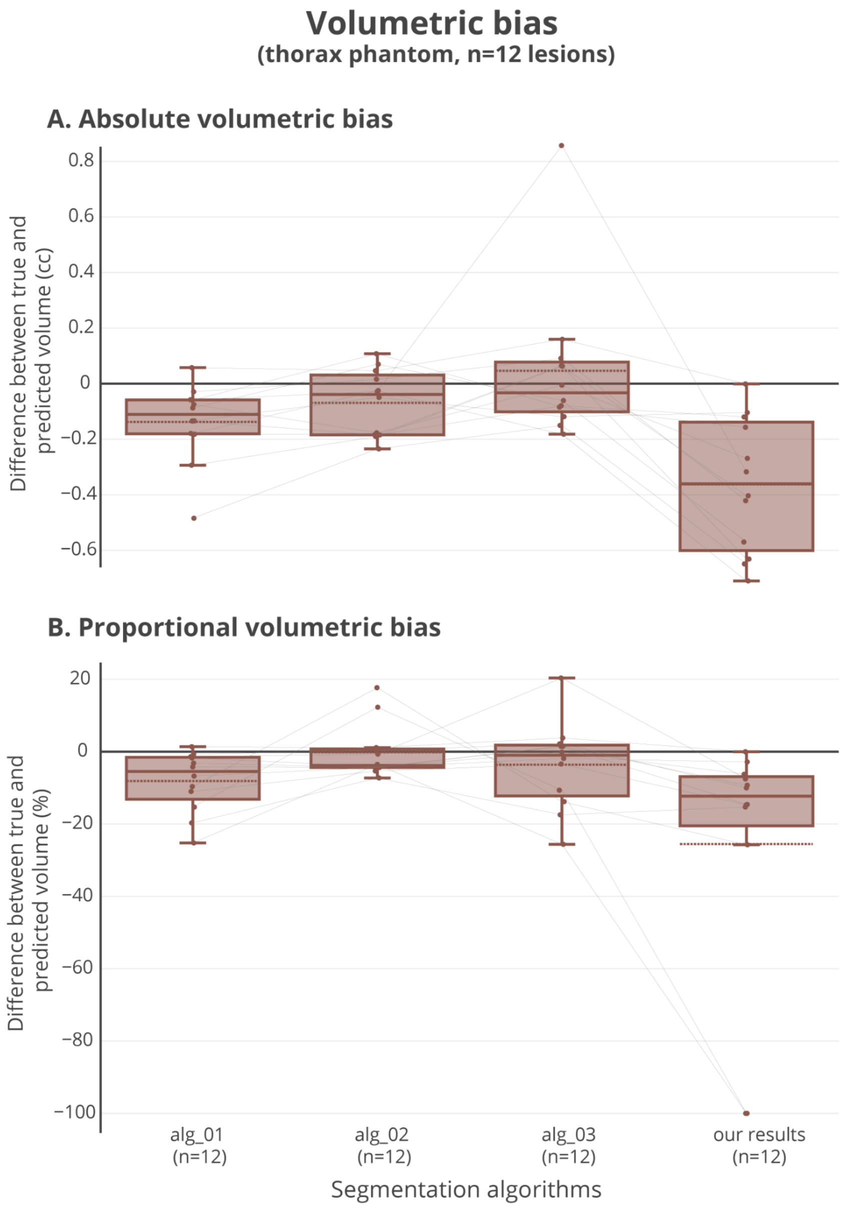

Supplementary Figure 28: Difference between true and predicted lesion volume in the thorax phantom dataset (n=12 lesions). Results are shown for four different segmentation algorithms: three previously published algorithms[8] in addition to ours. Panel A shows absolute bias calculated as predicted minus true volume, in cc. Panel B shows proportional bias calculated as the absolute bias divided by the true volume, presented as a percentage. All algorithms tend to underestimate the true known volume of the synthetic lesions. The small sample size (n=12) did not provide enough confidence in running statistical tests between the different algorithms.

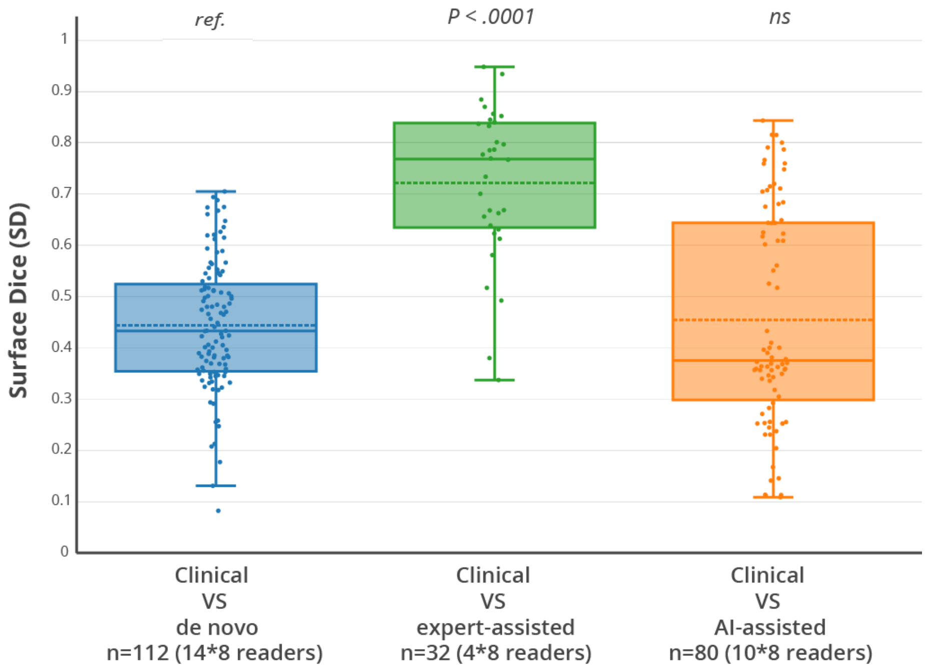

Supplementary Figure 29: Results from the end-user testing reporting the surface dice score between clinical trial segmentations and each of *de novo*, expert-assisted, and AI-assisted segmentations. The Mann-Whitney U rank test was used, with a two-tailed $P < .05$ indicating significance.

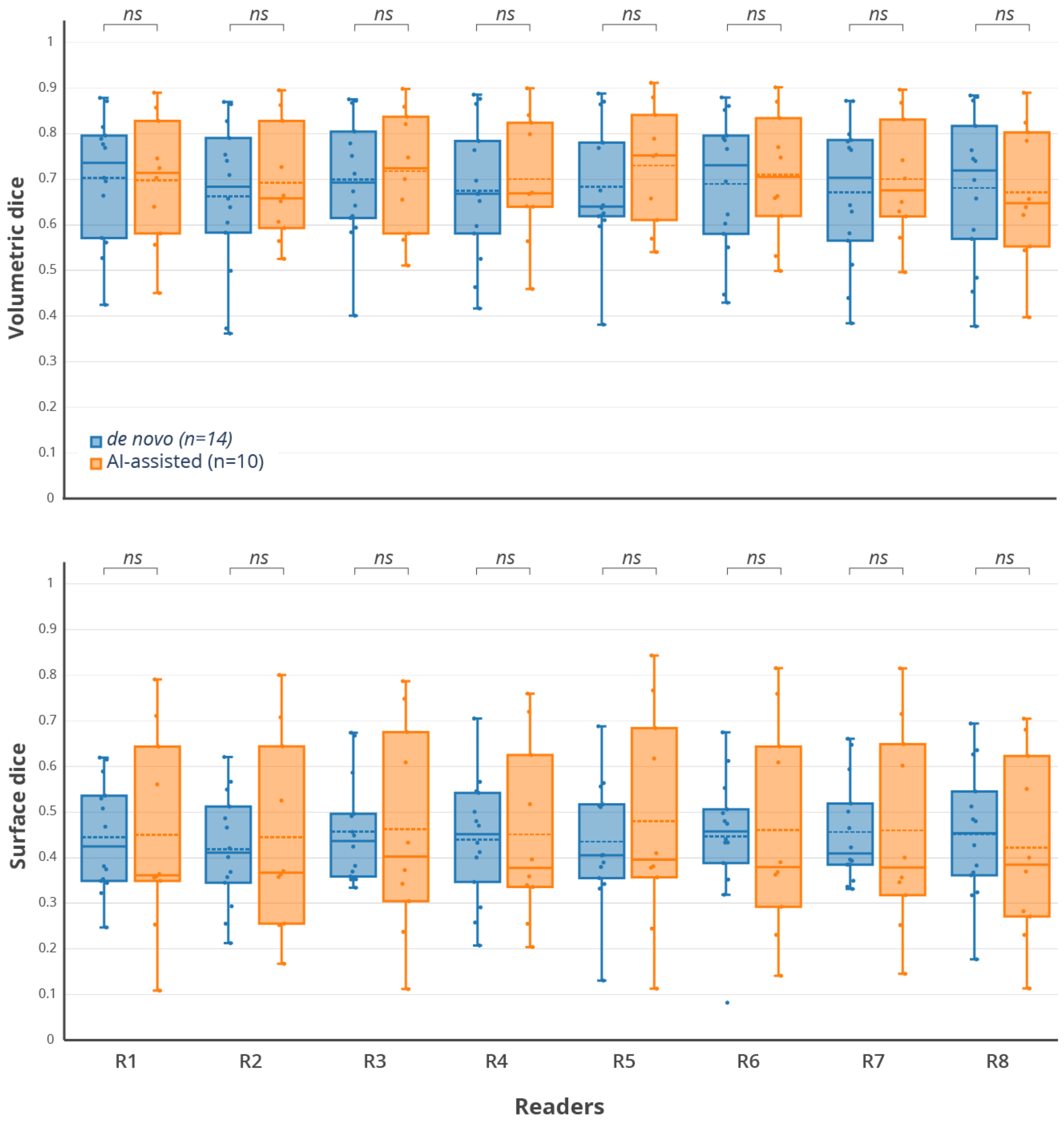

Supplementary Figure 30: Results from the end-user testing comparing de novo and AI-assisted segmentations per reader. The Mann-Whitney U rank test was used, with a two-tailed $P < .05$ indicating significance.

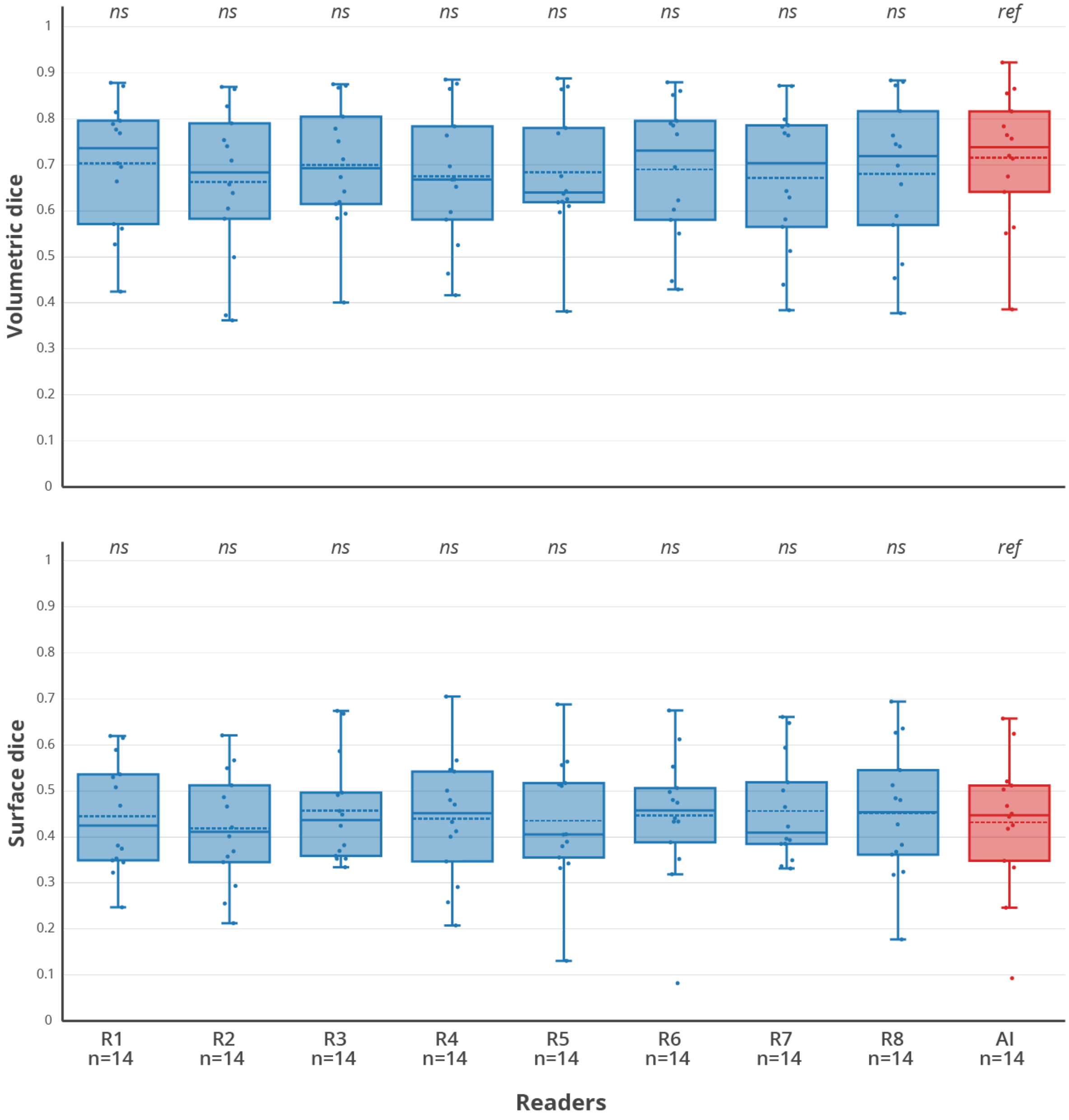

Supplementary Figure 31: Results from the end-user testing for cases segmented *de novo*. Segmentation metrics are calculated between clinical and readers' de novo segmentations in blue, as well as between clinical and AI segmentations in red. The Wilcoxon matched-pairs signed rank test was used, with a two-tailed $P < .05$ indicating significance.

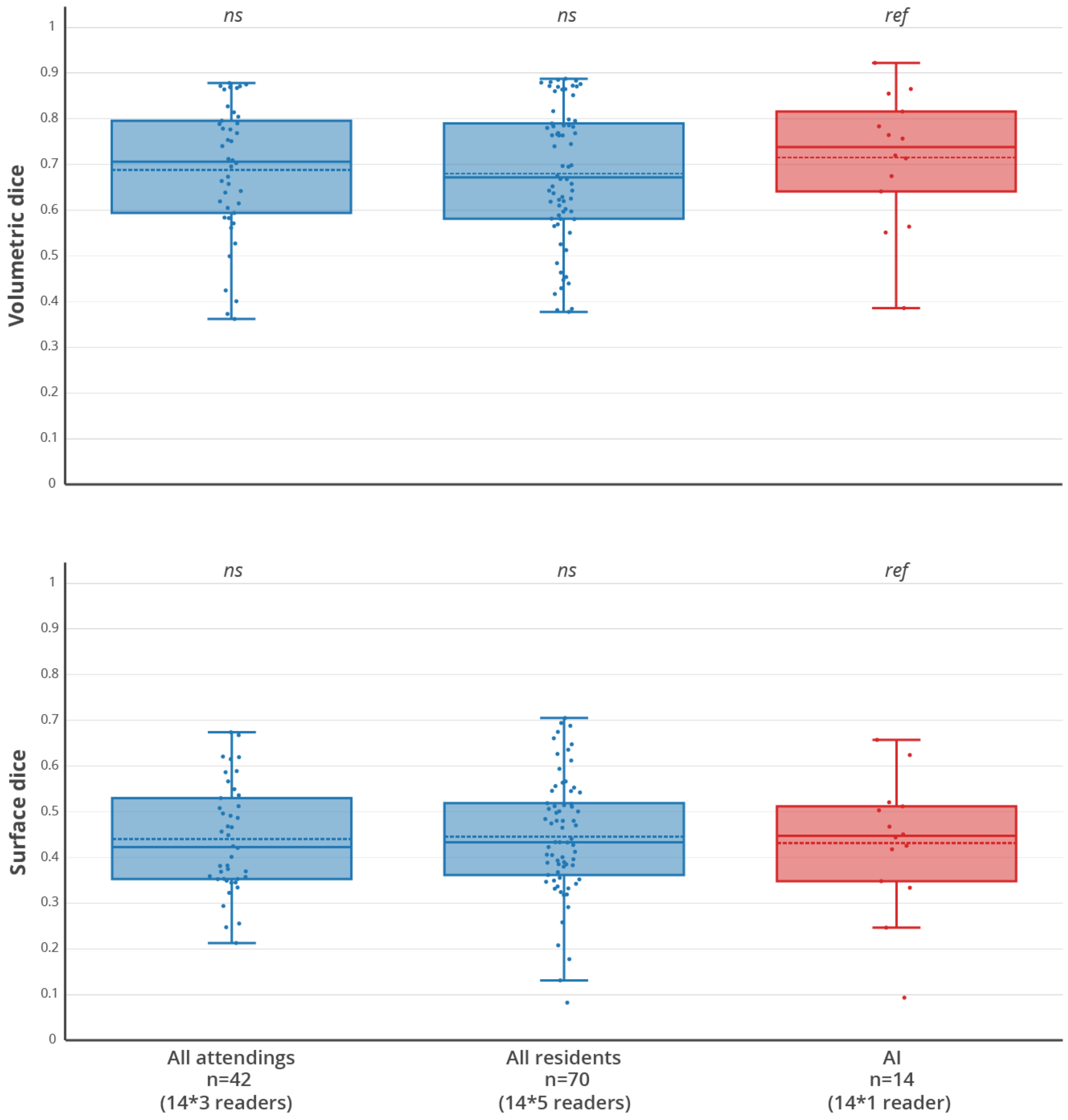

Supplementary Figure 32: Results from the end-user testing for cases segmented *de novo*. Segmentation metrics are calculated between clinical and readers' de novo segmentations in blue (3 attendings and 5 residents), as well as between clinical and AI segmentations in red. The Mann-Whitney U rank test was used, with a two-tailed $P < .05$ indicating significance.

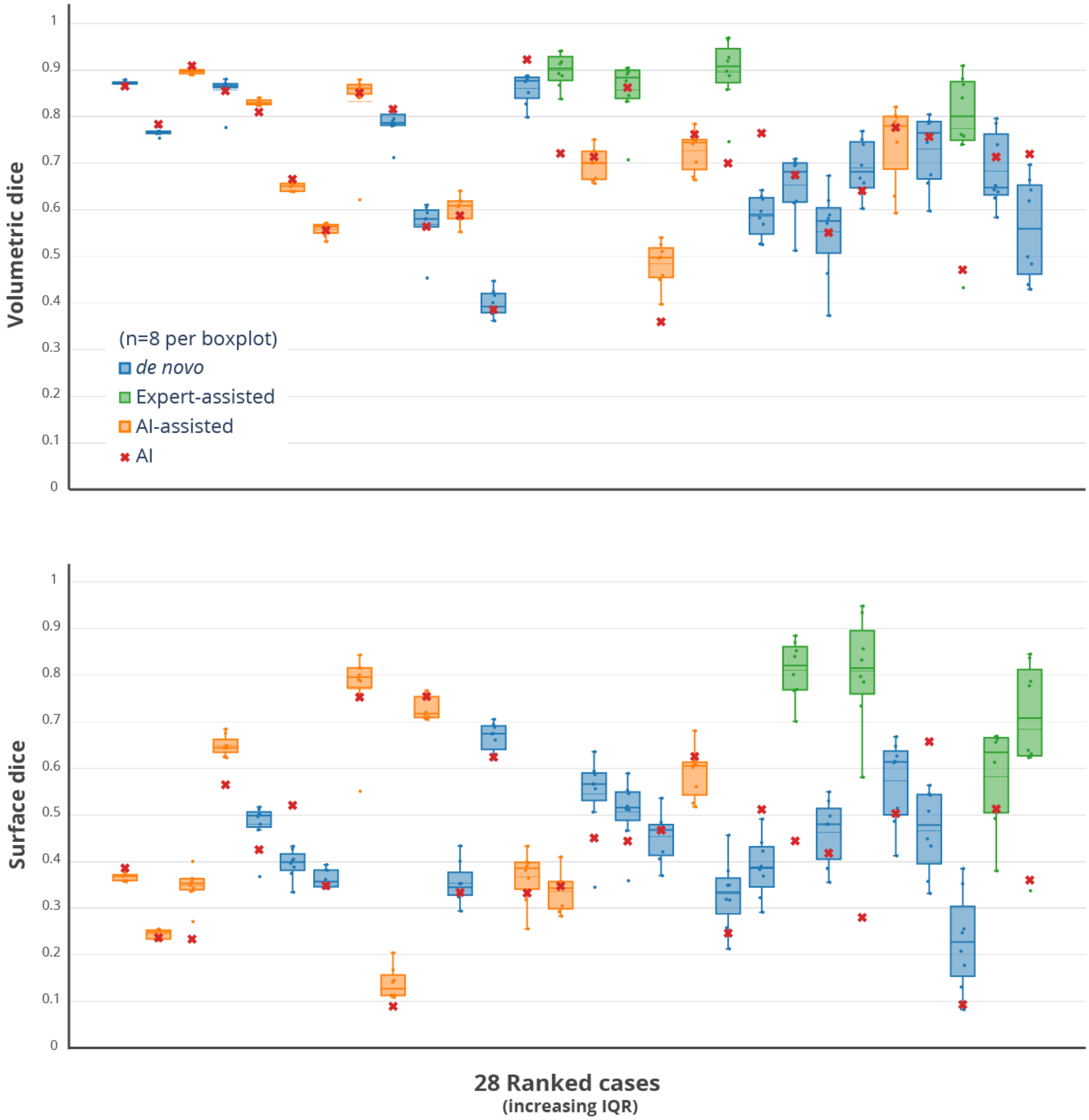

Supplementary Figure 33: Results from the end-user testing displaying metrics per case. Cases are ranked based on increasing interquartile range of the respective segmentation metric. Metrics are calculated between clinical trial segmentations and each of de novo, expert-assisted, AI-assisted, and AI segmentations as shown in the legend. Each boxplot contains 8 metric values corresponding to the 8 readers recruited for this experiment.

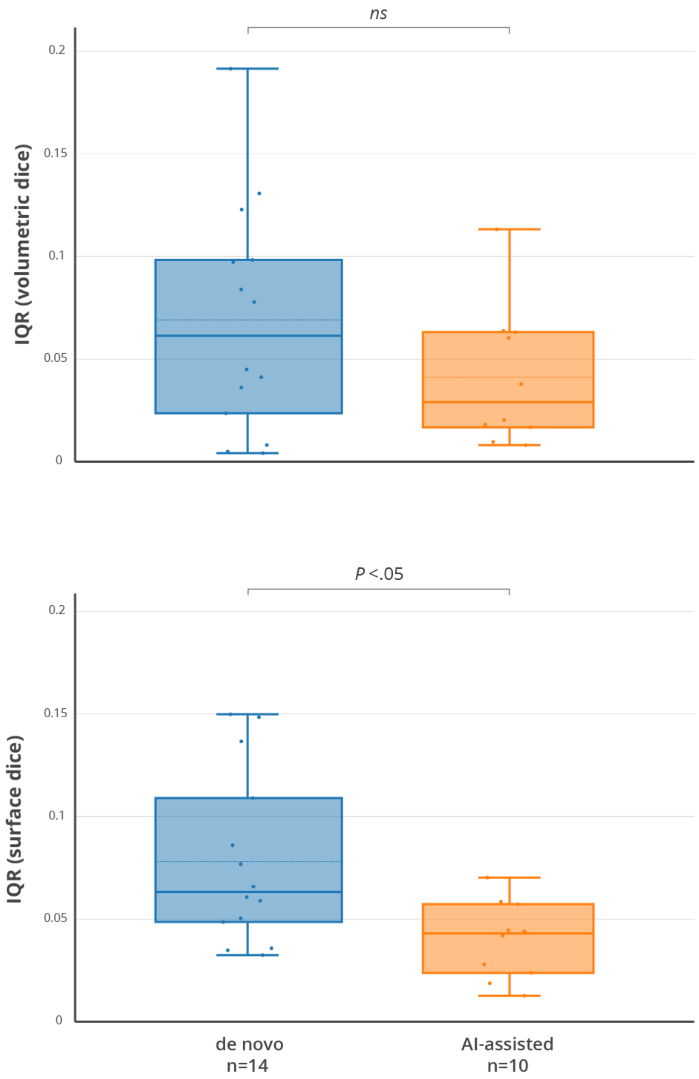

Supplementary Figure 34: Results from the end-user testing displaying differences in interquartile ranges between de novo and AI-assisted cases. Interquartile ranges are calculated on a case by case basis and measures the amount of variability among the 8 readers in the experiment.

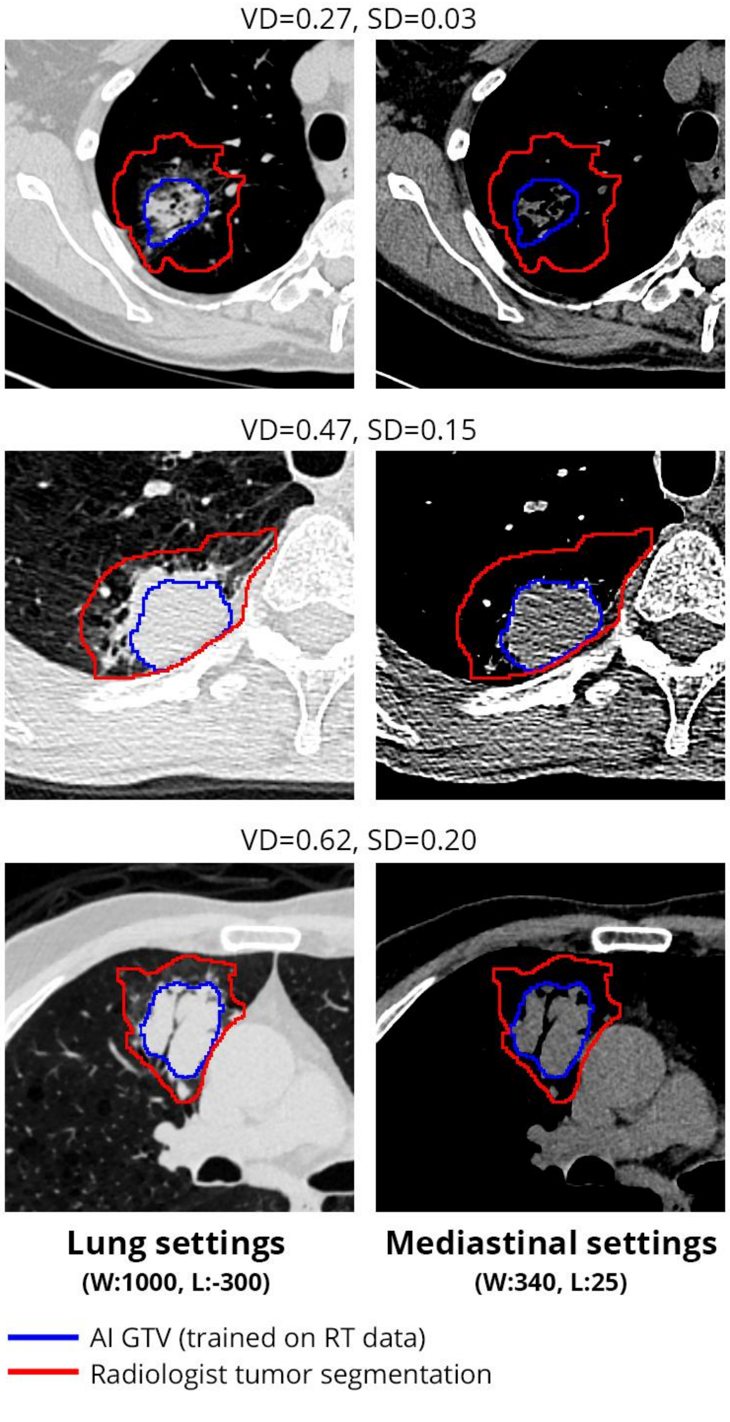

Supplementary Figure 35: Three representative examples from the NSCLC-radiogenomics dataset accompanied by model performance metrics. Images on the left are displayed using the lung window settings, while those on the right are displayed using the mediastinal window settings. Segmentations in blue depict the AI-generated GTV (AI models trained using radiotherapy segmentations), while those in red depict segmentations performed by radiologists. VD=volumetric dice, SD=surface dice.

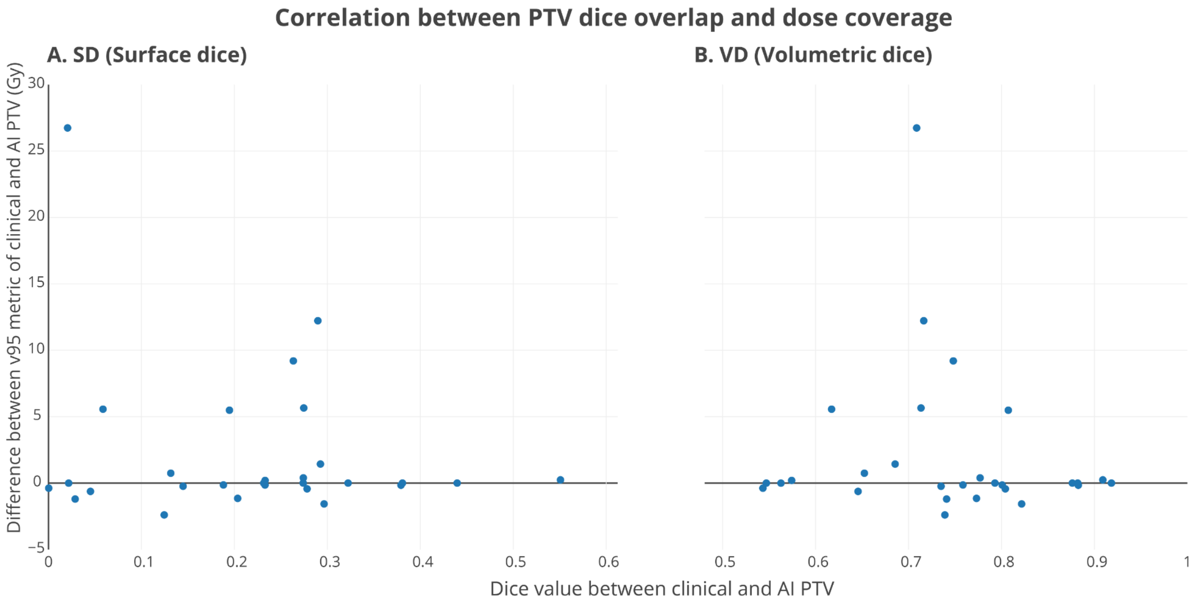

Supplementary Figure 36: Correlation between the v95 difference between clinical and AI PTV and the dice agreement between them. Data is from a n=28 patient subset of the RTOG-0617 clinical trial dataset Panel A shows v95 difference against surface dice, Spearman R=0.17, *P*=0.38. Panel B shows v95 difference against volumetric dice, Spearman R=-0.12, *P*=0.55.

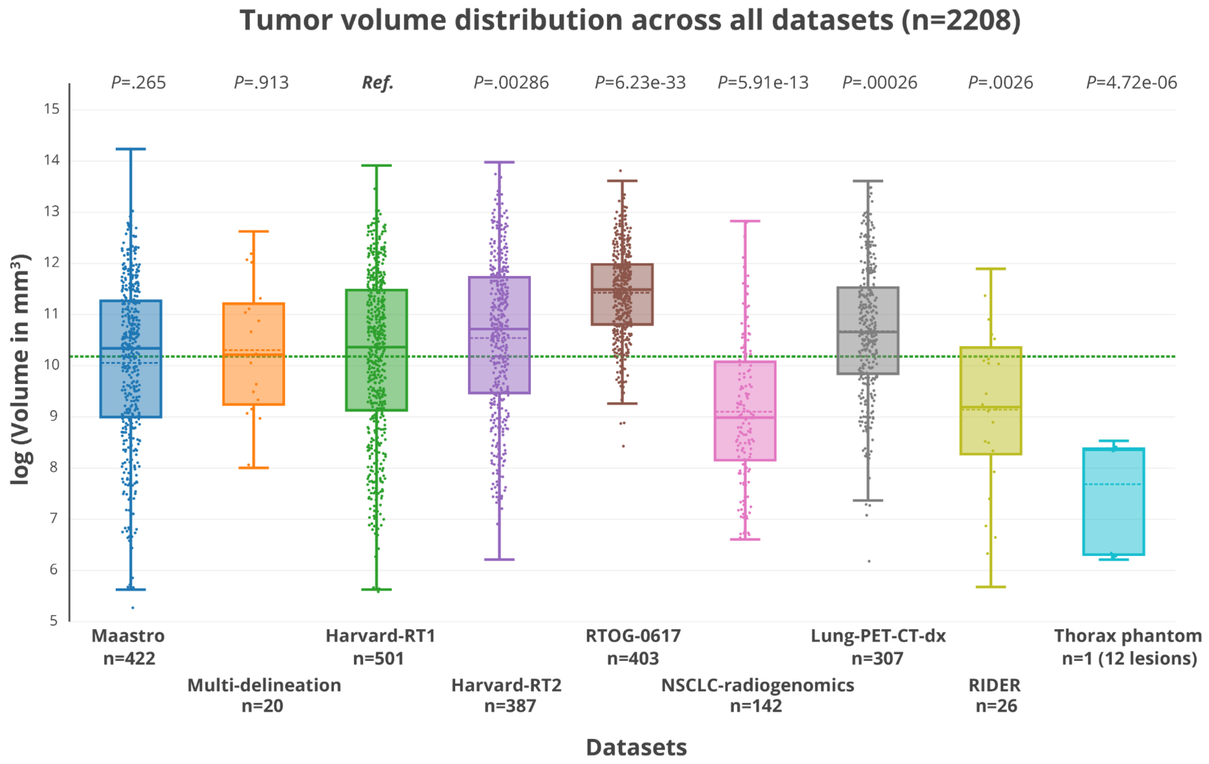

Supplementary Figure 37: Figure depicting tumor and involved lymph node volume distribution across all data used in this study. Volume is displayed on the log scale. Statistical test was conducted against Harvard-RT1 as the reference. The Mann-Whitney U rank test was used, with a two-tailed $P < .05$ indicating significance.

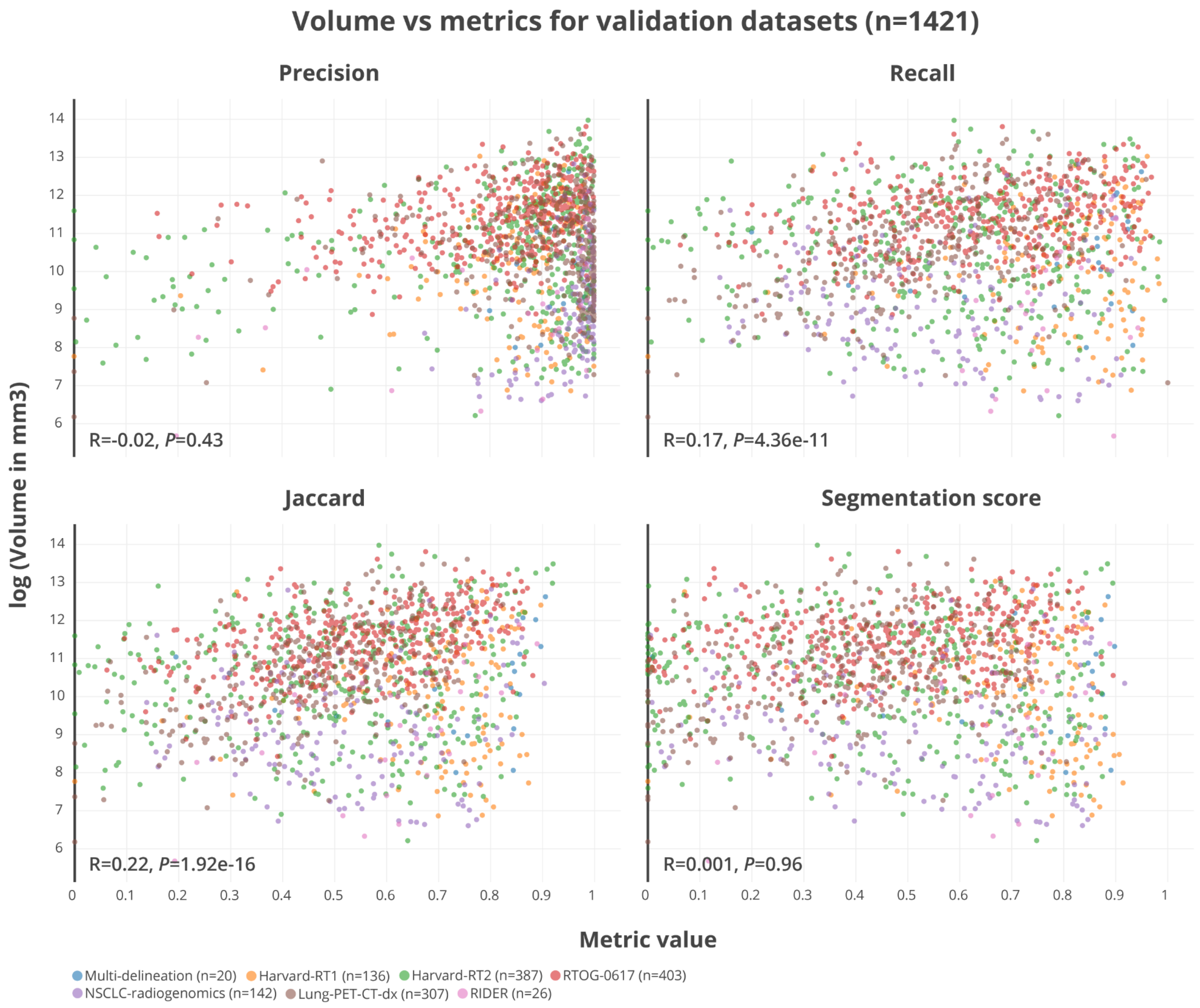

Supplementary Figure 38: Figure depicting correlation between volume and model performance across all validation datasets (n=1421). Performance metrics shown here include precision, recall, jaccard, and segmentation score. The Spearman rank-order correlation coefficient was used for measuring correlation between the two groups.

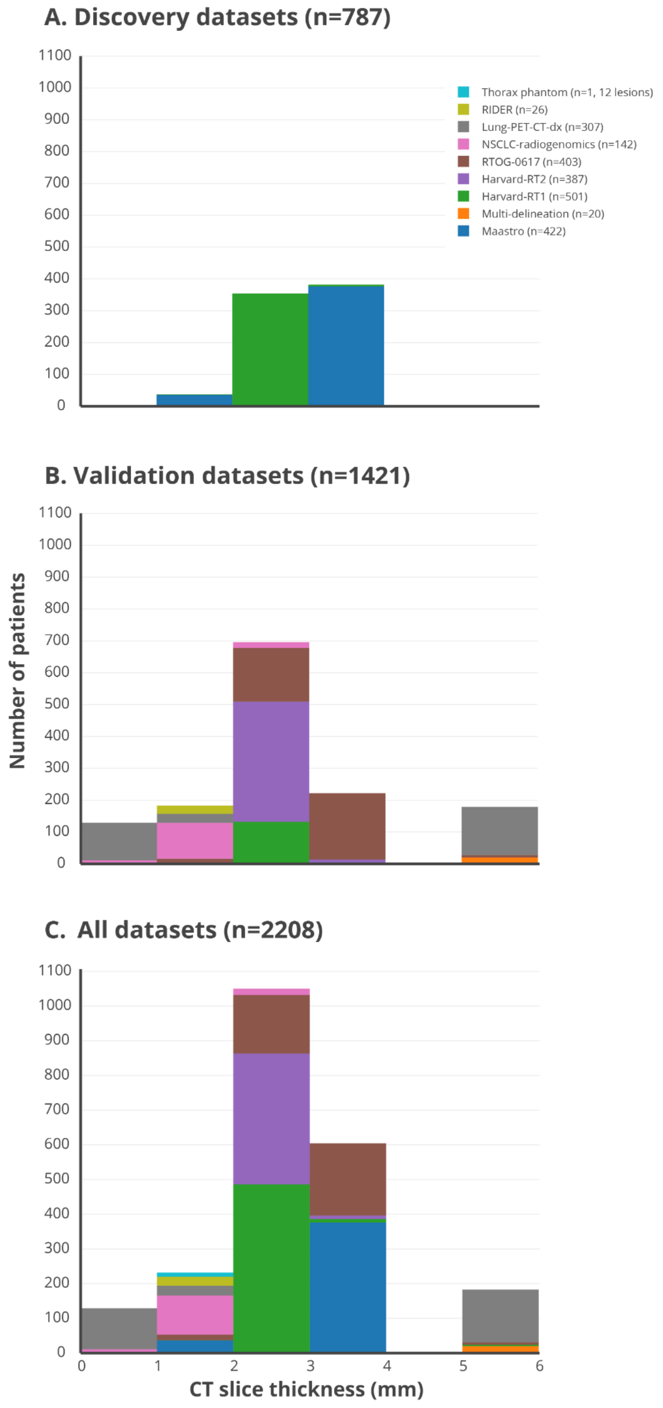

Supplementary Figure 39: Figure depicting the distribution of CT slice thickness across discovery (panel A) and validation (panel B) datasets, as well as all datasets (panel C).

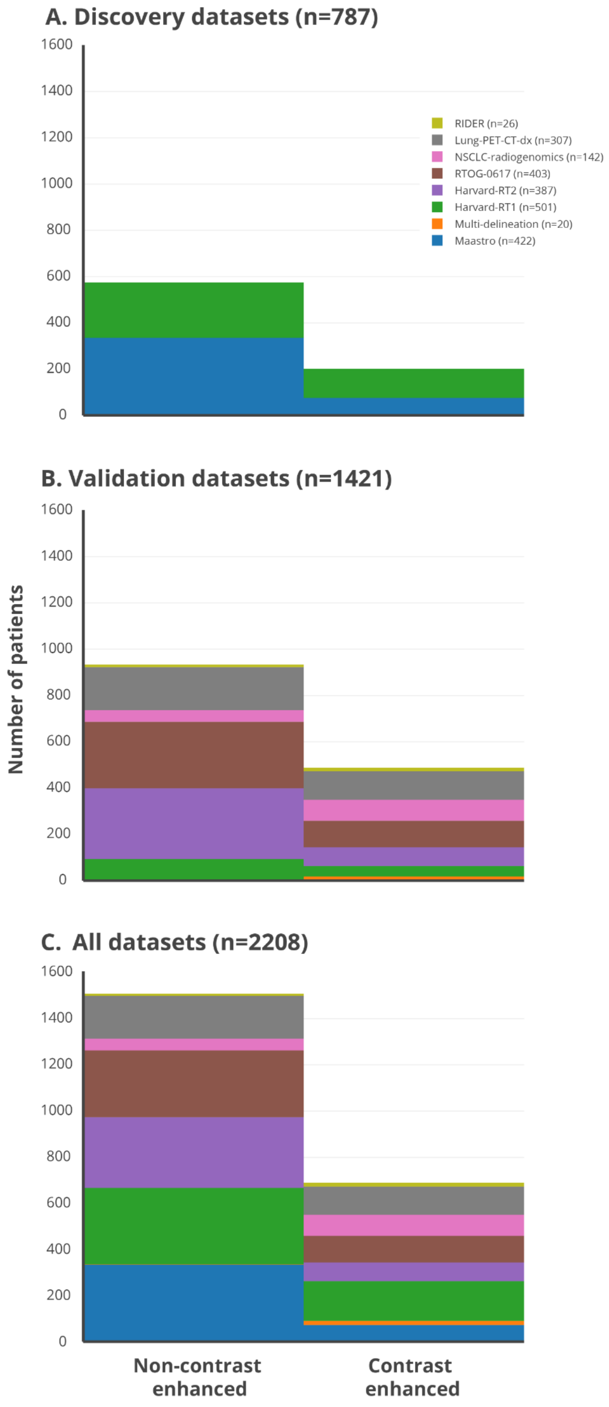

Supplementary Figure 40: Figure depicting the distribution of non-contrast and contrast CT images across discovery (panel A) and validation (panel B) datasets, as well as all datasets (panel C). Contrast labels were not readily available for all datasets. To generate this data, we used an internally-developed and unpublished contrast prediction model. This model's performance in detecting contrast was AUC 0.98 on the RTOG-0617 dataset.

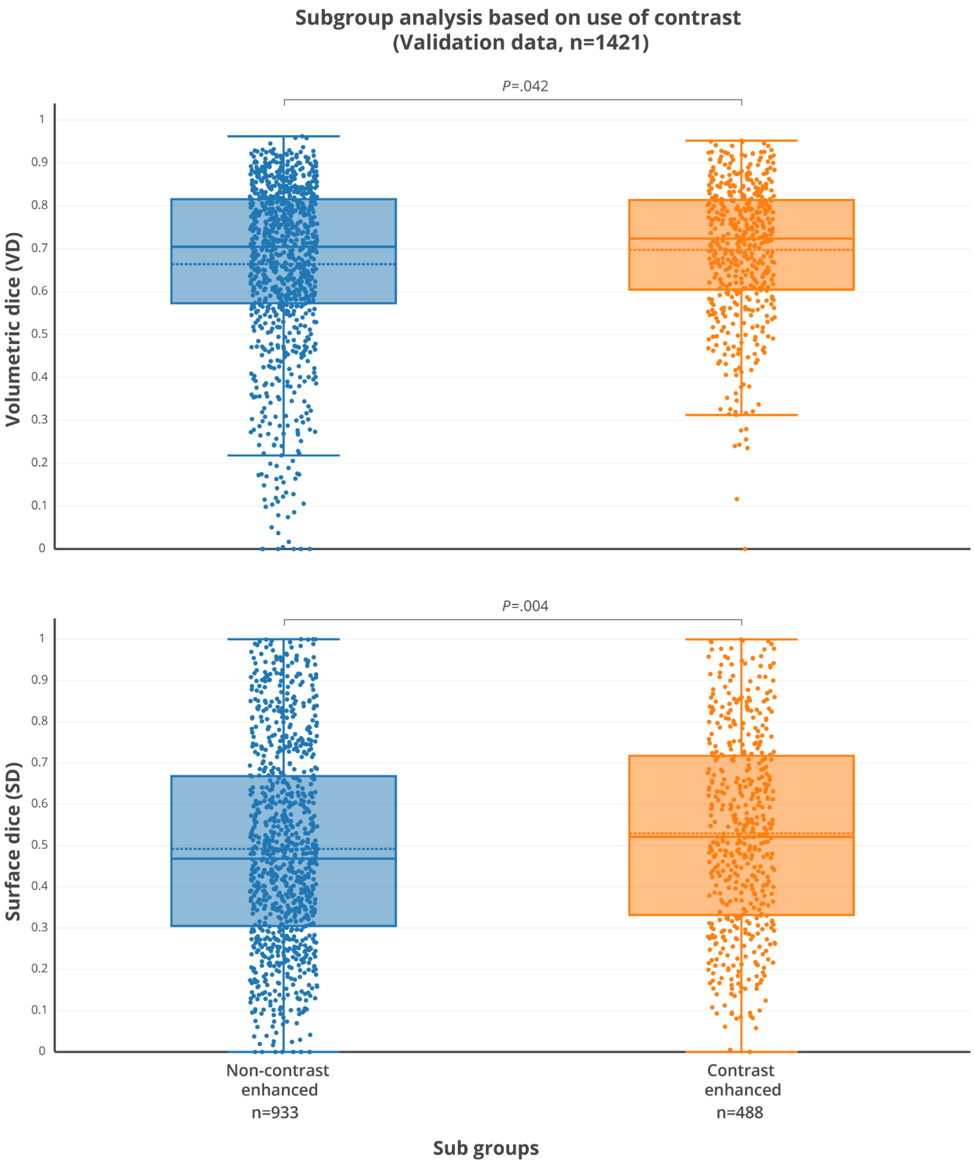

Supplementary Figure 41: Figure depicting difference in segmentation model performance between non-contrast and contrast enhanced images across all validation datasets (n=1421). Data is shown for volumetric dice (top) and surface dice (bottom) metrics. The Mann-Whitney U rank test was used, with a two-tailed $P < .05$ indicating significance.

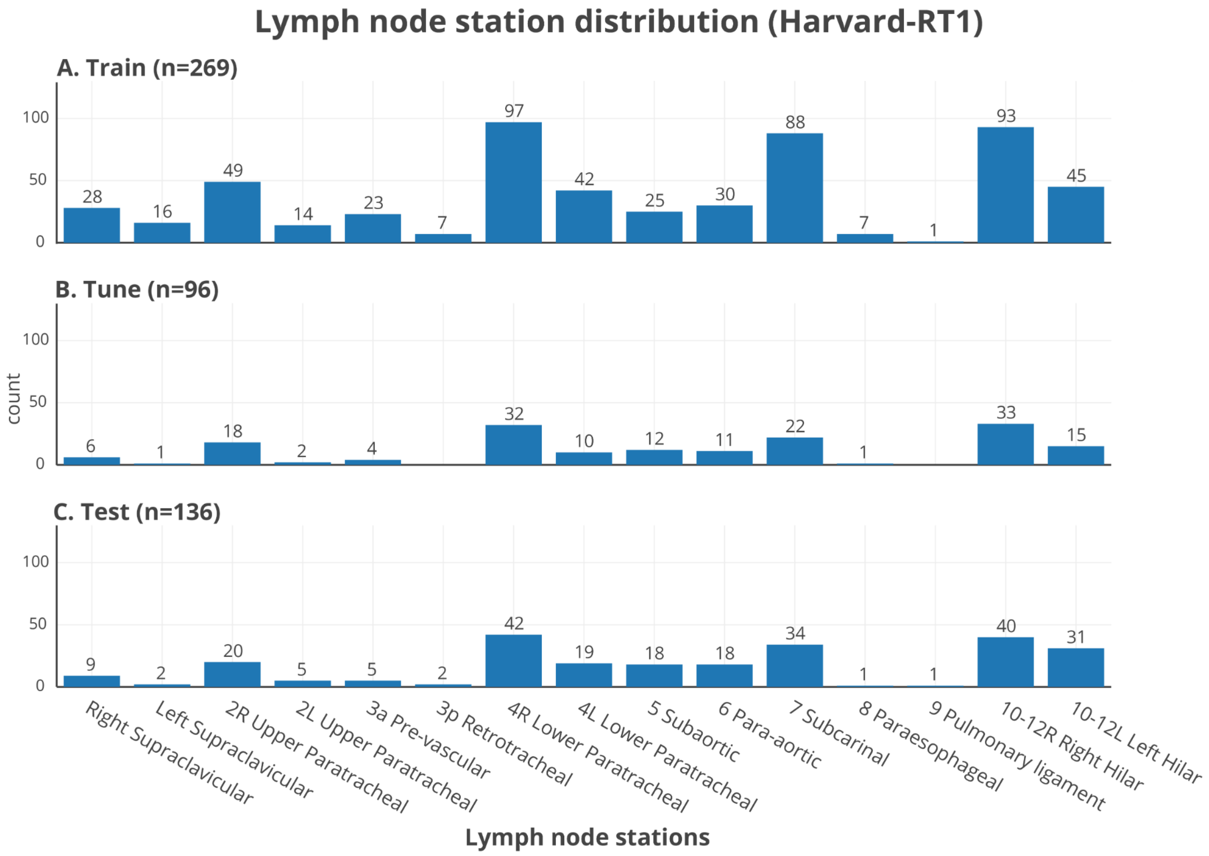

Supplementary Figure 42: Distribution of lymph node stations across the Harvard-RT1 dataset split across the training (n=269), tuning (n=96), and testing (n=136) cohorts. Patients can have multiple involved nodal stations.

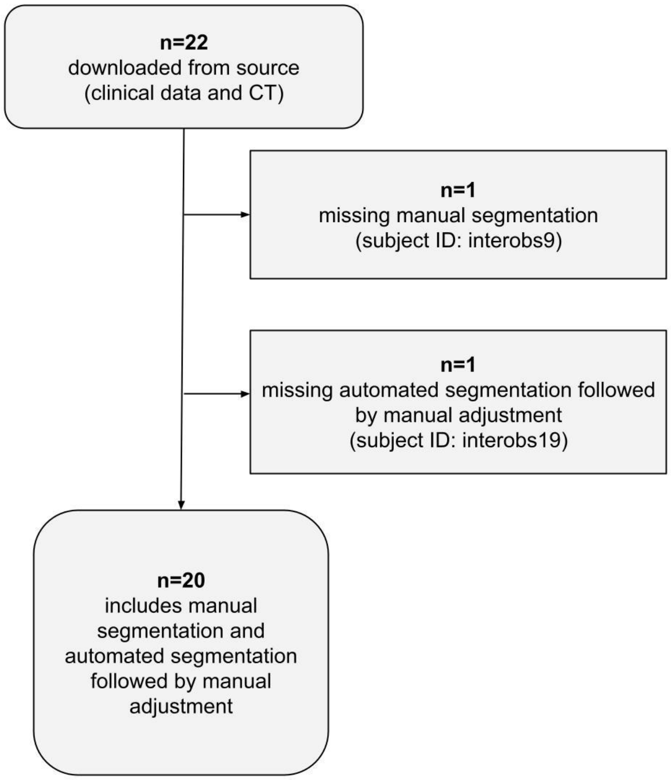

Supplementary Figure 43: Exclusion flowchart of the Multi-delineation dataset[2].

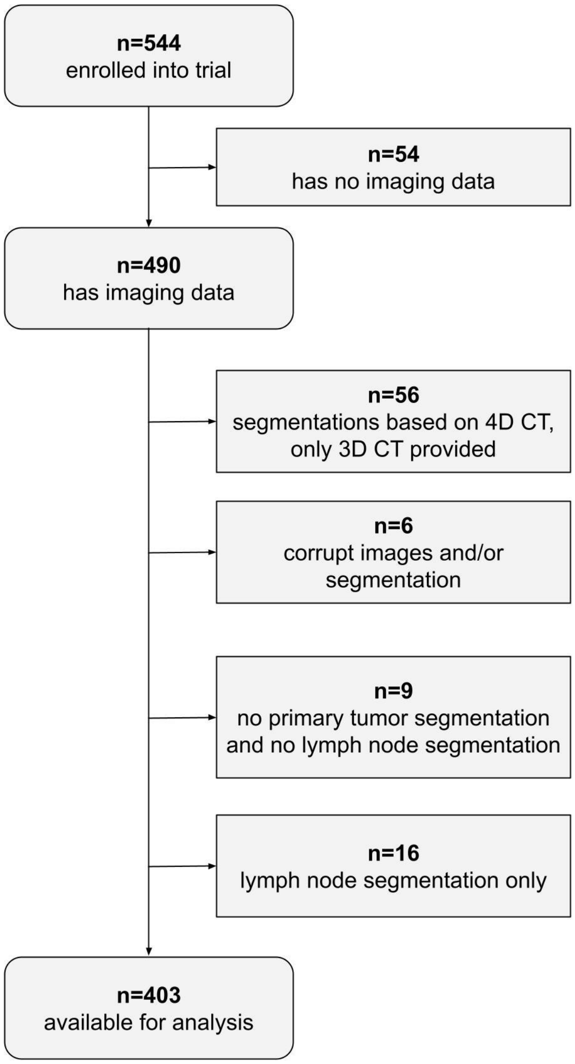

Supplementary Figure 44: Exclusion flowchart of the RTOG-0617 dataset[3].

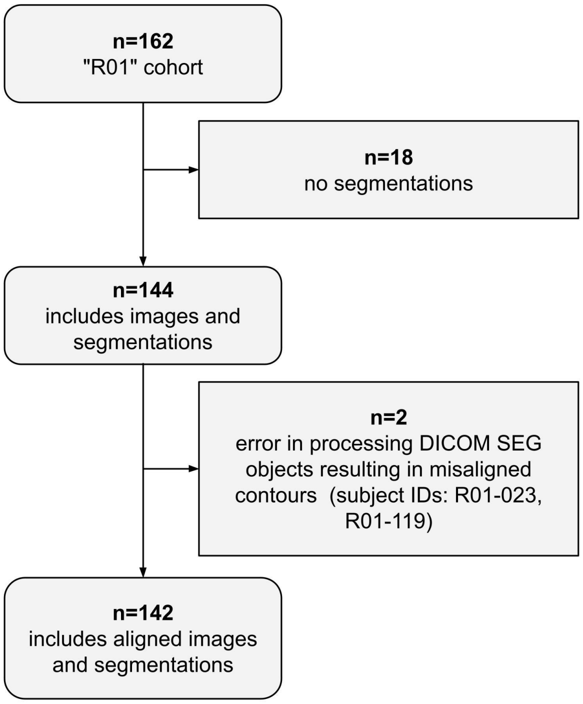

Supplementary Figure 45: Exclusion flowchart of the NSCLC-radiogenomics dataset[5].

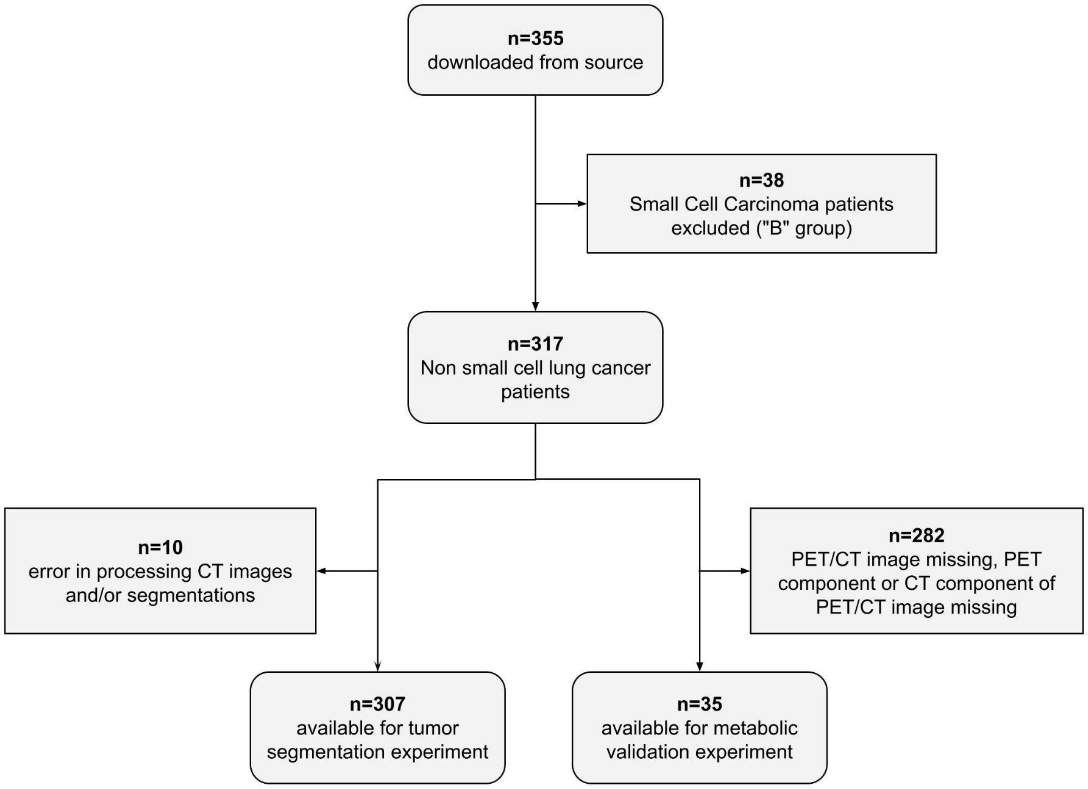

Supplementary Figure 46: Exclusion flowchart of the Lung-PET-CT-Dx dataset[26].

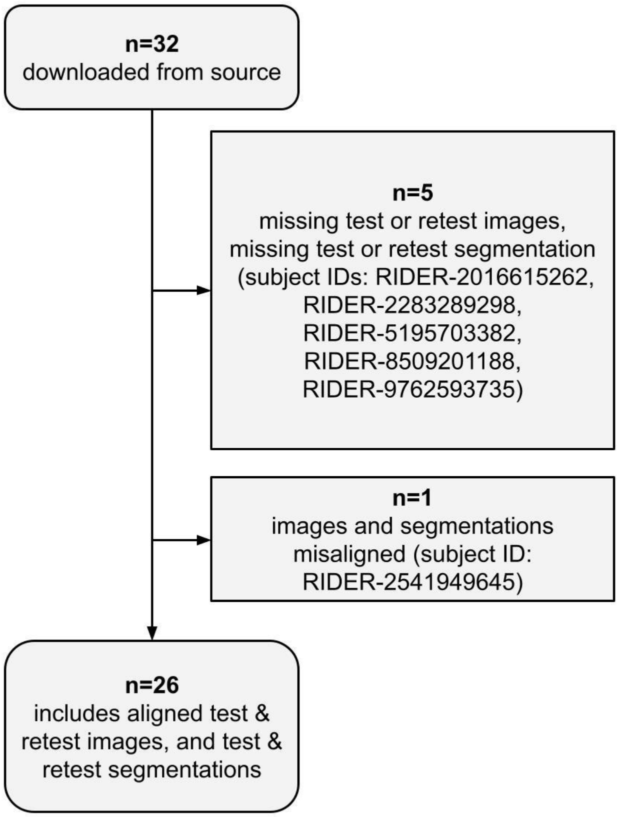

Supplementary Figure 47: Exclusion flowchart of the RIDER dataset[7].

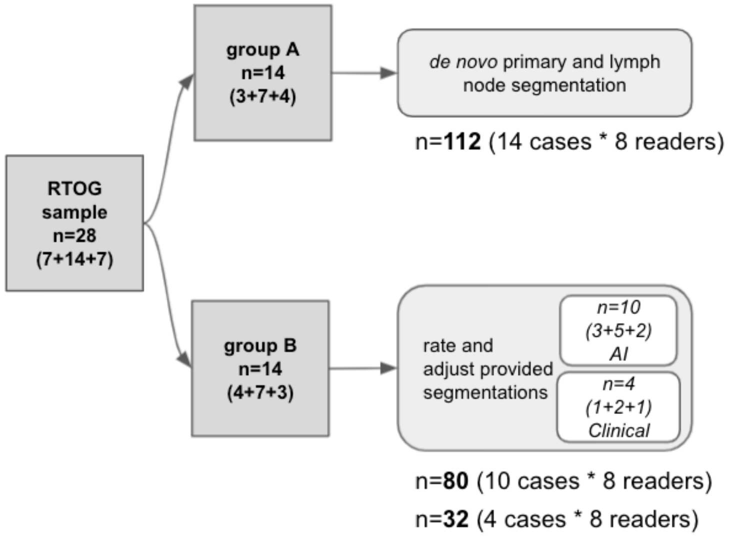

Supplementary Figure 48: Diagram depicting the data selection strategy for the end-user testing. Data consisted of a random quartile-based 28 patient subset of the RTOG-0617 clinical trial dataset. This subset was further divided into two random quartile-based groups of 14 patients each. For group A patients, readers were asked to perform the primary tumor and lymph node segmentation task de novo. For group B patients, readers were asked to rate and adjust a provided segmentation blinded to its source. For 10 patients, AI-generated segmentations were provided. For 4 patients, clinical segmentations (from RTOG-0617 clinical trial) were provided. The three numbers within brackets refer to the number of cases selected from the first quartile, interquartile range, and fourth quartile respectively. Percentiles are based on model performance (volumetric dice agreement between clinical and AI segmentations). See Supplementary table 4.

De novo segmentations (group A)

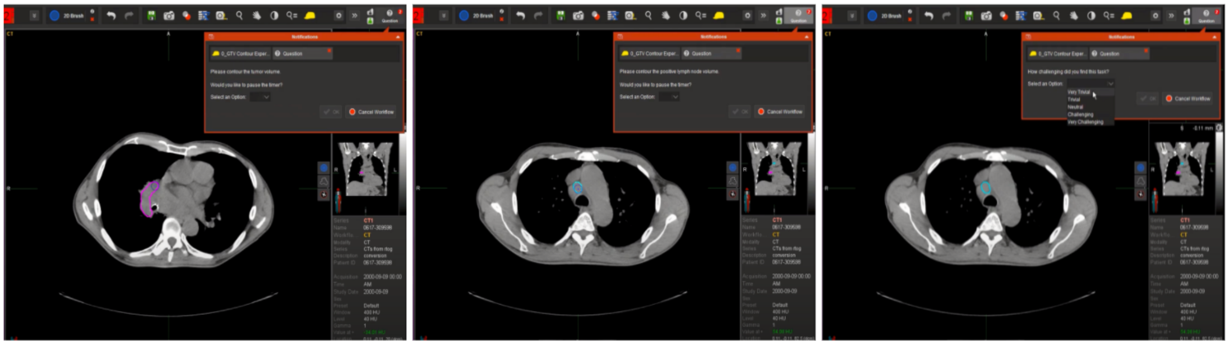

1. *De novo* primary tumor segmentation with background timing (pause option provided)

2. *De novo* lymph node segmentation with background timing (pause option provided)

3. Post-completion survey questions

Rate and adjust segmentations (group B)

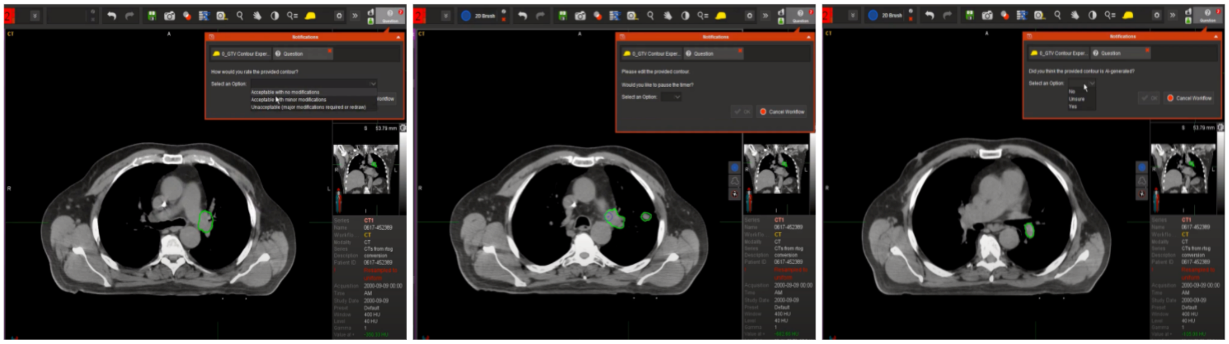

1. Rate provided segmentation

2. Adjust provided segmentation with background timing (pause option provided)

3. Post-completion survey questions including turing test

Supplementary Figure 49: Screenshots from the simulated clinical setting (MIM workflow) depicting both the *de novo* segmentation and rate-adjust segmentation workflows. During timed segmentation tasks, a pause option is provided to participants in case of interruptions. All segmentations and survey responses are saved automatically in the background.

References

1 Mak RH, Endres MG, Paik JH, *et al.* Use of Crowd Innovation to Develop an Artificial Intelligence–Based Solution for Radiation Therapy Targeting. JAMA Oncology. 2019; **5**: 654.

2 van Baardwijk A, Bosmans G, Boersma L, *et al.* PET-CT–Based Auto-Contouring in Non–Small-Cell Lung Cancer Correlates With Pathology and Reduces Interobserver Variability in the Delineation of the Primary Tumor and Involved Nodal Volumes. International Journal of Radiation Oncology*Biology*Physics. 2007; **68**: 771–8.

3 Bradley JD, Paulus R, Komaki R, *et al.* Standard-dose versus high-dose conformal radiotherapy with concurrent and consolidation carboplatin plus paclitaxel with or without cetuximab for patients with stage IIIA or IIIB non-small-cell lung cancer (RTOG 0617): a randomised, two-by-two factorial phase 3 study. *Lancet Oncol* 2015; **16**: 187–99.

4 High-Dose or Standard-Dose Radiation Therapy and Chemotherapy With or Without Cetuximab in Treating Patients With Newly Diagnosed Stage III Non-Small Cell Lung Cancer That Cannot Be Removed by Surgery. https://clinicaltrials.gov/ct2/show/NCT00533949 (accessed June 22, 2021).

5 Bakr S, Gevaert O, Echegaray S, *et al.* A radiogenomic dataset of non-small cell lung cancer. *Sci Data* 2018; **5**: 180202.

6 A large-scale CT and PET/CT dataset for lung cancer diagnosis (lung-PET-CT-dx) - the cancer imaging archive (TCIA) public access - cancer imaging archive wiki. https://wiki.cancerimagingarchive.net/pages/viewpage.action?pageId=70224216 (accessed July 9, 2021).

7 Zhao B, James LP, Moskowitz CS, *et al.* Evaluating Variability in Tumor Measurements from Same-day Repeat CT Scans of Patients with Non–Small Cell Lung Cancer. *Radiology* 2009; **252**: 263–72.

8 Kalpathy-Cramer J, Zhao B, Goldgof D, *et al.* A Comparison of Lung Nodule Segmentation Algorithms: Methods and Results from a Multi-institutional Study. *J Digit Imaging* 2016; **29**: 476–87.

9 Zhao B, Tan Y, Tsai WY, Schwartz LH, Lu L. Exploring Variability in CT Characterization of Tumors: A Preliminary Phantom Study. *Transl Oncol* 2014; **7**: 88–93.

10 Phantom FDA - the cancer imaging archive (TCIA) public access - cancer imaging archive wiki. https://wiki.cancerimagingarchive.net/display/Public/Phantom+FDA (accessed July 2, 2021).

11 Ye Z, Qian JM, Hosny A, *et al.* Deep learning-based detection of intravenous contrast in computed tomography scans. arXiv [eess.IV]. 2021; published online Oct 16. http://arxiv.org/abs/2110.08424.

12 Isensee F, Jäger P, Wasserthal J, *et al.* batchgenerators—a python framework for data augmentation. *Zenodo https://doi org/10 5281/zenodo* 2020; **3632567**.

13 Isensee F, Jaeger PF, Kohl SAA, Petersen J, Maier-Hein KH. nnU-Net: a self-configuring

method for deep learning-based biomedical image segmentation. *Nat Methods* 2021; **18**: 203–11.

14 Ronneberger O, Fischer P, Brox T. U-Net: Convolutional Networks for Biomedical Image Segmentation. In: Medical Image Computing and Computer-Assisted Intervention – MICCAI 2015. Springer, Cham, 2015: 234–41.

15 Çiçek Ö, Abdulkadir A, Lienkamp SS, Brox T, Ronneberger O. 3D U-Net: Learning Dense Volumetric Segmentation from Sparse Annotation. In: Medical Image Computing and Computer-Assisted Intervention – MICCAI 2016. Springer International Publishing, 2016: 424–32.

16 Ulyanov D, Vedaldi A, Lempitsky V. Instance Normalization: The Missing Ingredient for Fast Stylization. arXiv [cs.CV]. 2016; published online July 27. http://arxiv.org/abs/1607.08022.

17 Maas AL, Hannun AY, Ng AY, Others. Rectifier nonlinearities improve neural network acoustic models. In: Proc. icml. Citeseer, 2013: 3.

18 Mishra P, Sarawadekar K. Polynomial Learning Rate Policy with Warm Restart for Deep Neural Network. In: TENCON 2019 - 2019 IEEE Region 10 Conference (TENCON). 2019: 2087–92.

19 Jadon S. A survey of loss functions for semantic segmentation. In: 2020 IEEE Conference on Computational Intelligence in Bioinformatics and Computational Biology (CIBCB). 2020: 1–7.

20 Paszke A, Gross S, Massa F, *et al.* PyTorch: An Imperative Style, High-Performance Deep Learning Library. In: Wallach H, Larochelle H, Beygelzimer A, d\textquotesingle Alché-Buc F, Fox E, Garnett R, eds. Advances in Neural Information Processing Systems. Curran Associates, Inc., 2019. https://proceedings.neurips.cc/paper/2019/file/bdbca288fee7f92f2bfa9f7012727740-Paper.pdf.

21 Kikinis R, Pieper SD, Vosburgh KG. 3D Slicer: A Platform for Subject-Specific Image Analysis, Visualization, and Clinical Support. In: Jolesz FA, ed. Intraoperative Imaging and Image-Guided Therapy. New York, NY: Springer New York, 2014: 277–89.

22 Pinter C, Lasso A, Wang A, *et al.* Performing radiation therapy research using the open-source SlicerRT toolkit. In: World Congress on Medical Physics and Biomedical Engineering, June 7-12, 2015, Toronto, Canada. Springer International Publishing, 2015: 622–5.

23 Nikolov S, Blackwell S, Zverovitch A, *et al.* Clinically Applicable Segmentation of Head and Neck Anatomy for Radiotherapy: Deep Learning Algorithm Development and Validation Study. *J Med Internet Res* 2021; **23**: e26151.

24 Aerts HJWL, Velazquez ER, Leijenaar RTH, *et al.* Decoding tumour phenotype by noninvasive imaging using a quantitative radiomics approach. *Nat Commun* 2014; **5**: 4006.

25 van Baardwijk A, Bosmans G, Boersma L, *et al.* PET-CT-based auto-contouring in non-small-cell lung cancer correlates with pathology and reduces interobserver variability in the delineation of the primary tumor and involved nodal volumes. *Int J Radiat Oncol Biol*

Phys 2007; **68**: 771–8.

26 A large-scale CT and PET/CT dataset for lung cancer diagnosis (lung-PET-CT-dx) - the cancer imaging archive (TCIA) public access - cancer imaging archive wiki. https://wiki.cancerimagingarchive.net/pages/viewpage.action?pageId=70224216 (accessed July 9, 2021).
